# Development and application of a health-based framework for informing regulatory action in relation to exposure of microplastic particles in California drinking water

**DOI:** 10.1186/s43591-022-00030-6

**Published:** 2022-05-25

**Authors:** Scott Coffin, Hans Bouwmeester, Susanne Brander, Pauliina Damdimopoulou, Todd Gouin, Ludovic Hermabessiere, Elaine Khan, Albert A. Koelmans, Christine L. Lemieux, Katja Teerds, Martin Wagner, Stephen B. Weisberg, Stephanie Wright

**Affiliations:** 1 California State Water Resources Control Board, Sacramento, CA USA; 2 Division of Toxicology, Wageningen University & Research, Wageningen, Netherlands; 3 Fisheries, Wildlife, and Conservation Sciences Dept, Coastal Oregon Marine Experiment Station, Oregon State University, Newport, OR USA; 4 Division of Obstetrics and Gynecology, Department of Clinical Science, Intervention and Technology, Karolinska Institutet and Karolinska University Hospital Huddinge, 14186 Stockholm, Sweden; 5 TG Environmental Research, Sharnbrook, MK44 1PL UK; 6 Department of Ecology and Evolutionary Biology, University of Toronto, Toronto, ON Canada; 7 California Office of Environmental Health and Hazard Assessment, Sacramento, CA USA; 8 Aquatic Ecology and Water Quality Management Group, Wageningen University & Research, Wageningen, Netherlands; 9 Safe Environments Directorate, Health Canada, Water and Air Quality Bureau, Ottawa, ON Canada; 10 Department of Animal Sciences, Human and Animal Physiology, Wageningen University & Research, Wageningen, Netherlands; 11 Norwegian University of Science & Technology, Trondheim, Norway; 12 Southern California Coastal Water Research Project, Costa Mesa, CA USA; 13 Environmental Research Group, School of Public Health, Imperial College London, Sir Michael Uren Hub, 86 Wood Lane, London, W12 0BZ UK

**Keywords:** Microplastics, Nanoplastics, Human health, Risk assessment, Drinking water

## Abstract

Microplastics have been documented in drinking water, but their effects on human health from ingestion, or the concentrations at which those effects begin to manifest, are not established. Here, we report on the outcome of a virtual expert workshop conducted between October 2020 and October 2021 in which a comprehensive review of mammalian hazard studies was conducted. A key objective of this assessment was to evaluate the feasibility and confidence in deriving a human health-based threshold value to inform development of the State of California’s monitoring and management strategy for microplastics in drinking water. A tiered approach was adopted to evaluate the quality and reliability of studies identified from a review of the peer-reviewed scientific literature. A total of 41 in vitro and 31 in vivo studies using mammals were identified and subjected to a Tier 1 screening and prioritization exercise, which was based on an evaluation of how each of the studies addressed various quality criteria. Prioritized studies were identified largely based on their application and reporting of dose–response relationships. Given that methods for extrapolating between in vitro and in vivo systems are currently lacking, only oral exposure in vivo studies were identified as fit-for-purpose within the context of this workshop. Twelve mammalian toxicity studies were prioritized and subjected to a Tier 2 qualitative evaluation by external experts. Of the 12 studies, 7 report adverse effects on male and female reproductive systems, while 5 reported effects on various other physiological endpoints. It is notable that the majority of studies (83%) subjected to Tier 2 evaluation report results from exposure to a single polymer type (polystyrene spheres), representing a size range of 0.040 to 20 µm. No single study met all desired quality criteria, but collectively toxicological effects with respect to biomarkers of inflammation and oxidative stress represented a consistent trend. While it was possible to derive a conservative screening level to inform monitoring activities, it was not possible to extrapolate a human–health-based threshold value for microplastics, which is largely due to concerns regarding the relative quality and reliability of current data, but also due to the inability to extrapolate data from studies using monodisperse plastic particles, such as polystyrene spheres to an environmentally relevant exposure of microplastics. Nevertheless, a conservative screening level value was used to estimate a volume of drinking water (1000 L) that could be used to support monitoring activities and improve our overall understanding of exposure in California’s drinking water. In order to increase confidence in our ability to derive a human–health-based threshold value in the future, several research recommendations are provided, with an emphasis towards strengthening how toxicity studies should be conducted in the future and an improved understanding of human exposure to microplastics, insights critically important to better inform future risk assessments.

**Graphical abstract:**

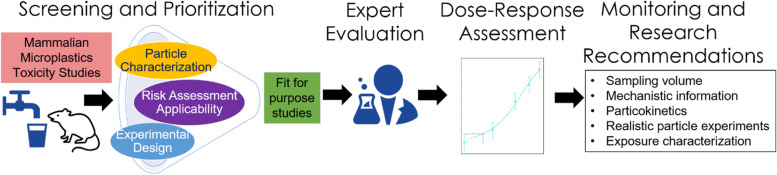

**Supplementary Information:**

The online version contains supplementary material available at 10.1186/s43591-022-00030-6.

## Introduction

Contaminants of emerging concern (CECs) identified in drinking water are often perceived to be public health risks prior to the acquisition of reliable hazard and exposure data needed to sufficiently evaluate their potential human health implications (Anadu and Harding [4]). It is important that confidence in the quality of drinking water is maintained at a high level and that actions to address potential concerns with respect to human health risks regarding exposure to CECs are thoroughly addressed. Failing to address public concern can result in negative societal impacts, such as a decreased trust in government organizations (Anadu and Harding [4]; Molak et al., [74]) as well as increased consumption of bottled water and sweetened beverages, which are linked to sustainability, social equity, and human health concerns (Pacheco et al., [84]; Cohen and Ray [22]).

Despite limited understanding of the human health implications associated with exposure to microplastic particles (MPs) (Vethaak and Legler [112], WHO [120], SAPEA [59]), which have been reported in drinking water samples collected from tap water sources from various municipalities in the United States (Kosuth et al., [71]), there exists a public perception of human health risk from MPs exposure (Thiele and Hudson [107]; Catarino et al., [19]). This perception has prompted a number of regulatory initiatives. Recent initiatives taken by the State of California include a requirement to monitor for MPs in drinking water, with the aim being to implement a reporting system that will appropriately inform consumers of their exposure to MPs via drinking water and demonstrate that appropriate actions are being taken to ensure negligible risk to consumers (California Code of Regulations, [18]). MPs are defined here according to California’s legal definition, which includes solid polymeric particles with or without additives between 1 nm and 5000 µm, and excludes natural polymers that have not been chemically modified (other than by hydrolysis) (State Water Resources Control Board, [103]).

Chief among California’s directive is the legal requirement for the State Water Resources Control Board—the agency governing drinking water—to consider adopting health-based guidance to aid consumers in interpreting concentrations of MPs in drinking water (Health and Safety Code 116376(3) [95]). Previous assessments aimed at characterizing and quantifying the human health risks of MPs have cited a paucity of reliable toxicity and exposure data relevant for drawing conclusions on the human health implications that exposure to MPs in drinking water represents (WHO [120]; SAPEA [59]). Since these assessments were published, there have been a number of toxicological studies reporting on various effects in mammalian models, primarily rodents, with relevance to human health exposure to MPs via drinking water.

California’s drinking water management framework currently includes three public health-based advisory levels for informing management and regulatory decisions: Notification Levels, Response Levels, and Public Health Goals (Fig. S1). A Screening Level is an additional, non-regulatory level that informs monitoring for CECs, and may be used when data are insufficient to derive a regulatory level, thus representing the earliest action towards addressing a CEC. The ‘gold standard’ of ‘California’s management construct’ is to reliably define and set Public Health Goals; public health goals form the basis of California’s drinking water regulatory standards. Establishing a Public Health Goal typically requires a period of extensive scrutiny, including two public comment periods and external scientific peer review (Health and Safety Code 116365 [93]). Notification Levels are health-based advisory levels established by the California Division of Drinking Water, with exceedances triggering a series of regulatory requirements, including the mandatory notification of the exceedance to drinking water consumers (State of California Health and Safety Code 116455 [94]). Response Levels are set at higher concentrations and carry additional regulatory requirements, including the possible removal of the affected system from service. The screening level thus represents a conservative, early estimate that carries no regulatory requirements and is not subject to public review. The derivation of a screening level, however, may be used to estimate analytical detection limits that are required to confidently measure concentrations aligned with levels where biological effects may occur.

To inform the State Water Resources Control Board’s monitoring goals and provide a scientific foundation for the consideration of adopting a health-based level (e.g., notification level) for MPs in drinking water and/or other guidance to aid consumer interpretations of the results as specified by Health and Safety Code 116376(3) [95], a Microplastics Health Effects Expert Virtual Workshop was convened (“Workshop”; held October 2020—October 2021). Workshop participants were charged with evaluating the evidence for hazards presented by MPs in drinking water and providing a recommendation to the State Water Resources Control Board for the type of health-based guidance level appropriate for adoption. Participants acknowledged the nascent nature of the field, and a considerable portion of the workshop was spent constructively discussing and evaluating the available studies and their use in deriving an appropriate health-based level.

Here we report on the outcome of those discussions, which includes an assessment of available mammalian hazard studies that have been screened and prioritized as fit-for-purpose. Studies were prioritized to provide the basis for discussions, which included an evaluation of the potential hazard endpoints and an uncertainty and sensitivity analysis aimed at estimating exposure concentrations through drinking water. Further, they provide both scientific guidance for monitoring through the derivation of a preliminary health-based level, as well as recommendations for further research. This work represents one of the first attempts globally to critically assess potential health effects of microplastics to humans through drinking water.

## Method

### Data collection and screening

Studies were identified based on a literature review aimed at identifying original research relevant for use in informing a human health threshold value for MPs in drinking water. Peer-reviewed studies were identified through an original literature search which included search results from Gouin et al. [42] and was expanded to include additional studies published through June 1, 2021. Studies were identified using the ProQuest database, Google Scholar, and PubMed search engines using the keywords ‘microplastic(s)’ AND ‘toxicity’. Additional studies were identified through the reference list in the United Kingdom Health and Safety Executive report summarizing hazards and risks of fibers (Burdett and Bard 2007) [15], and literature review by Rahman et al. (2021) [92] and Wright and Kelly (2017) [122]. While every effort way made to identify all relevant studies, we acknowledge that this approach may not be comprehensive and that some studies could have been missed based on how the literature search was performed, specifically due to the keyword ‘microplastics’ only being in use since ~ 2004 (Thompson et al. [108]). Information from studies was extracted manually and compiled in an interactive web-based RShiny application database “ToMEx” (Thornton Hampton L, Lowman H, Coffin S, Darin E, De Frond H, Hermabessiere L, et al: A Living Tool for the Continued Exploration of Microplastic Toxicity, In Press), from which summary statistics are reported and evaluation scores against various quality assurance and quality control (QA/QC) criteria can be obtained (Gouin et al., [42]). Both in vivo and in vitro studies were identified. While methods exist for quantitatively extrapolating from in vitro to in vivo systems for soluble contaminants (e.g., predicting estrogenic potencies of bisphenol A—Punt et al., [89]), methods are currently under development for insoluble (i.e. particulate) contaminants (Romeo et al., [97]), and while useful as screening tools for potential effects, in vitro studies cannot (yet) be unconditionally used for human health risk assessment (Noventa et al., [77]). Accordingly, only in vivo studies were screened and prioritized for further assessment.

In vivo mammalian studies identified were screened and prioritized according to criteria evaluating particle characterization, experimental design, and applicability for use in risk assessment (Table S3, replicated from Gouin et al., [42]). A diagram detailing the identification, screening, and prioritization of microplastics studies illustrates the workflow employed here (Fig. S2). Consistent with the approach and recommendations made by Hermsen et al. [45], Koelmans et al. [57] and de Ruijter et al. [28], each quality criterion is scored as adequate (2), adequate with restrictions (1) or inadequate (0). Under this approach, studies without zero scores are considered applicable for risk assessment without additional review (de Ruijter et al., [28]; Hermsen et al., [45]; Koelmans et al., [57]). However, in their evaluation of mammalian toxicity studies relevant for deriving a human health threshold value for drinking water, Gouin et al. [42] report that no studies received non-zero scores against all criteria. Thus, in a pragmatic approach, a more lenient set of 14 quality criteria were identified by Workshop participants as representing the minimal set of data required for conducting a preliminary assessment, with the understanding of the significant limitations of the findings. Studies receiving a minimal score of ‘1’ (adequate with restrictions) against each of the selected set of quality criteria were prioritized for further evaluation. The selected set of quality criteria, referred here as ‘red criteria’ include: a) four criteria aligned with particle characterization, whereby studies are required to report the size, shape, polymer type and the source of the particles; b) seven criteria aligned with study design, with a requirement that studies should, as a minimal, report the test medium and/or delivery vehicle, the administered dose, the administration route, the test species, the number of animals used (i.e. sample size), the frequency and duration of the exposure, and the use of controls; and c) three criteria aligned with applicability for risk assessment, including the reporting of toxicological endpoints, a dose–response relationship and the effect threshold. However, we emphasize that the omitted criteria are as crucial as the red criteria and that the only reason for their omission is that otherwise an insufficient number of data would be available.

### External expert consultation

Recognizing that the toxicity data and endpoints for studies prioritized include a number of underlying challenges (for example, remaining concerns associated with those criteria not used for screening purposes, i.e. non-red criteria), as well as the need for a thorough assessment regarding the reliability of specific endpoints), a tiered approach was used. The screening and prioritization tool described by Gouin et al. [42] represents the Tier 1 level of evaluation, aimed largely at assessing the relative applicability of the data for risk assessment. The objective of the Tier 2 expert evaluation is to provide additional information regarding the relative reliability of various specific endpoints, in particular those associated with mammalian reproductive toxicity. Since the expertise of the workshop participants did not include individuals with specific expertise in the various endpoints reported in studies prioritized, external experts were solicited to help inform a greater understanding of the relative extent of the uncertainties associated with the data reported in each of the studies.

The application of the tiered approach suggested above has similarities to the use of expert knowledge elicitation, which is understood to represent an important step in the analysis of risk. For instance, in evaluating the potential risk associated with dietary exposure to a CEC, there are several questions that risk assessors need to address, such as the characterization of the hazard (i.e. genotoxic/non-genotoxic), as well as the toxicokinetics and toxicodynamics (i.e. dose–response in relation to a target organ) of the CEC (European Food Safety Authority [38]). In the instance of MPs, where the knowledge of both the hazard and the exposure are associated with various sources of uncertainty (SAPEA [59], WHO [120]), the robustness of the evidence base for enabling an assessment of risk is critically important. The adoption of the tiered approach as part of this activity thus represents a process similar to the use of expert knowledge elicitation used by various groups, such as the European Food Safety Authority (European Food Safety Authority [38]) in their approach to assessing the risk of CECs in food and for supporting the environmental risk assessment process by the United States Environmental Protection Agency (US EPA [37]).

Following guidance provided by the European Food Safety Authority [38] and the US EPA [37], external experts were selected based on their expertise in their respective fields of study. Subject-matter experts having expertise relevant to specific toxicological endpoints reported in studies prioritized for evaluation (e.g., mammalian reproductive toxicity, mammalian gut toxicity, etc.) were asked to evaluate the human relevance and adequacy of the biological response measurements. The process employed for choosing expert reviewers is similar to the methodology utilized by journal editors, which relies on a combination of expertise reputation, availability, and personal contacts. A total of eight experts provided assessments, representing various technical expertise. Each of the prioritized studies was reviewed by at least one outside expert. Due to various logistical challenges (i.e. time constraints of experts, incompatible expertise with all prioritized studies), not all external experts reviewed all studies, resulting in an inherent inconsistency with respect to the number of reviews aligned with each of the prioritized studies. Consequently, while the overall approach adopted here is acknowledged as being inappropriate for enabling a quantitative evaluation of all studies, Workshop participants agreed that the knowledge obtained was sufficient to support a qualitative assessment identified as necessary to meet the objectives of the workshop. A summary of the 12 studies that were subject to expert evaluation is provided in Table S5, including the names and affiliations of each of the external experts who agreed to having their feedback shared in this way.

**Fig. 1:**
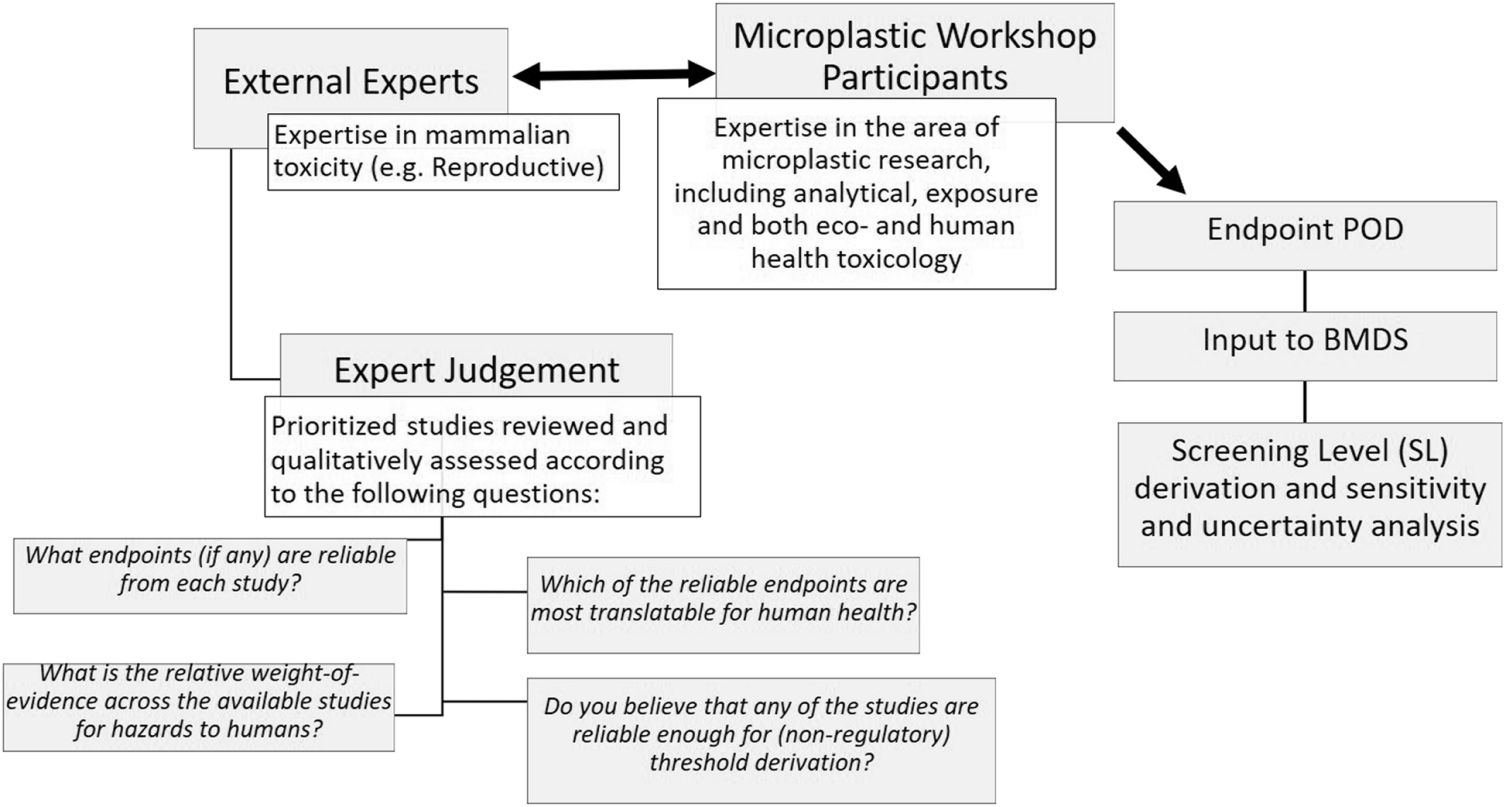
Summary of workshop process adopted towards derivation of a non-regulatory human-health screening level value for monitoring microplastics in California drinking water. BMD = benchmark dose

The objective of the expert knowledge elicitation adopted in this instance was therefore to obtain qualitative expert judgement regarding the reliability of studies prioritized from the Tier 1 screening evaluation. The feedback received from the external experts was then communicated and considered by the workshop participants, who then used this information to estimate points of departure (e.g., benchmark doses [BMDs]). BMDs are doses (or concentrations) that elicit a predetermined change in response of an adverse effect based on a modelled dose–response curve, and is the preferred method for assessing risks by many regulatory agencies such as the US EPA and the European Food Safety Authority (EFSA Scientific Committee et al., [35]). The BMD of endpoints, along with their respective upper and lower 95% confidence intervals were estimated based on modelled output using the US EPA benchmark dose software (BMDS) (US EPA [36]), which was further compared and interpreted using the RIVM PROAST tool (Slob, [101]). Figure 1 provides a schematic illustration of the process adopted.

### Benchmark dose modelling

Knowledge obtained from the external experts was considered by workshop participants, with the output of discussions collated and used as the basis for identifying endpoints for use in estimating their statistical BMD which (if BMD model is reliable) is used as a point-of-departure (Fig. 1). Benchmark dose modelling software fits mathematical models to data and determines the exposure concentration (benchmark dose or BMD) that corresponds to a predetermined response level (benchmark response or BMR). All analyzed data were continuous, and a BMR of one standard deviation from the control mean was used for all endpoints in accordance with US EPA guidance (US EPA [36]). BMDs were derived using both the US EPA BMDS (EPA, [36]) and the RIVM PROAST (Slob [101]) to examine the uncertainty of different BMD modelling methods. A thorough description of the BMD modelling methods used by both the US EPA BMDS and PROAST can be obtained from (US EPA [36]; Slob [101]). BMDs derived using US EPA’s BMDS were used in the derivation of a screening level, and uncertainties were reported using their respective upper and lower 95% confidence intervals.

Identification of the best BMD model using the US EPA BMDS was determined using a consensus approach, which included an evaluation of model goodness of fit (*p*-value > 0.10); lowest Akaike’s Information Criteria (AIC), scaled residuals < absolute value of 2, BMD/BMDL (lower 95% confidence interval) ratio < 5 and visual inspection of curve fit to assess plausibility and model parsimony (US EPA [36]). When no BMD model met the requisite criteria, the no-observed adverse effect level (NOAEL) was used to derive a point of departure (POD) normalized to body weight (mg·kg^−1^·d^−1^). In cases in which NOAELs were not reported and a BMD could not be reliably modelled, the LOAEL was divided by an assessment factor of 10 to derive the POD, according to guidance suggested by the California Office of Environmental Health and Hazard Assessment (OEHHA) (OEHHA [81]). In instances where dose–response data were not reported in tabular format, the necessary data were extracted from figures presented in the studies using an online tool (Rohatgi [96]).

### Screening level derivation

The derivation of a non-regulatory human-health screening level for MPs in drinking water was estimated using Eq. 1, which is based on the approach taken by OEHHA when deriving Public Health Goals for non-cancer effects (OEHHA [82]). Equation 1 estimates the concentration in drinking water that would not be expected to exceed a non-cancer hazard quotient of 1.0 by incorporating exposure through drinking water and other sources (OEHHA [83]). The screening level is derived using the reference dose (RfD), which is the POD divided by composite uncertainty factors (UFs).

1 $$Screening level (mg/L)=\frac{RfD (mg/kg-day) x RSC (unitless fraction)}{DWI (L/kg-day)}$$

Where RSC is a unitless fraction defining the relative source contribution (RSC) of exposure from drinking water, relative to all other exposure sources (e.g., food, ambient air, household dust), and DWI is the daily drinking water intake (DWI) for individuals in California. The US EPA Exposure Decision Tree Approach was used to derive a deterministic RSC, which considers availability of exposure data and specific subpopulations of concern (EPA, [110]). An age-specific DWI is estimated using methods described by OEHHA [83], from which data obtained from a nationwide survey of food and beverage intake for approximately 20,000 people (US Department of Agriculture’s Continuing Survey of Food Intake of Individuals 1994–1996, 1998 dataset) were used and normalized to body weight. The upper 95th percentile for the 70-y lifetime weighted average of 0.053 L·kg^−1^·d^−1^ was used as the default value (see derivation in supplemental information) (OEHHA [83]).

### Sensitivity and uncertainty analysis

The input parameters used in deriving the screening level (Eq. 1) are each represented by an inherent uncertainty and variance, the relative magnitude of which can have varying impacts on the sensitivity of the estimated value (Buser et al., [17]). Therefore, a sensitivity and uncertainty analysis aimed at characterizing and quantifying the influence of input values and their sensitivities as propagated through Eq. 1 was conducted. Table 1 reports the range of values used in the sensitivity analysis identified for each of the input parameters.

**Table 1:** Parameters^a^ used in the sensitivity analysis of the derivation of the screening level

Parameter	Intermediate value	Lower value	Upper value	Units
POD	BMD	^b^BMDL (default)	BMDU	mg·kg^−1^·d^−1^
RSC	0.20 (default)	0.00001	0.50	Unitless fraction
DWI	0.053 (upper 95^th^ %; default)	0.022 (50^th^ %)	0.135 (Maximum)	L·kg^−1^·d^−1^

^a^*POD* point-of-departure, *RSC* relative source contribution, *DWI* drinking water intake rate, *BMD* benchmark dose, *BMDL* lower 95^th^ percentile of benchmark dose, *BMDU* upper 95^th^ percentile of benchmark dose

^b^When BMD could not be reliably modelled, NOAELs were used. When a NOAEL was unavailable, the LOAEL was used divided by an uncertainty factor of 10

When available, parameters used in the sensitivity analysis in the derivation of the screening level were based on empirical data (e.g., DWI) or modelled uncertainties (e.g., BMD 95% confidence intervals). For the POD, the upper and lower 95% confidence interval values (BMDU and BMDL, respectively) can be obtained and used as a quantitative estimate of uncertainty. To arrive at the reference dose, the POD is typically divided by UFs used to account for variabilities and uncertainties unless data is available (EPA [36], OEHHA [83]). The UFs consider a number of factors, such as: extrapolating a POD between animals and humans (interspecies; UF = 10); differences in response within the population (intraspecies; UF = 10); adjustments required for converting LOAELs to NOAELs (UF = 10); and to account for database deficiencies (UF = $\sqrt{10}$). UFs are multiplied and rounded down to derive the composite UF. Accordingly, for cases in which BMDs or NOAELs are used as PODs, the maximum composite UF would be 300 (i.e., 10 × 10 x $\sqrt{10}$, rounded down), and 3000 when LOAELs are used as the POD due to the additional UF of 10 to estimate NOAELs based on LOAELs. The uncertainty introduced into the derived screening level by UFs is assessed by using a minimum composite UF of 1 (i.e., no UFs) and the maximum composite UF of 300 (or 3000 when NOAELs are used).

While MPs have been detected in household dust, air, seafood (fish, molluscs, crustaceans), salt, bottled water, beer, milk, tap water, human faeces, and placenta (Mohamed Nor et al., [73]; Zhang et al., [126]; Ragusa et al. [91]), the availability of data to obtain a comprehensive quantification of human exposure represented by these sources is currently limited due to a lack of data on food and other potentially significant exposure sources (Liu et al., [66]; Fadare et al., [39]; Dawson et al., [26]; Wright et al., [121]). Therefore, we used a default RSC of 20% consistent with the US EPA [110] guidance and tested the sensitivity of this assumption by varying the RSC from between 0.00001- 0.50, which we anticipate represents a reasonable range of extreme values aligned with scenarios whereby exposure to MPs in drinking water is either negligible or where it represents a potentially significant source of exposure, respectively. The choice of RSC ranges in this sensitivity analysis are based on estimates within the 95^th^ percentile bounds modelled by Mohamed Nor et al. [73], and the default US EPA [110] approach of varying RSCs from 0.20 to 0.80 was not employed here. As described above we have used the upper 95th percentile for the 70-yr lifetime weighted average of 0.053 L·kg^−1^·d^−1^ as the default value (OEHHA [83]). For the purposes of the sensitivity and uncertainty analysis, however, we consider the sensitivity of the DWI by using the median (0.022 L·kg^−1^·d^−1^) and maximum (0.135 L·kg^−1^·d^−1^) of the 70-yr lifetime weighted average from OEHHA [83], with additional details regarding the derivation of these values described in the supplementary information.

## Results and discussion

### Literature review and Tier 1 screening evaluation

A total of 66 mammalian toxicity studies reporting on the adverse effects of MPs were identified through the literature review, of which 31 reported results from in vivo experiments using oral exposure, and 41 reported results from in vitro mammalian cell line systems, with several studies reporting results for both in vivo and in vitro experiments (Table S1). The majority of studies (i.e., 64%) reporting on mammalian effects were published after 2019 (Fig. S3). Thus, the evaluation of MP studies presented here, with respect to the potential human health implications, is timely. A complete list of all studies, including a summary results of their evaluation against each of the screening criteria aligned with particle characteristics, experimental design, and applicability for risk assessment, is included in the supplemental information (Fig. S4).

A detailed discussion of the Tier 1 evaluation process used to inform this study is presented in Gouin et al., [42]. Noting that the availability of quantitative in vitro to in vivo extrapolation (“QIVIVE”) models are currently not available, and which represents an important research need (Romeo et al., [97]; Noventa et al., [77]; Gouin et al., [42]), the discussion presented here is thus largely limited to the results reporting dose–response relationships from mammalian in vivo studies. Specifically, the derivation of a screening level for use in estimating a health-based threshold for informing regulatory and monitoring activities is based entirely on results obtained from in vivo studies. Knowledge gained from in vitro studies, however, is considered further below in the context of evaluating potential toxicological modes of action, information from which can help inform future research activities.

**Fig. 2:**
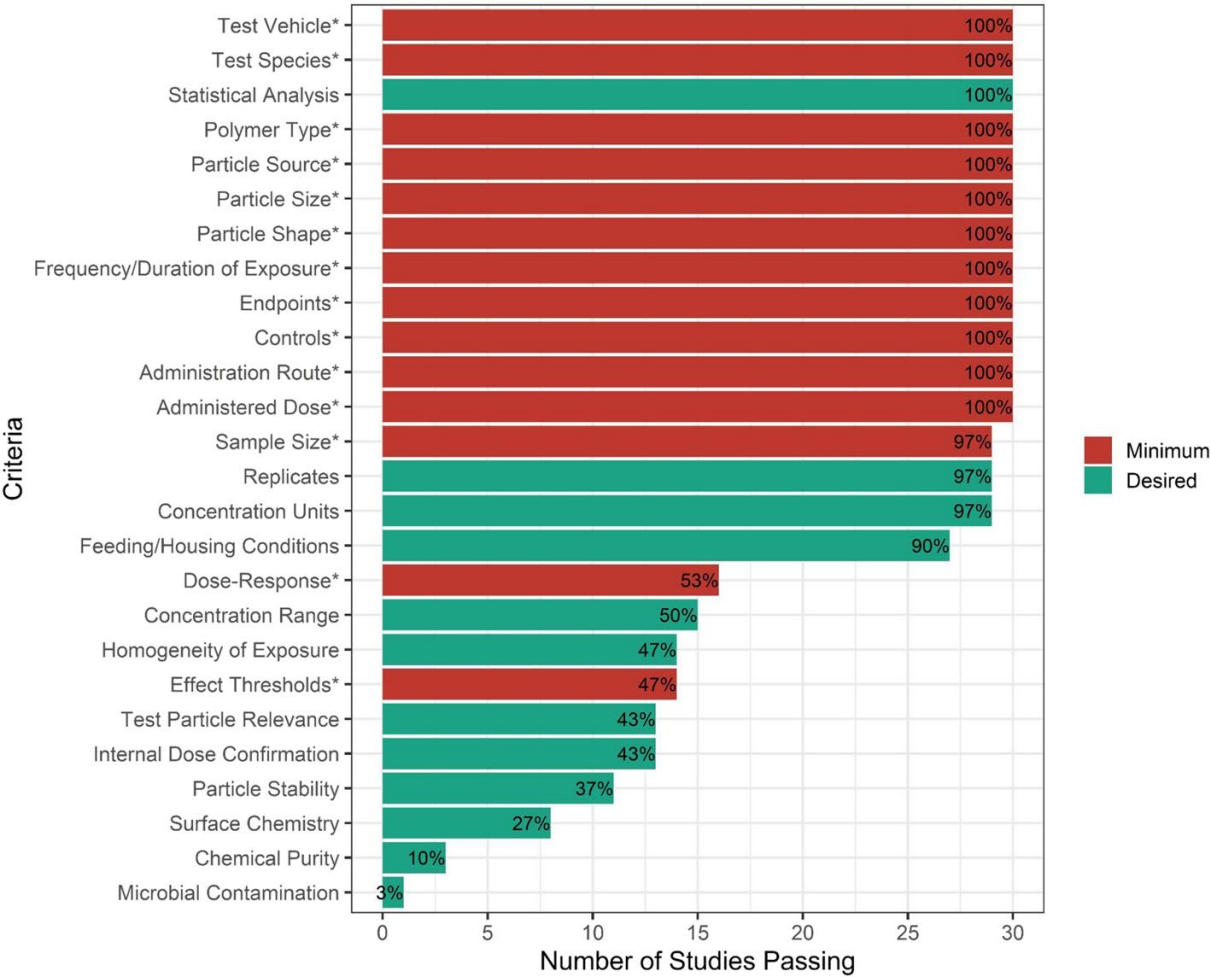
Summary of the quality screening results for all in vivo mammalian studies against 26 criteria. Prioritized studies should be assigned a score of > 0 against all criteria. In this study, however, due to lack of data, a subset of criteria has been identified as necessary for results to potentially be considered as fit-for-purpose towards helping to inform a dose–response assessment (‘red criteria’); these criteria are displayed in red and indicated by an asterisk (*), with all other screening criteria shown in green. Additional details from the Tier 1 evaluation are reported in the supplemental information (reference figure/tables) and in Gouin et al., [42]

An illustrative summary of the Tier 1 evaluation results associated with 26 screening and prioritization criteria (Gouin et al., [42]) is shown in Fig. 2. While no study received a non-zero score against all criteria, the majority of studies were assigned a score of ‘1’ (i.e., adequate with restrictions) or greater against most of the ‘red criteria’. Importantly, however, only 53% of in vivo studies report effects that were dose-dependent, and only 47% report a statistical effect threshold (e.g., LOEC). Thus, the limiting factor influencing the prioritization of studies as fit-for-purpose in the context of this study are strongly influenced by how well they addressed those two criteria. Additionally, the majority of studies failed non- ‘red criteria’ pertaining to particle characterization (e.g., ensuring chemical purity and lack of microbial contamination), indicating the significant uncertainties associated with their findings that are discussed in detail in Gouin et al. [42]. The screening and prioritization tier results indicate that 12 studies are potentially fit-for-purpose for this study, largely because they report an appropriate dose–response (or concentration–response) relationship and include sufficient data to derive an effect threshold, while also addressing the selected set of screening criteria (Table 2).

**Table 2:** Summary of in vivo ingestion-based mammalian studies prioritized for Tier 2 expert evaluation

Study and DOI	Particle type and shape	Particle length (μm)	Exposure Concentrations (mg/kg bw/day)	Exposure Method and Matrix	Tissues Investigated	Number and type of endpoints reported^a^
***Male reproduction***						
Hou et al. [47] 10.1016/j.jhazmat.2020.124028	Polystyrene Spheres	5	0.017, 0.17, 1.73^b^	Drinking water Deionized water	Testis whole body (weight) Sperm Epididymis	1 Apoptosis 4 Reproductive 8 Inflammatory 1 Gene stress
Amereh et al. [1] 10.1016/j.envpol.2020.114158	Polystyrene Spheres	0.025 and 0.05	1, 3, 6 and 20	Gavage Distilled water	Blood serum Sperm Testis	16 Reproductive
Li et al. [64] 10.1007/s11356-021–13,911-9	Polystyrene Spheres	0.5	0.083, 0.83, 8.3^b^	Drinking water Deionized water	Sperm Testis	3 Apoptosis 8 Reproductive 1 Inflammatory 4 Oxidative stress 1 Gene stress
Xie et al. [124] 10.1016/j.ecoenv.2019.110133	Polystyrene Spheres	5	0.43, 4.25, 43.7^b^	Drinking water Deionized water	Whole body Testis Sperm Blood serum	1 Apoptosis 4 Reproductive 3 Inflammatory 2 Energy metabolism 3 Oxidative stress 1 Gene stress 1 Body condition
***Female reproduction***						
An et al. [3] 10.1016/j.tox.2020.152665	Polystyrene Spheres	0.5	0.083, 0.83, 8.3	Drinking water Deionized water	Blood serum Ovaries	4 Apoptosis 9 Reproductive 5 Oxidative stress
Hou et al. [48] 10.1016/j.ecoenv.2021.112012	Polystyrene Spheres	0.5	0.015, 0.15, 1.5	Drinking water Deionized water	Blood serum Ovaries	2 Apoptosis 2 Reproductive 9 Inflammatory 4 Oxidative stress
Park et al. [85]^e^ 10.1016/j.toxlet.2020.01.008	Polyethylene Fragments	16.9	3.75, 15, 60	Gavage drinking water	Blood Lung Kidney Spleen Testis Stomach Seminal vesicles Ovaries Heart Thymus Epididymis Duodenum Small intestine Large Intestine Uterus Brain	4 Organ level 12 Blood biomarkers 1 Body condition 4 Reproductive 17 Immune
***Heart***						
Wei et al. [116] 10.1002/tox.23095	Polystyrene Spheres	0.5	0.087, 0.865, 8.56^c^	Drinking water Deionized water	Spleen Heart Blood serum	1 Apoptosis 3 Heart tissue 9 Inflammatory 4 Oxidative stress
Li et al. [65] 10.1016/j.envpol.2020.115025	Polystyrene Spheres	0.5	0.087, 0.865, 8.56^c^	Drinking water Deionized water	Heart Blood Serum	4 Apoptosis 10 Heart tissue 4 Oxidative stress
***Liver***						
Deng et al. [29] 10.1038/srep46687	Polystyrene Spheres	5 and 20	0.272, 2.613, 13.56	Gavage Milli-Q water	Whole body Liver	2 Liver tissue 1 Nervous system 1 Body condition 2 Energy metabolism 2 Lipid metabolism 3 Oxidative stress
***Thyroid***						
Amereh et al. [2] 10.1039/c9tx00147f	Polystyrene Spheres	0.025 and 0.050	1,3,6,10 ^c^	Gavage distilled water	Blood serum	5 Thyroid 3 Lipid metabolism 2 Gene stress
**Gut Microbiome**						
Li et al. [62] 10.1016/j.chemosphere.2019.125492	Polyethylene Spheres	10 to 150	6, 60, 600 μg ·d^−1 d^	Food Basal feed	Blood serum Colon Duodenum Spleen Feces	4 Intestinal 6 Immune 4 Inflammatory 14 Microbiome

^a^Numbers represent individual endpoints tested grouped by general effect category, which may include varying levels of biological organization from sub-cellular responses (e.g., changes in gene expression) to organismal level impacts (e.g., changes in body weight), additional details reported in Table S5

^b^Body-weight normalized exposure concentration estimated based on reported water intake rate, reported average reported body weight, and reported exposure concentration in drinking water fed to rodent in study

^c^Estimated exposure concentration based on reported concentration in drinking water and average reported body weight. Authors did not report drinking water intake rate of rodents, so an average value for this strain and life stage of rodent was used (1.7 × 10^–7^ L ·kg^−1^·d^−1^; Hou et al. [47])

^d^Body weight was not reported by authors, so body-weight normalized exposure concentrations could not be determined

^e^multi-generational effects, both male and female mice included in the study, with emphasis on effects related to female reproduction

An important observation across studies is a reliance on the use of monodisperse particles (monodisperse particles used in 21 in vivo ingestion studies; representing 72% of studies evaluated), with particle sizes across the different studies ranging from 5 nm to 80 µm in length, representing two polymers, including polystyrene (*n* = 24) and polyethylene (*n* = 5). With regards to particle size, 20 out of 29 studies reported specific information on particle size variance (Fig. S4). The majority of studies used spheres (*n* = 22), followed by fragments (*n* = 2), with 5 studies not reporting the particle’s shape. We further note that none of the studies evaluated adverse effects in relation to drinking water exposure to plastic microfibers, which are the majority of what is detected globally in environmental samples (Athey and Erdle [7]; Singh et al., [100]). As summarized in Table 2, the types of MPs tested in the 12 prioritized studies are predominately polystyrene spheres, between 5 nm and 20 μm.

The heavy reliance of reporting effects for MPs based on results obtained from a monodisperse group of particles represents a major challenge when attempting to assess the implications of the great diversity of MPs that humans are exposed to. Recently, methods have been proposed to translate toxicologically relevant characteristics of MPs used in the laboratory to those relevant for actual exposure (Kooi et al., [60]). Toxicologically relevant metrics (TRMs) for inflammation and oxidative stress have been validated for particles smaller than < 100 nm, corresponding to specific surface area and surface area, respectively (Schmid and Stoeger [98]; Tran et al., [109]). While promising and theoretically correct, further validation to which extent these translations adequately predict effects observed in mammalian test systems for different types of particles, more dedicated experimentation is advised, especially regarding applicability to particle sizes < 10 µm. Therefore, at this stage an appropriate level of caution is warranted when attempting to interpret and predict human health implications in a risk assessment context. While not defined as a ‘red criteria’ for pragmatic purposes, it is important to note that only 43% of all studies reviewed attempted to address the environmental relevance of the particles tested. Studies using environmentally realistic MP mixtures are encouraged, however monodisperse studies still have merit in elucidating differences in toxicodynamics and toxicokinetics between particle types.

The observation that a number of studies fail to address criteria not used for prioritization purposes is important to highlight. While the 12 studies listed in Table 2 satisfactorily addressed the selected set of quality criteria, there are several aspects that have been poorly addressed and which also represent additional issues considered as part of the expert evaluation process. In addition to the challenge of extrapolating from monodisperse to an environmentally relevant exposure of MPs, for instance, there are also concerns related to the lack of information presented in each of the 12 studies regarding the removal of any chemical contaminants that may be associated with the test particles (e.g., using dialysis, gas purging, extraction, etc.) and which may have influenced test results. Across all studies reviewed, only 10% addressed the presence of chemical contaminants through cleaning of particles before administration (Fig. 2), with none of the 12 studies listed in Table 2 satisfactorily reporting this information. Additionally, all studies rely on nominal concentrations used in the reporting of dose–response relationships, however, concerns are raised regarding the particle stability (i.e. aggregation/agglomeration, etc.) in the test media. Furthermore, few studies provided analytical verification and quantification of the actual tissue concentrations of MPs. A more detailed discussion related to the relative importance of each of the quality criteria is presented in Gouin et al. [42], including the potential influence that not addressing each of the criteria may represent with respect to data interpretation.

### Tier 2 expert evaluation

The tiered-approach used to support workshop objectives is illustrative of a constructive process aimed at helping to inform workshop discussions associated with the various factors that might influence the interpretation of data reported from studies for use in deriving a health-based screening level. Fundamentally, two main challenges can be identified, and which are characterized by a requirement to evaluate the quality and reliability of studies confidently and transparently. The first challenge, addressed by the Tier 1 screening and prioritization exercise described in Gouin et al. [42], relates to the need to assess whether a study has adequately addressed a prioritized subset of fundamental QA/QC criteria. The second is represented by a need to more thoroughly evaluate the strengths and limitations of the various physiological endpoints measured and reported for prioritized studies. Table 2 illustrates that a number of different toxicological endpoints have been addressed, including several studies reporting on the mammalian reproductive toxicity of MPs in drinking water, as well as adverse effects on the function of various organs and physiological systems, including the potential impact that oral ingestion of MPs may have on the gut microbiome. Given the specialized technical expertise required to fully evaluate the various endpoints reported, several external experts, primarily with expertise in the area of reproductive physiology, were recruited to provide qualitative evaluations of each of the prioritized studies. The results of the external expert review are documented in the Supplementary Information.

The opinions and judgements documented by the external experts provide specialized insight into the challenges of conducting and interpreting results reported in each of the 12 studies. The insight gained from the qualitative evaluations documented in the SI, coupled with the Tier 1 QA/QC screening results, represent the primary sources of information influencing workshop discussions and the determination of which endpoints should be considered for use in deriving a human-health screening level value. Table 2 reports a variety of physiological endpoints across the 12 studies that were considered with respect to their relevance and reliability by both external experts and workshop participants, and which relate to a range of both organism- and cellular-level toxicological endpoints. Observed adverse effects for all physiological endpoints and studies were in response to an oral sub-chronic exposure to a monodisperse group of MPs (predominantly polystyrene spheres) via gavage or drinking water.

Of the endpoints considered, several were identified as relevant for use in deriving a screening level. In general, a consensus between the external experts and workshop participants resulted in an emphasis towards organismal-level endpoints, as well as various sub-organismal biomarkers with a strong connection to a human relevant endpoint of potential concern. These include several related to toxicological effects on male reproduction (i.e. testosterone concentration, testicular capsule diameter, sperm cell concentration, sperm count, deformity, viability, motility and maturity, sperm DNA damage, seminiferous tubules diameter, luteinizing hormone concentration, seminiferous epithelium height, and follicle stimulating hormone), female reproduction (i.e. Anti-Müllerian hormone (AMH) concentration), liver (i.e. liver tissue inflammation) and on body condition (i.e., body weight).

Since seven of the 12 prioritized studies report adverse effects on both male and female reproductive systems in various mammalian models, the majority of the discussion presented below is directed towards summarizing insight gained through the Tier 2 expert evaluation for those studies. The important factors influencing the relative weight given towards considering the mammalian reproductive studies in the context of workshop discussions is based on two main factors. First, is the relatively high number of studies reporting reproductive effects, which may imply a weight-of-evidence with respect to a causal relationship. Secondly, since the relevance of the endpoints reported with respect to human health represent a potential source of concern to the general public, it is thus prudent to gain a good understanding of the relative reliability of the studies through an evaluation of their strengths and weaknesses. The qualitative evaluations obtained from Tier 2 experts who have expertise in the field of mammalian reproductive toxicity thus represents an important factor towards an expression of confidence in the data by workshop participants, which subsequently influences how the information gained through this process might be used within a decision-making framework.

#### Mammalian reproductive effects

Four of the studies passing red criteria (Table 2) report observations obtained from performing male reproductive toxicity studies. The adverse effects reported include a reduction in the number and proportion of viable sperm, increased sperm deformities, as well as apoptosis of sperm cells accompanied by a dose-related expression of cytokines, which can serve as biomarkers of underlying inflammation (Table S6) (Hou et al., [47]; Xie et al., [124]; Li et al., [64]; Amereh et al., [1]). Accumulation of polystyrene spheres of varying sizes (i.e., 0.5 μm, 4 μm and 10 μm) is reported in the testis by Jin et al., [54] at 24 h following an exposure via daily 28d repeat-dose oral gavage of 1 mg · d^−1^, resulting in an observation of reduced sperm quality and testosterone levels at levels that were not significantly different between particle sizes. Jin et al., [54] could not be used for dose–response assessment due to having only one exposure concentration and is therefore not included in Table 2. The observed adverse effects from the various mammalian studies on male reproduction (Hou et al., [47]; Xie et al., [124]; Li et al., [64]; Amereh et al., [1]; Jin et al., [54]), such as an increase in sperm abnormalities and decreased sperm motility and concentrations is further suggested to cause an impairment of the blood-testis barrier (Li et al., [63]). Consequently, a general observation across the four male mammalian reproductive toxicity studies passing red criteria (Table 2) and Jin et al., [54]), is that the combined observations related to a decrease in testosterone levels, an increase in inflammation markers, and a decrease in proteins involved in oxidative stress defence, suggests that there is either a direct effect of the polystyrene spheres tested on testicular function or that the observations reflect secondary effects that can occur due to inflammation (Azenabor et al., [8]). The ability to reliably evaluate if the effects on testicular function represent a direct response to exposure to the test materials, however, cannot be confidently determined, since none of the studies performed a proper analysis of testicular histology.

Furthermore, the observed effects on sperm viability and morphology may also be due to defects occurring in the epididymis itself. Since it is understood that as sperm mature and gradually acquire motility as they travel from the caput (top section) to the cauda (bottom section) epididymis, their motility also changes from irregular (abnormal) motility to normal sperm motility. Thus, in order to obtain a reliable impression of sperm motility, sperm should be collected from the cauda epididymis (Cornwall [24]). Of all the studies that report a decreased sperm motility, only one collected sperm from the cauda epididymis (Jin et al., [54]), which cannot be used to support the objective of the workshop to derive a human health screening level due to using a single exposure concentration. The observed response in the study of Jin et al., [54], however, may provide support to the observations reported in prioritized studies listed in Table 2, where similar responses following exposure to polystyrene spheres were observed. Nevertheless, in the absence of greater mechanistic understanding caution is warranted not to overinterpret results, with a recommendation for future studies to ensure collection and interpretation of results follow guidance applicable to mammalian reproductive effects studies (see OECD Test Guideline numbers 421 [79] and 422 [78]).

Additional examples of concern associated with each of the studies raised through the Tier 2 expert evaluation are detailed in the SI, which include an observation that while testis weight was reported to decrease by (Hou et al., [48] and Li et al., [64]), the application of an inappropriate method used to interpret the effects on testis weight is potentially problematic. For instance, Hou et al., [47] report the effect on testis weight as a coefficient relative to whole organism body weight, which is an inappropriate analysis due to the lack of significant effects observed on the body weight of test animals in that study.

The importance of confirming particle exposure and translocation into target tissues can be illustrated by one of the studies included in Table 2 (Park et al., [85]), which reports an absence of effects on reproductive outcomes, in contrast to other studies evaluated (i.e. Hou et al., [47]; Xie et al., [124]; Li et al., [64]; Amereh et al., [1]; Jin et al., [54]). Park et al., [85]) assessed mating and fertility outcomes of five pairs of mice exposed for 90 days to polyethylene fragments, modified to contain acid and hydroxy groups, with a measured size of 16.9 ± 1.9 µm following oral gavage, where no statistically significant effects on growth, viability, fertility, fecundity or viability in the parent or offspring generations following a re-evaluation of the underlying data (supplemental information) are reported. The absence of an observed effect, however, may be due to a lack of translocation associated with the relatively large size particles tested, making it difficult to directly compare and extrapolate potential effects on mammalian reproduction between studies using polystyrene spheres < 5 μm to polyethylene fragments > 10 μm. This hypothesis (lack of translocation due to size) cannot be confirmed due to the absence of confirmed particle translocation in tissue, and illustrates a recurring issue across the studies which prevents determination of causal relationships between adverse effects and the relative potential for tested particles to translocate and accumulate within organs of interest.

For instance, some of the earliest studies on MPs conducted in rodents are described by (Jani et al. [50], [51]), where female Sprague Dawley rats were exposed to polystyrene spheres of various sizes (0.05—3 µm across the two studies), and which provided an early indication for the potential of variable but dispersed internalization and translocation of polystyrene spheres following oral gavage. The physiological uptake of non-ionized MPs is suggested to occur via Peyer’s patches, which are groups of lymphoid follicles (similar to lymph nodes) distributed throughout the mucosa of the ileum, with smaller quantities also found in the small intestine (Wright and Kelly [122]). The moderate uptake of uncharged polystyrene spheres (100 nm – 1 μm) has been observed and reported in the colon and liver, with the uptake of negatively charged particles observed to be less than that of uncharged particles (Jani et al., [50]). In later studies, a particle-size dependency has been reported for polystyrene spheres (50 nm – 3 μm) extracted from the tissues of the stomach, small intestine, and colon (Jani et al., [51]). Specifically, Jani et al., [51] reports a decrease in the translocation of polystyrene spheres with increasing particle size, with a cumulative translocation falling from 6% for 50 nm particles to around 1% for 1 μm and zero for 3 μm.

More recently, a number of studies have reported the uptake of polystyrene spheres (2–20 μm) in the gut, liver, and kidney tissues of mice, with a number of adverse effects being observed and reported, including oxidative stress, altered metabolic profile and lipid metabolism, as well as chronic inflammation (Deng et al., [29], Jin et al., [55], Li et al., [64], Shengchen et al. [99], Wang et al., [114], Xu et al. [125], Zheng et al., [127]). Generally, a particle-size dependence influencing the uptake and presence of polystyrene spheres in various tissues resulting in a variety of adverse effects has been reported, with smaller particles (< 1 µm) understood to have a higher likelihood for translocation (e.g., Jani et al., [50], [51]). Nevertheless, some studies have reported the uptake of polystyrene spheres between 5–20 μm (e.g., Deng et al. [29], Zheng et al. [127]), although the uptake of these larger particles represents a source of debate (Stock et al., [104]), with Stock et al. [105] observing differences in uptake between MPs (1–4 μm) of varying polymeric composition reported from an in vitro Caco-2 based transwell model. Consequently, there appears to be a variety of factors to consider when attempting to interpret the physiological effects that exposure to a monodisperse group of particles represents, such as the influence of particle size, surface charge and polymeric composition on the uptake and systemic distribution in relation to various organ-specific effects.

While the majority of mammalian reproductive studies report results pertaining to effects on males, two of the studies listed in Table 2 specifically evaluated the adverse effects of 500 nm polystyrene spheres on mammalian female reproduction (An et al. [3], Hou et al. [48]), with a particular emphasis on ovarian histology, serum AMH concentrations and markers of oxidative stress, inflammation and apoptosis in ovaries. As a general observation, we note that both studies are strikingly similar in many aspects, including study design and results. The consistency between the two studies can thus potentially result in a heightened perception of a causal relationship between exposure to the polystyrene spheres tested and female reproductive effects, particularly since results are supported by more than one study reporting similar observations. It should be noted, however, that the studies have been conducted by individuals and institutions common between the two publications and that while Hou et al. [48] report exposure doses for rats based on a body weight adjusted dose (i.e., 0.015–1.5 mg·kg^−1^·d^−1^), An et al. [3] do not (0.015–1.5 mg/d). Thus, while two separate studies report consistent effects for serum AMH levels, ovarian follicle counts, and markers of oxidative stress, inflammation and apoptosis, the results do not necessarily support replication between different groups and instead may be considered an intralaboratory comparison.

When considering specific aspects of the studies, we emphasize the importance of performing quantitative analysis of ovarian histology following a systematic approach, for example the OECD TG443: Extended one-generation reproductive toxicity study guideline (OECD [80]), which we note was not performed in either of the studies of (An et al. [3] or Hou et al. [48]). Consequently, the lack of a systematic approach in relation to the ovarian histology represents an underlying concern raised by the Tier 2 experts (supplemental information), and which can result in a reduced reliability of the data. Nevertheless, when assessing human female reproductive effects based on animal data, the most relevant sub-organismal biomarker associated with fecundity potential of women is understood to be represented by AMH. AMH is a growth factor secreted by early growing follicles and is an established biomarker of ovarian reserve in women, and therefore widely used in fertility assessment of women during infertility treatments (Broer et al., [14]). Because AMH is typically temporally stable, it is considered to be a more reliable indicator of female fecundity than other biomarkers such as steroid hormones, gonadotropins, and antral follicle counts which fluctuate cyclically during the ovarian cycle (Broer et al., [14]). While serum AMH is used in reproductive medicine to evaluate the likelihood of success in retrieving a sufficient number of oocytes in ovarian hyperstimulation during infertility treatments, uncertainties remain about its implications for female fertility potential in the general population (Broer et al., [14]). To reliably assess the potential for MPs to cause infertility in females, antral follicles, and corpora lutea should also be quantified as markers of folliculogenesis and ovulation in humans. However, due to the heterogeneity of follicle size distributions in ovaries, a systematic approach must be applied towards the counting of follicles and corpora lutea. Neither of the female reproductive papers (An et al., [3]; Hou et al., [48]) used a systematic approach to quantify follicles, nor did they specify what types of follicles were counted. Consequently, while AMH represents a highly relevant endpoint, uncertainties are expressed by the Tier 2 experts regarding its reliability for use in the derivation of a human-health based screening level.

#### Mammalian non-reproductive effects

Several in vivo mammalian ingestion-based studies reported effects on heart, liver, thyroid, and the gut microbiome (Tables 2 and S6), with many studies unable to be used for screening level derivation due to their lack of at least three exposure concentrations. For instance, Li et al., [63], report adverse effects of 5 μm polystyrene spheres in drinking water in the livers of male mice exposed to 20 mg·kg bw^−1^·d^−1^ for 30 days. The elevated exposure to the 5 μm particles resulted in inflammation and apoptosis (Li et al., [63]), observations that are consistent with the induction of inflammation and altered liver metabolism in mice with acute colitis reported by Zheng et al. [127] (Table S6). Observations of metabolic disorders involving dysbiosis of the gut microbiota have also been reported, accompanied by gut barrier dysfunction and increased intestinal permeability in mice exposed to polystyrene spheres between 500 nm and 50 μm via drinking water (Lu et al. [68], Luo et al. [69], Zheng et al. [127]; see Table S6 for additional details). In the study of Li et al. [62], listed in Table 2, however, an increase in the diversity of gut microbiota is reported, although biomarkers related to an inflammatory response, such as interleukin-1α was reported to increase in the treatment group, relative to the control group.

### Potential effect mechanisms

Biomarkers suggestive of potential toxicity mechanisms in evaluated studies include reactive oxygen species generation, DNA damage, inflammation, cytotoxicity, and alterations to lipid and energy metabolism. When considered from the perspective of aligning biomarker responses to a potentially relevant adverse outcome pathway (AOP), there may be an opportunity to gain some additional mechanistic insight with respect to the adverse effects that exposure to MPs might initiate. A recently published putative AOP with an emphasis on assessing toxicological mechanisms of particle toxicity in mammals, for instance, and which among other species included adverse effects on mice and zebrafish, both commonly used for the study of human health implications (*Mus musculus* and *Danio rerio*), suggested the use of several biomarkers in identifying potential key events, and which may play a role in inducing adverse responses following MP exposure (Jeong and Choi [53]). These include oxidative stress (KE1392), reactive oxygen species generation (KE1278), increased inflammation (KE149), and lipid peroxidation (KE1511) (Jeong and Choi [53]). Although more evidence is needed to better understand the relationship between exposure to MPs and the triggering of various relevant biomarkers used within an AOP-context, responses reported in the studies listed in Tables 2 and S7, as well as those from various in vitro based studies, may align with existing AOPs. For instance, AOP 173, for which the adverse outcome is lung fibrosis following an inhalation exposure pathway, has a proposed molecular initiating event (MIE) cellular membrane interaction with nanomaterials (Halappanavar et al., [44]), and is one of several AOPs that have been tentatively linked to microplastics (Halappanavar and Mallach [43]).

While not conclusive, the adverse outcomes and biomarkers associated with MP exposure may also have some similarity with effects reported for nanoparticles, such as titanium dioxide (TiO2), whereby key events include the induction of oxidative stress and inflammation in the liver and intestine (Brand et al. [11]). Although an appropriate level of caution is warranted not to overinterpret results in the context of a specific MIE, particularly given current knowledge gaps and various concerns related to the relative quality and reliability of studies (documented in the supplemental information), we do suggest that the use of biomarkers can play an important role towards an improved mechanistic understanding of adverse effects, with appropriate consideration of dose and time response behavior. Specifically, we recommend future studies consider the implications of MP-induced toxicity in the context of inflammation and oxidative stress through an AOP-based perspective (Ankley et al. [5]). For example, Villeneuve et al. [113] proposed that researchers should consider factors such as damage-associated molecular patterns, which can eventually lead to cell damage and tissue dysfunction, when elucidating the underlying causes of oxidative damage and inflammation.

In terms of specific molecular pathways highlighted in the in vitro studies included as part of this activity, and which are supportive of responses reported in the mammalian in vivo studies, both Hou et al. [47] and Wei et al. [116] report a dose-dependent alteration of Nf-κb, a transcription factor known to induce the expression of cytokines and as an initiator of general inflammatory responses (Liu et al. [67]). These observations are consistent with results reporting the effects of MPs on gastric cells and the induction of pro-inflammatory cytokines such as IL-6 (e.g., Forte et al. [40], Hwang et al. [49]), biomarkers that have been shown to result in downstream Nf-κb activation (Brasier et al. [13]). Several other in vitro studies provide additional lines-of-evidence linking exposure to MPs with a pattern of overall inflammation and reactive oxygen species production, similar to observations in live animal models (Wang et al. [114]; Choi et al. [21], Dong et al. [32], Wu et al. [123]; Efeoglu et al. [34]). Although we emphasize the potential importance of these observations towards an improved mechanistic understanding of the adverse effects associated with MPs, we also note the need for additional research towards strengthening various QA/QC components of study design and implementation to enable better comparison and extrapolation of results between studies using different cell types and target organs and with a variety of different types of MPs, prior to reliably aligning to any MIEs.

#### Human-health guidance level recommendation

**Fig. 3:**
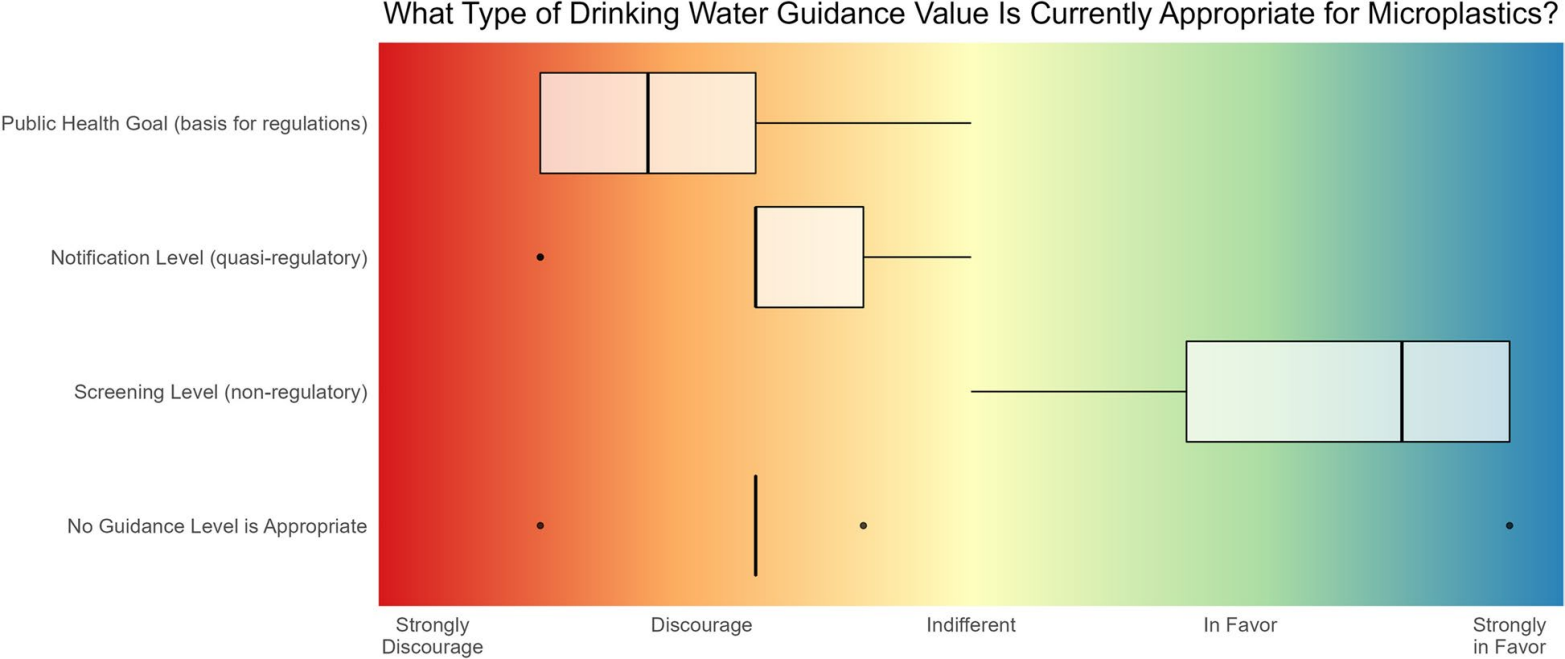
Results from the polling of workshop participants in relation to an appropriate health-based threshold level that the California State Water Resources Control Board should adopt for MPs in drinking water. Results presented are based on the experts’ qualitative judgement of the evidence of health effects obtained from the studies evaluated through the Tiered approach presented in this study. The box and whiskers plot represents the summary of votes (*n* = 9), with the upper and lower boundaries of the box indicating the 75th and 25th percentiles, respectively. The line within the box marks the median, error bars indicate the 90th and 10th percentiles, and individual points represent statistical outliers

Following the insight gained from the combined output of the tiered-approach applied in this study regarding the quality and reliability of MP effects data, workshop participants were polled regarding their relative confidence with respect to the use of data in application against three main thresholds used for drinking water management as defined by the State of California. The three main thresholds consist of one non-regulatory Screening Level, a quasi-regulatory level – represented as a Notification Level, and a level used as the basis for informing regulatory actions, i.e., a Public Health Goal. The results of the polling exercise are summarized in Fig. 3, where it can be seen that the overall consensus was that the data were markedly insufficient for either of the two levels on which advisory and regulatory actions may be based (i.e., Notification Levels and Public Health Goals, respectively), but were considered adequate for supporting the derivation of a non-regulatory screening level, from which an appropriate volume of drinking water can be estimated towards helping to guide the monitoring of MPs in California’s drinking water supply.

The output of the expert discussions and judgements, summarized as supporting the derivation of a non-regulatory human-health screening level, demonstrate the need for future studies to strengthen the application and implementation of test methods sufficient to produce relevant and reliable data appropriate for informing the potential implications that exposure to MPs represent to human health. Consequently, in addition to estimating a non-regulatory human health screening level, insight gained from this exercise is also used towards the prioritization of a number of research recommendations (Thornton Hampton L, Bouwmeester H, Brander S, Coffin S, Cole M, Hermabessiere L, et al: Research Recommendations to Better Understand the Potential Health Impacts of Microplastics to Humans and Aquatic Ecosystems, In Press).

### Screening level derivation

In order to support the derivation of a screening level value, Tier 2 experts and workshop participants were asked to consider which of the endpoints reported in the studies listed in Table 2 should be used in its estimation. As noted above, endpoints with a human health relevance were identified as representing the most appropriate candidates. In order to quantify the relative uncertainty in relation to the data for selected endpoints, the respective upper and lower 95% confidence intervals were modelled using the US EPA BMDS, and which are summarized in Table 3.

**Table 3:** Point of departure concentrations for selected endpoints used as input for deriving a human-health screening level. All studies report adverse effects in relation to polystyrene spheres of varying sizes, as summarized in Table 2

**Study**	**Endpoint**	**Metric used for POD**^**a**^	**NOAEL**^**b**^ (mg /kg-day)	**LOAEL**^**b**^ (mg /kg-day)	**Best-fit Model**^**c**^	**BMDS Recommendation**^**d**^	**Scaled residual for dose group near BMD** (unitless)	**Scaled residual for control dose group** (unitless)	**BMDL** (mg /kg-day)	**BMD** (mg /kg-day)	**BMDU** (mg /kg-day)
Amereh et al. [1]	Luteinizing hormone concentration	LOAEL/10	NA	1	Hill	Viable	0.17	0.17	NA	NA	NA
Amereh et al. [1]	Follicle stimulating hormone concentration	LOAEL/10	NA	1	Exponential 3	Questionable	7 × 10^–5^	3.99	NA	NA	NA
Amereh et al. [1]	Testosterone concentration	BMDL	NA	1	Exponential 4	Viable	0.23	0.23	0.26	0.46	Infinity
Amereh et al. [1]	Sperm count	BMDL	NA	1	Hill	Viable	0.30	0.30	0.27	0.41	0.7
Amereh et al. [1]	Sperm motility	BMDL	1	3	Exponential 4	Viable	-0.45	0.12	0.54	0.79	1.2
Amereh et al. [1]	Sperm DNA damage	BMDL	1	3	Polynomial Degree 3	Viable	-1.02	0.49	0.67	0.82	1.2
Amereh et al. [1]	Sperm maturity	BMDL	1	3	Exponential 3	Viable	0.85	-0.20	0.78	0.97	1.6
Amereh et al. [1]	Sperm viability	BMDL	NA	1	Polynomial Degree 3	Viable	-0.85	1.01	0.85	1.0	1.4
Amereh et al. [1]	Sperm Deformity	BMDL	1	3	Polynomial Degree 3	Viable	0.89	-0.51	1.0	1.2	1.8
An et al. [3]	Anti-Mullerian hormone concentration	BMDL	0.015	0.15	Exponential 4	Viable	0.090	-0.077	0.14	0.20	0.32
Deng et al. [29]	Liver Condition Index	BMDL	2.6	13	Exponential 4	Viable	-0.18	-0.57	0.68	2.5	39
Deng et al. [29]	Liver Condition Index	BMDL	2.6	13	Polynomial Degree 3	Viable	-0.01	0.71	4.98	10	13
Hou et al. [47]	Testis Weight	LOAEL/10	NA	0.017	Exponential 3	Questionable	0.08	2.46	NA	NA	NA
Hou et al. [47]	Sperm deformity	NOAEL	0.17	1.7	Hill	Questionable	7 × 10^–6^	0.74	NA	NA	NA
Hou et al. [48]	Anti-Mullerian hormone concentration	BMDL	0.083	0.83	Exponential 5	Viable	-0.022	0.019	0.025	0.034	0.14
Li et al. [64]	Sperm deformity	NOAEL	0.83	8.3	Linear	Questionable	1.46	-1.04	NA	NA	NA
Xie et al. [124]	Body weight	LOAEL/10	NA	0.43	Exponential 2	Questionable	-0.02	0.42	NA	NA	NA
Xie et al. [124]	Sperm count	LOAEL/10	NA	0.43	Exponential 4	Questionable	0.77	1.79	NA	NA	NA
Xie et al. [124]	Sperm deformity	LOAEL/10	NA	0.43	Exponential 4	Questionable	-0.28	-1.37	NA	NA	NA
Xie et al. [124]	Testosterone concentration	LOAEL/10	NA	0.43	Polynomial Degree 3	Questionable	0.13	2.07	NA	NA	NA

*NA* Not Available

^a^When viable BMD models were recommended by BMDS software, BMDs were used as the POD. If not, the NOAEL was used. When NOAELs were unavailable, the LOAEL was used with an UF of 10

^b^Studies did not always explicitly refer to exposure concentrations as LOAELs and NOAELs and were often inferred from text and figures based on statistical relationships relative to control

^c^ Models selected for use met all of the default threshold criteria set by BMDS software, such as constant variance, goodness of fit, ratio of BMD/BMDL, absolute residual of controls and exposure, etc. and had the lowest Akaike’s Information Criteria of models tested (US EPA [36]). All models are based on a normal coefficient of variance

^d^EPA’s BMDS software provides a recommendation for the viability of the best-fit model. When models were recommended as ‘viable’, BMDs were used. Otherwise, NOAELs or LOAELs were used as point-of-departures

**Fig. 4:**
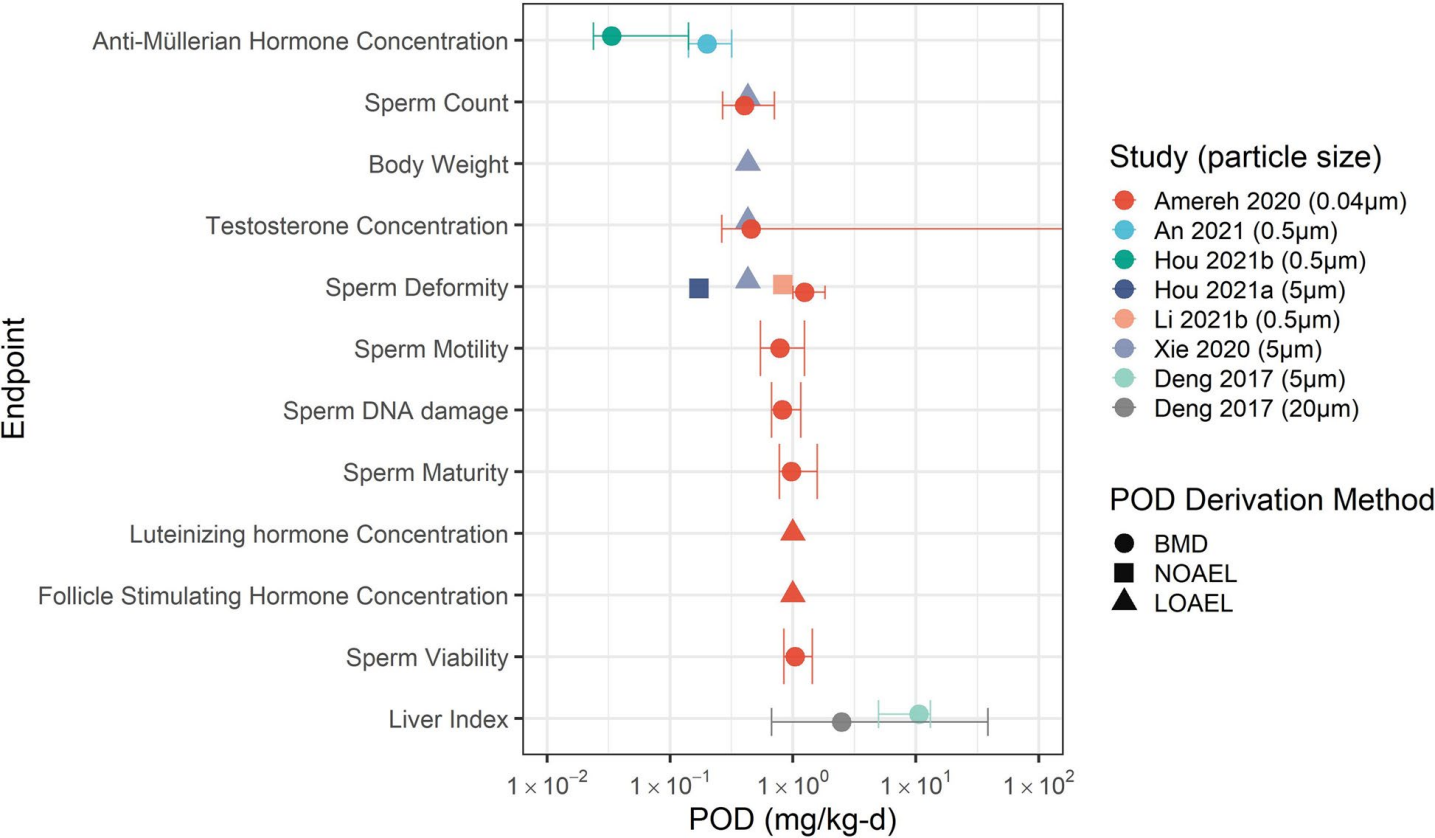
Summary of BMD modelling output results for endpoints deemed reliable by outside experts. Points represent BMDs, and error bars represent 95% confidence intervals predicted by US EPA BMDS software

For several endpoints, application of the US EPA BMDS failed to converge on a reliable model for quantifying a benchmark dose, largely due to non-monotonic dose–response relationships (e.g., sperm deformity reported in Hou et al. [47]), of which are common with endocrine/reproductive-related endpoints (Vandenberg et al. [111]). The results reported in Table 3 are further illustrated in Fig. 4, which graphically summarizes the output obtained from the BMD model providing the best-fit, and which further illustrates the range of variability between the BMDU and BMDL estimates as well as the relative difference in sensitivity in BMD, such as between the response reported for reducing AMH concentration and responses reported in relation to the liver condition index. Additional discussion of the application of the BMDS model, including a comparison between the BMDS and RIVM PROAST tools, used here to enable a statistical assessment regarding each of the endpoints for use in the screening level derivation, is included in the Supplementary Information.

For endpoints that could not be reliably modelled using the US EPA BMDS, NOAELs were used as PODs, or if unavailable, the LOAEL was divided by a factor of 10, representing an uncertainty adjustment factor extrapolating from the LOAEL to the NOAEL, following guidance by OEHHA (OEHHA [81]). It is important to note that since it is currently not possible to enable a read-across between particles of varying composition, size and shape, the PODs presented in Table 3 and Fig. 4 are grouped by the exact particle sizes of polystyrene spheres that were used in each of the studies, as listed in Table 2. Caution is thus warranted not to interpret results as representing a POD for the heterogeneous mixture of MPs, typical of an environmental exposure. This is an important point to emphasize, since the presence of polystyrene in drinking water represents a negligible fraction of the types of MPs that have been identified in drinking water, whereby particles consisting of polyethylene, polypropylene and polyethylene terephthalate are typically reported (WHO, [120]). From Fig. 4 it can be seen that several of the endpoints appear to have a POD that centers around 1 mg·kg^−1^·d^−1^, with the PODs for reduction of AMH concentration from Hou et al. [48] and An et al., [3] being approximately one to two orders of magnitude lower than that of the responses in relation to liver condition, which is an order of magnitude higher. For derivation of the screening level, the lowest POD is used, which is the BMDL from Hou et al., [48] for reduced AMH concentration (0.024 mg·kg^−1^·d^−1^), however a maximum POD is also considered for the purposes of assessing the sensitivity of the POD, which is the BMDL for reduced liver condition index from Deng et al. [29], i.e., 4.98 mg·kg^−1^·d^−1^.

#### Screening level sensitivity and uncertainty analysis

Acknowledging the various sources of uncertainty that surround the underlying hazard data and the estimates of the PODs, while also attempting to support a conservative approach in the derivation of a non-regulatory screening level as defined by Eq. 1, a sensitivity and uncertainty analysis has been performed. In the instance of the sensitivity analysis, the influence of the relative magnitude between the upper and lower 95% confidence interval for each of the endpoints is considered, as is the impact of assumptions related to the default values used for the RSC and the DWI. Table 4 summarizes the results of the sensitivity and uncertainty analysis, with specific details presented in the SI.

**Table 4:** Sensitivity analysis of screening level (μg·L^−1^) for the most sensitive POD identified as a reduction in AMH concentration as shown in Table 3 and Fig. 4

Reference dose^a^ = 0.024 mg·kg^−1^·d^−1^ (BMDL)			
	RSC = 0.00001	RSC = 0.20	RSC = 0.50
DWI = 0.022 L·kg^−1^·d^−1^	0.011	230	540
DWI = 0.053 L·kg^−1^·d^−1^	0.0045	90	240
DWI = 0.135 L·kg^−1^·d^−1^	0.0018	36	89
Reference dose^a^ = 0.034 mg·kg^−1^·d^−1^ (BMD)			
	RSC = 0.00001	RSC = 0.20	RSC = 0.50
DWI = 0.022 L·kg^−1^·d^−1^	0.015	310	760
DWI = 0.053 L·kg^−1^·d^−1^	0.0063	130	320
DWI = 0.135 L·kg^−1^·d^−1^	0.0025	50	120
Reference dose^a^ 0.141 mg·kg^−1^·d^−1^ (BMDU)			
	RSC = 0.00001	RSC = 0.20	RSC = 0.50
DWI = 0.022 L·kg^−1^·d^−1^	0.064	1300	3200
DWI = 0.053 L·kg^−1^·d^−1^	0.027	530	1300
DWI = 0.135 L·kg^−1^·d^−1^	0.010	210	520

^a^A composite uncertainty factor of 1 was used for derivation of these reference doses. The default composite UF that OEHHA would use in this case would be 300

While BMDLs were used as default values for calculating screening levels (when available), uncertainties introduced to the screening level due to the use of BMD modelling software is assessed here by considering the BMD and associated 95% confidence intervals (i.e., BMDL and BMDU). The relative sensitivity of the variability associated with BMDs varies by each POD, ranging from an absolute percentage difference of 9% to 315%, with an average variability of 54% (Fig. 4). The BMD for the lowest POD (AMH; Hou et al. [48]), for instance, has consistent variability between the upper and lower 95% confidence intervals (-29% to 311%) relative to all other BMDs modelled based on the lower and upper 95^th^ percentiles, respectively), which is due to the variability in the relative uncertainties estimated using BMDS. Depending on which endpoint might be used as a POD, the relative influence of its uncertainty greatly influences the impact on the sensitivity with respect to deriving a screening level value. For instance, the variability in the screening level for the most sensitive POD, identified as a reduction in AMH concentration, results in a difference of between 90 and 540 μg · L^1^ in the estimated screening level value, when assuming default values for the DWI and the RSC (Table 4), or about a factor of 5 difference. The relative sensitivity of the variability associated with the POD on the derivation of a screening level value when using AMH as the most sensitive endpoint is not surprising, which when coupled with the various concerns raised above regarding the studies from which the data were obtained, implies the need to acquire higher quality effects data aimed at reducing the relative level of uncertainty that might propagate through calculations, such as characterized by Eq. 1.

As noted in the methods section, a default value of 20% has been defined for the RSC, which is understood to represent a conservative approach in instances where exposure data in drinking water might be limited – such as in the case of MPs in California’s drinking water (OEHHA [83]). Thus, recognizing that depending on the ability to quantify the contribution that exposure to MPs from drinking water might represent, ranging from a dominant source to negligible, intuitively represents an additional data need. While the variability in the POD represents an approximate difference in the screening level derivation of a factor of 5, the sensitivity of the assumption related to the RSC is observed to vary by several orders of magnitude, ranging from between 0.0047 to 240 μg·L^−1^ when assuming a default POD (i.e., BMDL) of 0.024 mg·kg^−1^·d^−1^ and the default value for DWI (Table 4). It is therefore prudent to ensure future research activities support the characterization and quantification of MPs in California’s drinking water, which when coupled with robust hazard data will provide decision makers with a higher level of confidence in defining an appropriate level of regulatory action. Finally, we note that the relative sensitivity regarding the assumption of DWI (-63% to 146%) is observed to be lower than those identified for either the POD (-26% to 315%) or the RSC (-99.995% to 147%), consequently the relative sensitivity regarding the DWI assumption represents a relatively minor source of uncertainty in the screening level derivation.

The insight gained from the sensitivity and uncertainty analysis, particularly with respect to the influence that strengthening understanding regarding the relative source contribution of MPs in California’s drinking water represents, suggests the need to support analytical monitoring efforts. Ideally the ability for monitoring activity to reliably detect and report MPs in drinking water should be aligned with capability towards analyzing the concentration of particles that might accompany the most sensitive human-health relevant endpoint, which in this instance is the observed reduction of AMH concentrations in female rats (Hou et al. [48]). As noted above, the results reported by Hou et al. [48] should be interpreted with caution, given various concerns related to both the QA/QC screening evaluation and a lack of a systematic approach used in conducting the study according to standard methods. For the purposes of this activity, however, which are intended to support the development of guidance regarding the collection of drinking water samples, we consider a screening level derived using data reported by Hou et al. [48] to be consistent with a conservative estimate towards assessing the volume of water required to be concentrated to enable detection above a quantitation limit.

Given that the polystyrene particles used by Hou et al. [48] were 0.5 μm spheres, it is possible to estimate the number of particles associated with the nominal concentrations used in the test system based on the density of polystyrene using in laboratory experiments (1.05 g · cm^−3^; Cospheric [25]) and the equation for the volume of a sphere (V = 4/3 · π · r^3^), which results in a particle-based concentration for the screening level of approximately 2.3·10^8^ 0.5 μm polystyrene spherical particles· L^−1^ (95% CI based on BMD: 1.7·10^8^ to 9.7·10^8^ particles· L^−1^). When sensitivities with regards to RSC and DWI are considered, the screening level varies by nearly six orders of magnitude: 4.5·10^3^ to 1.4·10^9^ polystyrene particles· L^−1^.

Finally, considering that in many instances a reference dose might be estimated when a POD is used in supporting risk assessment (whereby a composite UF value of 300 might be used based on inter-species variability, intra-species variability, and database deficiencies) a non-regulatory screening level value based on default DWI and RSC parameters (i.e., 0.053 L·kg^−1^·d^−1^ and 0.20, respectively), and derived using the BMDL for AMH reduction (i.e., 0.024 mg · kg^−1^ · d^−1^), would equate to 0.3 µg · L^−1^ or 5.7·10^5^ particles· L^−1^ for 0.5 μm polystyrene spheres using Eq. 1. This value thus represents the most conservative estimate derived from the mammalian effects data considered in this study towards characterizing a potential human-health effect, which we suggest should only be used for helping to guide monitoring activities.

We emphasize that due to the various limitations identified above, that the non-regulatory screening level values reported here should not be used to imply the potential for human-health effects of MPs at these concentrations. This is important to note, since workshop participants identified three factors that led to an overall lack of confidence in the effects data for use in deriving a regulatory threshold value:

1. **Lack of established mechanism of toxicity** – Workshop participants noted that due to poor characterization of the exposure material, specifically in relation to the potential role that a chemical contaminant may have played towards influencing results, it is not possible to provide a robust determination of causality, which is further limited due to concerns raised regarding lack of confidence in the dosage at which effects occur.
2. **Particokinetics** – Studies did not report on the potential agglomeration of particles or consider the interactions between the properties of particles and the intestinal lumen, which might influence on bioavailability and aberrant dose–response curves (Dhawan et al. [31]), coupled with limited information reporting an overall mass balance aimed at quantifying the dose of particles reaching the target organ.
3. **Inability to extrapolate between effects reported for monodisperse particles and environmentally relevant MPs** – It is noted that the majority of studies tested polystyrene spheres, which have been reported at relatively low abundances (~ 10%) in surface freshwaters relative to other polymer types, with spherical plastic particles summarized by Burns and Boxall [16] as representing 6.5% of the types of particles detected in the environment. Although read-across methods that would enable a reliable extrapolation of polystyrene spheres to the heterogeneous mixture of MPs are available (see Koelmans et al. [58]; Kooi et al. [60]), caution is warranted not to use such data within a regulatory context until TRMs are experimentally validated.

### Sample volume derivation

Recommendations regarding the implementation of sampling and subsequent analysis of MPs in drinking water would greatly benefit from an indication regarding the relative relationship between the screening levels derived above and the analytical sensitivity required to reliably detect MPs in drinking water at concentrations consistent with potential screening levels. In the analysis of MPs in drinking water, the volume of water needed to concentrate a sufficient number of MPs to exceed a defined reporting analytical limit represents an important source of information towards guiding monitoring activities (Brander et al. [12]; Koelmans et al., [57]). Detection limits for spectroscopic analysis techniques (e.g., infrared, Raman) are largely limited by particle size, abundance (i.e., count), morphology, and color (related to polymer type) whereas pyrolysis–gas chromatography/mass spectrometry (pyro-GC/MS) is limited by the mass of each polymer present in a sample. At the time of writing, standardized analytical methodologies for microplastics in drinking water are available for spectroscopic analysis via Raman and infrared (Wong [117]; Wong [118]), but standardized protocols for pyro-GC/MS are still under development (see ASTM WK67788 [6]).

While standardized methods for pyro-GC/MS are not currently available, the technique demonstrates potential for being reliable in detecting MPs in drinking water and other matrices in addition to being relatively rapid and inexpensive (DeFrond et al., [27]; Primpke et al., [88]). Commonly-reported limits of quantitation for MPs in aqueous media using pyro-GC/MS range from ~ 0.5 – 4 µg, which are largely dependent on polymer type (Gomiero et al., [41]; Dumichen et al., [33]; Pipkin et al., [86]), with quantitation limits for polystyrene in drinking water estimated to be ~ 1 µg (Jung et al. [56]). Pyro-GC/MS mass-based quantitation limits may be directly compared to the mass-based screening levels derived here without the need for alignments or correction factors. To make toxicologically relevant comparisons of quantitation limits to screening levels, MPs of sizes not expected to be bioaccessible through tissue translocation (i.e., larger than 10 µm) (Rieux et al. [30]; Mowat [75]; Hodges et al. [46]; Jani et al. [52]) should be excluded from analysis using filters (i.e., only analyze < 10 µm particles) or otherwise. Comparison of a quantitation limit for polystyrene (i.e., 1 µg) to the screening level derived using default assumptions with a maximum composite UF of 300 applied (i.e., 0.3 µg · L^−1^ for 0.5 µm polystyrene spheres) results in a suggested minimum sampling volume of 3 L. Direct comparison to the range of screening levels derived here using minimum and maximum assumptions for DWI and RSC and without UFs applied (i.e. 0.0018 to 3000 µg · L^−1^; Table 4) to a polystyrene quantitation limit of 1 µg using pyro-GC/MS results in sampling volumes ranging from 3.3 × 10^–4^ to 555 L. Estimates for required sampling volume for mass-based analyses of MPs may be improved by refining uncertainties in the derivation of the screening level (i.e.; DWI, RSC, POD), as well as characterizing the polymer-dependent toxicities of MPs, and polymer-dependent pyro-GC/MS quantitation limits.

Comparison of size-dependent detection limits (reported in particle count) for spectroscopic techniques (e.g., Raman, infrared) to a monitoring goal for a concentration of MPs of a particular size (e.g., screening level of 5.7·10^5^ particles· L^−1^ of 0.5 μm polystyrene spheres) can be used to inform a desired sampling volume for monitoring. The Minimum Detectable Amount (MDAs) for MPs in drinking water, which are the minimum number of microplastic particles that must be present in a sample to give a specified power 1- β of 0.95 (MARLAP, [70]) are reported for three size fractions using Raman spectroscopy or fourier-transform infrared spectroscopy by DeFrond et al. [27] are 88 particles (> 500 µm), 47 particles (212–500 µm), 80 particles (20–212 µm), and 11 particles (1—20 µm). As described by DeFrond et al. [27], the MDA values are characterized as the number of particles (i.e., particle count) and are independent of the extracted water volume, so are further denoted here as MDA_part_. To support an estimate of the desired sampling volume for drinking water, the ratio of the MDA_part_ and screening level results in a volume of water required to reliably detect the concentration of MPs consistent with the screening level. Ideally, the source of information pertaining to analytical method performance should represent the characterization and quantification of MPs consistent with the size and type of particles used to derive the screening level, which in this instance are 0.5 µm polystyrene spheres. Since the ideal data are currently lacking, we suggest the adoption of the following approximation approach, based on Koelmans et al. [58], which could be used for estimating particle counts for particle sizes that are outside the measured size range. For instance, Koelmans et al. [58] propose the use of a scaling factor to correct the measured particle-count concentration for a given size range (e.g., 1 – 20 µm), which is used to reflect a probabilistic estimate of concentrations reflecting a broader size range, such as 1—5,000 µm, as obtained from Eq. 2.

2 $$C{F}_{Meas\, or\, Bio}=\frac{{L}_{{UL}_{Default}}^{1-a}- {L}_{{LL}_{Default}}^{1-a}}{{L}_{{UL}_{Meas\, or\, Bio}}^{1-a}- {L}_{{LL}_{meas\, or\, Bio}}^{1-a}}$$

Where *L*_*UL*_ and *L*_*LL*_ are the upper and lower particle lengths (µm), respectively, and the subscripts *default*, *meas*, and *bio,* correspond to a default size range (e.g., California’s microplastics definition: 0.001 to 5,000 µm) (State Water Resources Control Board [103]), analytically measured range (e.g., 1—20 µm), and bioaccessible range (e.g., based on tissue translocation potential; < 10 µm), respectively. The slope of the power law for particle length (*a*; unitless) should be applicable to a given size range and environmental compartment (e.g., surface freshwater; treated drinking water) (Koelmans, et al., [58]; Kooi et al., [60]). Use of this rescaling equation assumes that MPs of interest are all MPs regardless of polymer type, shape, and size. Since toxicity information is only available here for polystyrene spheres, caution should be exercised in using these equations until differences in toxicity between sizes and shapes of MPs are known.

To rescale and/or align data using these methods, site-specific distribution data are preferable. Since MP distributions in drinking waters in California have not been characterized at the time of writing, the closest available data may be used. Nizamali J, Mintenig SM, Koelmans AA: Assessing microplastic characteristics in bottled drinking water and air deposition samples using laser direct infrared imaging, in preparation report a MP length exponent value of 2.78 ± 0.29 derived from 286 MPs detected in 9 water bottles with a size-based detection limit of 10 µm. Ideally, a power law value for tap water would be used, as some MPs in bottled water may occur due to contamination by packaging (Sobhani et al. [102]), however such values are currently unavailable. Kooi et al. [60] report a power law exponent of 2.64 ± 0.01 for MPs in surface freshwater—which is commonly used as a source for tap water with additional treatment—and is within one standard deviation of the value reported by Nizamali J, Mintenig SM, Koelmans AA: Assessing microplastic characteristics in bottled drinking water and air deposition samples using laser direct infrared imaging, in preparation. *In lieu* of a site-specific value for tap water in California, the value for bottled water from Nizamali J, Mintenig SM, Koelmans AA: Assessing microplastic characteristics in bottled drinking water and air deposition samples using laser direct infrared imaging, in preparation is used here, with sensitivity assessed using the mean ± the standard deviation. The power law size distribution exponent values from Nizamali J, Mintenig SM, Koelmans AA: Assessing microplastic characteristics in bottled drinking water and air deposition samples using laser direct infrared imaging, in preparation and Kooi et al. [60] are argued to be valid from 1 to 5,000 µm, and reasonably expected to be valid to sizes as small as 0.1 µm according to a re-analysis of data from Mattsson et al. [72] and Lambert and Wagner [61] as reported in Nizamali J, Mintenig SM, Koelmans AA: Assessing microplastic characteristics in bottled drinking water and air deposition samples using laser direct infrared imaging, in preparation.

For the purposes of this exercise, data are aligned and rescaled to a default distribution (i.e., 1 to 5,000 µm) based on the lowest POD as reported for 0.5 µm polystyrene spheres in Hou et al., [48]). Other studies reported here use smaller particles (e.g., 0.04 µm in Amereh et al. [1]), however aligning particles of such small size would be highly uncertain based on available monitoring data and are not attempted here even for demonstration purposes. Using Eq. 2, MDA_part_ for given size ranges reported in DeFrond et al. [27] are rescaled to a default environmentally relevant size range (*LL*_*default*_ = 1 µm; *UL*_default_ = 5,000 µm) using an α value of 2.78 ± 0.29 (95% CI: 2.21 to 3.35) for MPs in bottled water (Nizamali J, Mintenig SM, Koelmans AA: Assessing microplastic characteristics in bottled drinking water and air deposition samples using laser direct infrared imaging, in preparation). Multiplying *CF*_*meas*_ by the MDA_part_ yields the rescaled reporting limits for given size ranges (MDA_part, rescaled_) (Koelmans et al. [58]) and are reported in Table 5.

**Table 5:** Reported and rescaled Minimum Detectable Amounts (MDAs) for MP analysis using Raman or Infrared Spectroscopy reported from DeFrond et al. [27] (95% intervals based on microplastics length distribution power law value in bottled waters from Nizamali J, Mintenig SM, Koelmans AA: Assessing microplastic characteristics in bottled drinking water and air deposition samples using laser direct infrared imaging, in preparation). Desired sampling volumes derived using default mass-aligned screening level without UF’s applied (i.e., 4.6 × 10^6^ particles)

L_LL_, _meas_ (µm)	L_UL_, _meas_ (µm)	MDA (particles)	CF (unitless; 95% CI)	MDA (particles; 95% CI)	Sampling Volume (L; 95% CI)
500	5000	88	6.5 × 10^4^ (2.0 × 10^3^ to 2.2 × 10^6^)	5.7 × 10^6^ (1.7 × 10^5^ to 1.9 × 10^8^)	1.2 (3.8 × 10^–2^ to 42)
212	500	47	1.8 × 10^4^ (1.0 × 10^3^ to 3.4 × 10^5^)	8.3 × 10^5^ (4.8 × 10^4^ to 1.6 × 10^7^)	0.18 (1.0 × 10^–2^ to 3.4)
20	212	80	2.1 × 10^2^ (40 to 1.1 × 10^3^)	1.7 × 10^4^ (3.2 × 10^3^ to 9.1 × 10^4^)	3.7 × 10^–3^ (7.0 × 10^–4^ to 2.0 × 10^–2^)
1	20	11	1.0 (1.0 to 1.0)	11 (11 to 11)	2.4 × 10^–6^ (2.4 × 10^–6^ to 2.5 × 10^–6^)

Similar alignments may be applied to rescale the screening level (based on 0.5 µm polystyrene spheres) to reflect the same environmentally relevant size range. We also note that Koelmans et al. [58] propose a framework for aligning effect concentrations for MPs of varying sizes to reflect a common distribution, and which can be based on the *TRM* (e.g., volume, surface area, etc.) linked to the adverse outcome of interest. In this instance, however, the available data obtained from the mammalian toxicity studies reviewed as part of this exercise were observed to be insufficient to reliably identify a TRM for MPs. Although for nanoparticles, the surface area and specific surface area of particles are believed to be relevant predictors for oxidative stress (Schmid and Stoeger [98]) and inflammation (Tran et al., [109]; Stoeger et al., [106]), respectively, studies linking effects to particle traits amongst the wide range of sizes and polymers in the MPs class are missing. Nevertheless, for illustrative purposes, the application of such an approach is considered in an effort to provide preliminary guidance with respect to the monitoring of MPs in California’s drinking water and should be considered again once the TRM for the adverse effect is determined.

In their approach used to extrapolate adverse effects observed for a monodisperse or polydisperse group of MPs to represent a polydisperse distribution of environmentally relevant MPs, Koelmans et al. [58] and Kooi et al. [60] describe the application of two separate alignments. The first correction enables an alignment to address issues related to bioaccessibility, and which uses Eq. 2 to derive an appropriate scaling factor. Assuming that MPs larger than 10 µm are unlikely to translocate across intestinal barriers to a significant extent (Rieux et al. [30]; Mowat [75]; Hodges et al. [46]; Jani et al. [52]), such particles are considered here to not be bioaccessible and are excluded from alignments. Thus, the limits for the denominator of Eq. 2 are derived accordingly (*LL*_*Bio*_ = 1 µm; *LL*_*bio*_ = 10 µm), which correspond to the smallest default size and the upper limit for bioaccessibility, respectively. This results in a *CF*_*Bio*_ of 1.02 (unitless). Further corrections for the polydispersity of particles in the environment, representing a range of potentially TRMs (volume, surface area, mass, specific surface area) are also applied (see Kooi et al. [60] and Supplementary Information for additional detail). Adopting the approach of Kooi et al. [60] to represent particles between 1 to 5,000 µm in size, various aligned screening levels (particles·L^−1^) are reported in Table 5, and which represent the lowest, highest, and default values for each of the terms applied to Eq. 1 (in order).

**Fig. 5:**
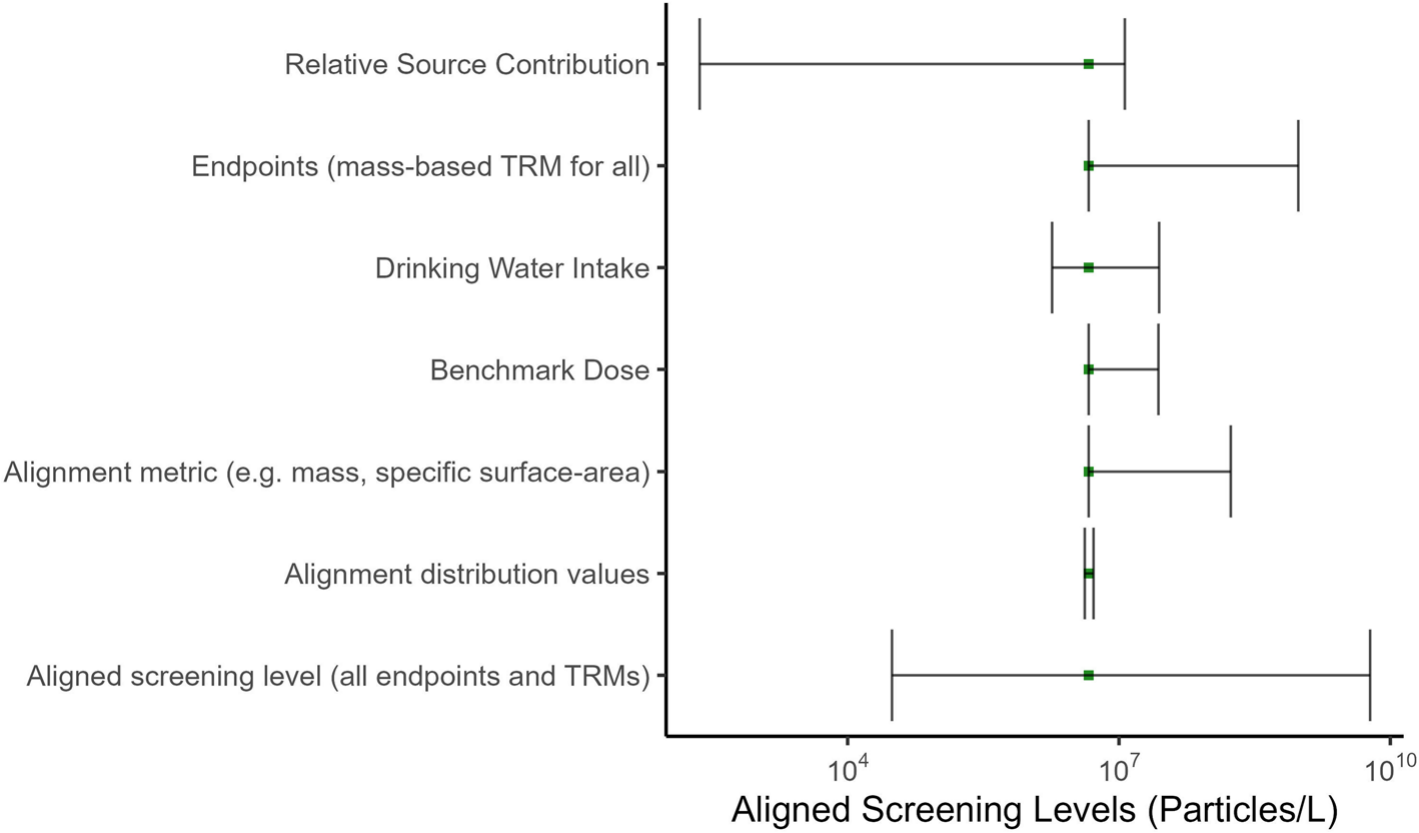
Sensitivity analysis of screening level (aligned to various TRMs to a default size range of 1 to 5000 µm based on one-at-a-time analysis for each variable. For each variable, all other variables were held at their default values, and the minimum and maximum values were used to calculate the resulting lower and upper range for the screening level. The aligned screening level based on endpoint considers all TRMs aligned for all PODs from all reliable endpoints. Green squares represent the screening level obtained using the default value for that parameter, and error bars represent minimum and maximum values of the sensitivity analysis for the variable

Dividing the MDA_part, rescaled_ for given size ranges for Raman and infrared spectroscopy from DeFrond et al. [27] by the range of possible TRM-aligned screening levels results in sampling volumes spanning several orders of magnitude. Using the screening level derived using default assumptions (i.e., RSC = 0.20, DWI = 0.053 L·kg^−1^·d^−1^, lower 95% BMD; composite UF = 1; screening level = 90 µg·L^−1^), and the toxicologically-relevant metric-aligned value of the smallest magnitude which is mass (4.6·10^6^ particles·L^−1^; Table 6) to the MDA_part,rescaled_ for given size ranges results in estimated desirable sampling volumes for drinking water ranging between 2.4 × 10^–6^ L and 1.2 L, with more volume needed when larger size particle ranges are used due to the higher correction factors applied (Table 5). When the minimum and maximum possible TRM-aligned values (Table 6) are considered (i.e. 91 to 6.1 × 10^9^ particles·L^−1^), sampling volumes range from 1.8 × 10^–9^ (1 to 20 µm fraction) to 6.3 × 10^4^ L (500 to 5,000 µm fraction). When the composite UF of 300 is applied to the reference dose (i.e. default mass-aligned screening level = 1.5 × 10^4^ particles·L^−1^), estimated sampling volumes range from 7.2 × 10^–4^ L to 3.7 × 10^2^ L depending on the size fraction analysed. A sensitivity analysis of the aligned screening levels based on MP particle length distributions in drinking water from Nizamali J, Mintenig SM, Koelmans AA: Assessing microplastic characteristics in bottled drinking water and air deposition samples using laser direct infrared imaging, in preparation demonstrates a minor point of uncertainty (0.4 to 20% absolute relative difference) relative to RSC and the POD (Fig. 5).

**Table 6:** Screening levels aligned (1 to 5,000 µm) to various TRMs, with different sensitivity analysis scenarios shown (alignment uncertainty represented lower and upper 95^th^ percentile based on length power law value)

Reference dose^a^ (mg·kg^−1^·d^−1^)	RSC (unitless)	DWI (L·kg^−1^·d^−1^)	Screening Level^a^ (µg·L^−1^)	Mass-aligned Screening Level (particles·L^−1^)	Particle-aligned Screening Level (particles·L^−1^)	Surface area-aligned Screening Level (particles·L^−1^)	Specific surface-area aligned Screening Level (particles·L^−1^)	Volume-aligned Screening Level (particles·L^−1^)
0.14 (BMD)	0.50	0.022	3200	1.6 × 10^8^ (1.5 × 10^8^ to 1.9 × 10^8^)	3.1 × 10^9^ (2.6 × 10^9^ to 3.5 × 10^9^)	1.3 × 10^9^ (1.3 × 10^9^ to 1.3 × 10^9^)	6.1 × 10^9^ (5.5 × 10^9^ to 6.9 × 10^9^)	3.4 × 10^8^ (3.4 × 10^9^ to 3.6 × 10^8^)
0.024 (BMDL)^b^	0.20	0.053	90	4.6 × 10^6^ (4.2 × 10^6^ to 5.2 × 10^6^)	8.6 × 10^7^ (7.5 × 10^7^ to 9.9 × 10^7^)	3.6 × 10^7^ (3.6 × 10^7^ to 3.8 × 10^7^)	1.7 × 10^8^ (1.6 × 10^8^ to 1.9 × 10^8^)	9.6 × 10^6^ (9.5 × 10^6^ to 1.0 × 10^7^)
0.024 (BMDL)	0.0001	0.135	0.0018	91 (82 to 1.0 × 10^2^)	1.7 × 10^3^ (1.5 × 10^3^ to 1.9 × 10^3^)	7.1 × 10^2^ (7.0 × 10^2^ to 7.4 × 10^2^)	3.4 × 10^3^ (3.1 × 10^3^ to 3.8 × 10^3^)	1.9 × 10^2^ (1.9 × 10^2^ to 2.0 × 10^2^)

^a^Reference doses and screening levels reported here are derived with a composite uncertainty factor of one

^b^”Default” parameters for screening level derivation

Given the values of 1,000 L suggested by Koelmans et al. [57] for representatives and 1,500 L required with a standardized method (ASTM D8332-20) are within the range of health-based sampling volumes derived here, we suggest the use of 1,000 L as a reasonable minimum volume of water to target towards helping to support the reliable analysis of MPs in California’s drinking water for most analyses. As detectable size ranges decrease in size, the volume of water needed to achieve desired detection limits decreases exponentially due to the power law size distributions of MPs, as illustrated in Table 5. If water has been treated with processes likely to remove larger MPs, such as sedimentation, flocculation, or filtration (e.g. microfiltration, reverse osmosis, etc.) (Cheng et al., [20]; Wang et al., [115]; Pivokonsky et al., [87]), size-based distributions and sampling volumes estimated based on such distributions (i.e. Nizamali J, Mintenig SM, Koelmans AA: Assessing microplastic characteristics in bottled drinking water and air deposition samples using laser direct infrared imaging, in preparation) may no longer be valid. For assessing MPs in treated water, analysts should ensure that size-based detection limits can adequately characterize the sizes of MPs that pass-through treatment.

The sensitivity of the screening level based on TRMs and endpoint PODs from different studies was also evaluated. For each reliable endpoint POD listed in Table 3, aligned screening levels were calculated based on particle mass, surface area, specific surface area, and volume TRMs according to methods described in the supplemental information and Koelmans et al., [58]). Laboratory effect studies using particles larger than 10 µm were excluded due to their theoretical implausibility for translocation (Rieux et al. [30]; Mowat [75]; Hodges et al. [46]; Jani et al. [52]), and studies using particles smaller than 0.5 µm were excluded due to significant uncertainties with regards to particle size distributions smaller that size range (Kooi et al., [60]). All values were aligned to a default distribution of 1 to 5,000 µm. Since the TRM-aligned values are dependent on the particle size, shape, and density used in the study as well as the POD (dependent on the endpoint, species, and other factors), this combined sensitivity analysis (Fig. S7) illustrates the relative combined impact of these factors. The lowest possible screening level using the default values for RSC and DWI for all PODs and TRMs is 3.1 × 10^4^ particles · L^−1^ aligned by surface area (1 to 5,000 µm) and based on a POD of 0.043 mg·kg^−1^·d^−1^ from Xie et al. [124] using 5 µm polystyrene spheres (Fig. 5). The largest possible screening level using this approach is 6.0 × 10^9^ particles· L^−1^ aligned by volume and based on a POD of 5.0 mg·kg^−1^·d^−1^ from Deng et al. [29] using 5 µm polystyrene spheres (Fig. S7).

As illustrated in Fig. 5, the most influential factors for deriving a screening level using available (limited) evidence are relative source contribution, and a combination of endpoint and TRM. As mentioned above, improved understandings of human exposure to MPs from all sources, more reliable laboratory toxicity studies, and mechanistic information that supports a TRM would reduce the uncertainty of a health-based screening level for drinking water.

### Implications and recommendations

Reliable and safe drinking water is a pillar of society and key to protecting public health. Frameworks for developing standards and guidelines for drinking water have been in place since the WHO first published the International Standards for Drinking-water in 1958, which has been adopted in part or in whole by many countries as a basis for the formulation of national standards (WHO [119]). However, approaches for deriving health-based guidance levels which form the basis of drinking water regulations differ between the United States (including individual states), the European Union, and other nations. While this assessment followed the State of California’s approach to assessing risks of contaminant exposure in drinking water to fulfil the specific goal for informing monitoring in the State required by law (California Code of Regulations [18]), a supplementary analysis following the European Union’s approach to assessing dose–response relationships was performed to demonstrate sensitivities due to jurisdiction-specific modelling preferences (Fig. S6, Table S7). Each regulatory agency has unique policies and legal requirements, and it is at the discretion of each agency on how to use the information presented in this assessment. Based on the evaluation of the existing data, a consensus among workshop participants is to support a non-regulatory screening level for MPs in drinking water.

To protect public health, government agencies such as the US EPA and the OEHHA often derive health-based advisory-levels for emerging contaminants using conservative assumptions regarding hazard and exposure. In this study, the derivation of a non-regulatory screening level for MPs in drinking water included the application of a number of conservative assumptions, which have been adopted and used in the absence of refined scientific evidence. Conservative assumptions include the attribution of 20% RSC of drinking water to total exposure of MPs via an oral exposure pathway, as well as the use of the most sensitive POD and the derivation of a reference dose based on a maximum composite UF of 300. Drinking water intake rates used are based on estimates for susceptible populations in California, which because of the warmer climate and residents’ relatively high activity rate, is a higher estimate than other regions (OEHHA [83]). As illustrated through the use of a sensitivity and uncertainty analysis, the two critical parameters influencing the derivation of the screening level are due to uncertainties associated with the POD and the RSC, with additional relatively minor uncertainty associated with alignments to environmental MP distributions. Thus, in order to reduce inherent uncertainties in the derivation of the non-regulatory screening level value and to support science-based regulatory activities, future research must aim at producing more reliable effects data and strengthen our understanding of exposure to MPs in drinking water, as well as in food and beverages.

Consequently, we recommend that additional research regarding mammalian toxicity of MPs, based on systematic and standard methods, are urgently needed to determine an appropriate regulatory response. At the time of writing, the authors are aware of several significant projects that are underway. Notably, the European Commission is funding five independent projects for a total of ~ €30 million focused on characterizing the human health impacts of micro- and nano-plastics with end dates listed as March 31, 2025 or 2026 (European Commission [23]). In the United States, government-funded research on human health impacts of microplastics are limited, with a single small project ($112,499) identified with a projected end date of August 31, 2021 (National Institute of Health [76]). In Canada, research on the human health impacts of MPs is also limited; the Natural Sciences and Engineering Council of Canada is currently funding one project on the human health impacts of nanoplastics ($1 million CAD), the Government of Canada is funding two small projects through the Increasing Knowledge on Plastic Pollution initiative ($306,515 CAD combined). While these projects will likely fill significant data gaps in the understanding of the effects of microplastics on human health, additional research is necessary to reduce significant uncertainties in assessing risks to humans.

We further note that a number of different endpoints have been reviewed as part of this exercise, however, there appears to be a lack of mechanistic understanding demonstrating why certain endpoints have been targeted by the research community and others have not. We observe that effects on inflammation, for instance, represent a common theme, which we suggest warrants further targeted investigation. It may be beneficial to therefore characterize and quantify potential relationships between the properties of particles and their potential to trigger various inflammatory and oxidative stress biomarkers, from which a stronger mechanistic basis might begin to emerge. A mechanistic understanding may enable reliable alignments across varying particle types, most importantly between particles used in toxicity tests and those that humans are exposed to (Koelmans et al. [58]). Complimentary to targeted mechanistic effect studies are a greater need towards better understanding of the translocation and internalization of MPs, particularly in relation to varying properties of size, shape, and composition. Additional studies on microfibers in mammalian models, for example, would add greatly to our collective understanding of potential effects from real world exposures.

As emphasized by Gouin et al., [42], future toxicity studies should ensure to fulfil the quality and reporting characteristics aligned with the various QA/QC criteria described. For studies targeting a specific endpoint, additional opportunities to ensure studies are conducted according to best practices should be optimized, such as considering criteria defined in the SciRAP (Beronius et al. [9]), used to support the evaluation of mammalian reproductive toxicity studies, for instance. Studies aiming to characterize effects, either through dose–response curves or toxicodynamics, may be informed by summary statistics for lower benchmark dose concentrations (BMDLs), grouped by general endpoint and size as presented in Table 3. Finally, although non-mammalian studies are not typically included in the calculation of regulatory thresholds for humans, given that zebrafish (*Danio rerio*) are now a widely accepted model for the mechanistic study of human diseases, it follows that the numerous studies on the toxicological impacts of micro and nanoplastic exposure could be considered when assessing their potential risk to human health (e.g., Bhagat et al., [10]; Qiang and Cheng, [90]). Additionally, the development of quantitative in vitro to in vivo extrapolation models should be prioritized for MPs to enable the assessment of in vitro studies in a regulatory context (30 of which could not be considered in this assessment) and reduce the number of live animals used in experiments.

## Conclusion

Here we present the findings of a workshop to assess the potential evidence for MPs toxicity to humans through drinking water. We observed that uncertainties are too high to develop or recommend threshold values for any regulatory use, with significant data gaps lacking for exposure and mechanistic understandings of particle toxicity. Despite these shortcomings, we present a framework for developing guidance values for MPs in drinking water and tested the relative sensitivity of the framework with existing (limited) data. Using this framework, we derived a non-regulatory health-based screening level value, which contains a significant level of uncertainty, however, may be used to determine a sampling volume for estimating human exposure via drinking water. We anticipate that refinement in the RSC represents an important factor towards reducing the overall relative uncertainty but are confident that the 1,000 L of drinking water estimated to support monitoring activities represents a reasonable recommendation to California’s water authorities to adopt. When coupled with the acquisition of reliably obtained mammalian toxicity data, which we anticipate will result in an overall improvement of our understanding of adverse effects, the ability to transition from a non-regulatory screening level to regulatory threshold values will undoubtably be more strongly supported in the future.

## Supplementary Information

**Additional file 1.**

## Data Availability

All data generated and analyzed as part of this study are included in this published article and its supplementary information. Toxicity data used in these analyses may also be visualized and downloaded using the online RShiny application ToMEx hosted at https://microplastics.sccwrp.org/ and described in detail in Thornton Hampton L, Lowman H, Coffin S, Darin E, De Frond H, Hermabessiere L, et al: A Living Tool for the Continued Exploration of Microplastic Toxicity, In Press.

## Abbreviations

AOP: Adverse outcome pathway
AMH: Anti-Müllerian hormone
BMD: Benchmark Dose
BMDL: Lower 95% confidence interval of a benchmark dose
BMDU: Upper 95% confidence interval of a benchmark dose
CEC: Contaminant of Emerging Concern
DWI: Drinking water intake
JRC: Joint Research Centre
LOAEL: Lowest-observable adverse effect level
MDA: Minimum Detectable Amount
MPs: Microplastic particles
NOAEL: No-observable adverse effect level
OECD: Organisation for Economic Co-operation and Development
POD: Point of departure
QA/QC: Quality assurance / Quality Control
QIVIVE: Quantitative in vitro to in vivo extrapolation
RIVM: National Institute for Public Health and the Environment
RSC: Relative source contribution
SCCWRP: Southern California Coastal Water Research Project
TRM: Toxicologically relevant metric
UF: Uncertainty factor
US EPA: United States Environmental Protection Agency
WHO: World Health Organization
