# Supplementary material for: Development and application of a health-based framework for informing regulatory action in relation to exposure of microplastic particles in California drinking water
Source: Microplast nanoplast. 2022 May 25;2(1):12. doi: 10.1186/s43591-022-00030-6 (PMC9132802; doi:10.1186/s43591-022-00030-6)
Supplement: Supplementary file 1 — Additional file 1. [file 43591_2022_30_MOESM1_ESM.pdf]

Supporting Information for

Development and application of a health-based framework for informing regulatory action in relation to exposure of microplastic particles in California drinking water

Scott Coffin*, Hans Bouwmeester, Susanne Brander, Pauliina Damdimopoulou, Todd Gouin, Ludovic Hermabessiere, Elaine Khan, Albert A. Koelmans, Christine L Lemieux, Katja Teerds, Martin Wagner, Stephen Weisberg, Stephanie Wright

* Corresponding author: scott.coffin@waterboards.ca.gov

This PDF file includes:

Supplementary Methods
Table S1 to S7
Figure S1 to S7
Power analysis
Outside expert opinions

Other supplementary material for this manuscript includes the following:
(available at https://microplastics.sccwrp.org/)

Data
Interactive visualization tool (ToMEx)
R code

Supplementary Methods

Drinking Water Intake Values

Age-specific drinking water intake (DWI) values were estimated using methods described by OEHHA (2012), from which data obtained from a nationwide survey of food and beverage intake for approximately 20,000 people (US Department of Agriculture's Continuing Survey of Food Intake of Individuals 1994-1996, 1998 dataset) and were normalized to body weight. To estimate the 70-yr lifetime weighted average DWI, average body-weight normalized water intake rates ($L \cdot kg^{-1} \cdot d^{-1}$) for age bins reported in OEHHA (2012) were first multiplied by their fractional duration of a 70-year lifetime and then the sum of these values was used to determine the time-weighted average ($L \cdot kg^{-1} \cdot d^{-1}$). Table S1 lists the probabilistic drinking water intake values for age bins and their associated fractional duration and contributions.

Table S1. Probabilistic drinking water intake values used in sensitivity analysis

**Life Stage**	**Age range (years)**	**Fractional duration (years)**	**Median oral ingestion (L/kg-day)**	**Upper 95th% oral ingestion (L/kg-day)**	**Max oral ingestion (L/kg-day)**
3rd Trimester (pregnancy)	NA	0.75/70	0.018	0.047	0.117
Infant	0-2	2/70	0.113	0.196	0.491
Child	2 - 16	14/70	0.024	0.061	0.152
Adult	16-70	54/70	0.018	0.045	0.116
Time-weighted average (L-kg/day)			0.022	0.053	0.135

Alignment of Monodisperse Effect Concentrations to Polydisperse Environmental Concentrations

Monodisperse laboratory-based effect concentrations (e.g. 5 µm polystyrene [PS] spheres) were re-scaled to a default polydisperse particle size range (e.g. 1 - 5,000 µm) using methods described in Kooi et al (2021) for the purposes of this assessment. Re-scaling to a default size range allows direct comparison to exposure concentrations for a default size range (which may also be re-scaled). To rescale to a toxicologically relevant metric (TRM) of interest, the effect concentration must first be corrected for bioaccessibility. In the case of microplastics in drinking water, tissue uptake (which is hypothesized to be critical to adverse effects) is size-restricted (Mohamed Nor et al., 2021). A size-based restriction for tissue uptake may be calculated as the fraction of particles between the bioaccessible size fraction (i.e. 1 - 10 µm) in the default size range (e.g. 1 - 5,000 µm) (Rieux et al 2005; Mowat 2003; Hodges et al 1995; Jani et al 1992).

Below is an illustrated example of the alignments performed on a given effect concentration derived in the method sections of the main manuscript. As an example, Hou et al (2021a) reported a NOAEL of 0.17 mg/kg-day for sperm deformity in male mice, from which a drinking water screening level of 640 µg/L for monodisperse 5 micron PS spheres may be

derived using equation 1 in the main text. To convert the monodisperse effect concentration from mass/volume to particles/volume, the effect concentration may be divided by an estimated particle mass for 5 µm PS spheres using the volume equation for a sphere ($V = 4/3*\pi*r^3$) and a density for polystyrene of 1.05 g/cm^3 (Cospheric 2018). This results in an estimated particle mass of 6.87 x 10^{-5} µg/particle. The monodisperse effect concentration in particles/L is therefore estimated to be 9.33 x 10^6 particles/L.

Given an upper limit (UL) and lower limit (LL) of the measured and default size range, a dimensionless correction factor (CF_{meas}) for measured environmental concentrations or (CF_{bio}) for bioaccessible effect concentrations may be calculated, which rescales the measured (M) or bioaccessible (B) number concentrations for a certain size range to the number concentration for the microplastics default (D) size range (e.g. 1 to 5,000 um) according to the power law distribution for length (L) in the compartment of interest (e.g., drinking water).

The equation for CF_{meas} (equation S1) is identical for effect concentration (CF_{Bio}) except the bioaccessible fraction of particles is denoted as UL,B and LL,B on the denominator (for the case of tissue translocation, 1 - 10 microns) (Koelmans et al 2020).

Equation S1 $$CF_{Meas} = \frac{L_{UL,D}^{1-a} - L_{LL,D}^{1-a}}{L_{UL,M}^{1-a} - L_{LL,M}^{1-a}}$$

The resulting correction factor (CF_{meas}, unitless) is then multiplied by the measured concentration (C_{Meas}, particles/L) to obtain a rescaled exposure number concentration (particles/L) using equation S2.

Equation S2 $$C_{Env} = CF_{meas}\, x\, C_{meas}$$

The monodisperse effect concentration in particles/L for a theoretical SL (e.g., 5 um PS spheres) is corrected to an environmental polydisperse distribution of 1-5,000 um microplastics by first calculating CF_{Bio} using equation S3.

Equation S3 $$CF_{Bio} = \frac{L_{5,000}^{1-a} - L_{1}^{1-a}}{L_{10}^{1-a} - L_{1}^{1-a}}$$

Using an $\alpha_L$of 2.78 for microplastics in bottled waters (Nizamali et al *in prep*), the CF_{Bio} in equation 3 would equate to 1.02 (unitless).

The threshold effect concentration for the bioaccessible size fraction of particles (EC_{poly}; particles/L) can be related to the threshold effect concentration for the environmentally relevant (1 to 5,000 um) range of particles (EC_{env}; particles/L) with equation S4.

Equation S4 $$EC_{env} = EC_{poly} * CF_{bio}$$

In order to convert the monodisperse microplastic particle effect concentration a second correction must occur, which takes into consideration the toxicologically relevant metric (TRM) (Koelmans et al 2017). For a given TRM (*x*), the threshold may be related to both mono- or polydisperse particles interchangeably so long as the total magnitude of TRM remains the same (Koelmans et al, 2020). Kooi et al (2021) provide an equation which preserves the TRM of interest (i.e. SSA) between mono- and polydisperse particles for a given threshold effect level (equation S5).

Equation S5 $EC_{poly} * \mu_{x,poly} = EC_{mono} * \mu_{x,mono}$

Where EC_{mono} (particles/L) is the effect number concentration for monodisperse particles and $\mu_{x,mono}$ is the mean value for the TRM of interest for the monodisperse particles. The above equation may be used to convert both monodisperse and polydisperse distributions with any level of polydispersity. Equations for calculating specific TRMs with an example SL based on a theoretical laboratory experiment that used 5 µm PS spheres follows.

Surface Area TRM

For surface area as an TRM, $\mu_{sa,mono}$ is equivalent to the average surface area of a 5 µm PS sphere, calculated as follows (equation S6):

Equation S6 $SA = 4 * \pi * r^2$

Since the probability distribution of TRM sa (surface area) follows a power law regime, the mean TRM value for the polydisperse particles, $\mu_{sa,poly}$, can be calculated using equation S7:

Equation S7 $\mu_{sa,poly} = \frac{1-a_{sa}}{2-a_{sa}} \frac{X_{UL}^{2-a_x} - X_{LL}^{2-a_x}}{X_{UL}^{1-a_x} - X_{LL}^{1-a_x}}$

Where UL and LL are respectively defined as the upper and lower limit in TRM sa for which the mean is calculated, and a_{sa} is the power law exponent of TRM sa. In the case of a = 2 (the a_{sa} for the area of microplastics in surface freshwater from Table S4 in Kooi et al 2021), an alternative equation (equation S8) must be used (Kooi et al 2021):

Equation S8 $\mu_{sa,poly} = \frac{ln(\frac{X_{UL}}{X_{LL}})}{X_{LL}^{-1} - X_{UL}^{-1}}$

For surface area, UL and LL are calculated using the equation for the surface area (SA) of an ellipsoid (equation S9):

Equation S9 $SA = 4\pi(\frac{(ab)^{1.6} + (ac)^{1.6} + (bc)^{1.6}}{3})^{1/1.6}$

With *a, b, c* being equal to 0.5 x length, 0.5 x width, and 0.5x height, respectively. The same minimum and maximum bioaccessible values for length, width, and height are used. In the case of a 5 µm spherical PS particle example, the ranges for length, width and height are 1 to 10 µm, 1 to 10 µm, and 1 to 10 µm, respectively. The *minimum* bioaccessible particle area is thus calculated from the dimensions (*a, b, c*): 0.5, 0.5, 0.5 µm, and the *maximum* ingestible particle area is calculated from the dimensions 5, 5, and 5 µm. Using the above equation for the surface area of an ellipsoid results in minimum and maximum bioaccessible particle areas of x_{UL}= 387.4 um^2 and x_{LL} =2.48 um^2, respectively. Substitution of these x_{UL} and x_{LL} values in equation S7 results in: $\mu_{SA,poly} = 12.6\, um^2$.

The polydisperse concentration therefore is calculated by rearranging equation 7 into equation S10:

Equation S10 $EC_{poly} = \frac{EC_{mono} * \mu_{x,mono}}{\mu_{x,poly}}$

For the 5 µm PS sphere example, $EC_{poly,SA}$= 2.4 x10^8 particles/L. Using equation S4, $EC_{env,SA}$= 2.41 x10^8 particles/L.

Volume TRM

In the case of an TRM of interest being total volume, $\mu_{x,mono}$ is equivalent to the average volume of a 5 µm PS sphere (i.e. $\mu_{v,mono}$). The volume of a sphere can be calculated using the simple equation for a sphere (equation S11).

Equation S11 $$V = \frac{4}{3}\pi r^3$$

Where r = radius (length/2 = 2.5 µm for 5 µm spheres).

For the example of a 5 um PS sphere, $\mu_{v,mono}$= 65.4 um^3.

Since the probability distribution of TRM v (volume) follows a power law regime, the mean TRM value for the polydisperse particles, $\mu_{V,poly}$, can be calculated using equation S7. Where UL and LL are respectively defined as the upper and lower limit in TRM v (volume) for which the mean is calculated, and a_v is the power law exponent of mass. In the case of freshwater surface water, an a_v of 1.68 ± 0.081 is utilized (Kooi et al 2021).

For volume as a TRM, UL and LL are volume-based upper and lower limits, respectively of bioaccessibility based on the length of particles. To estimate volume-based limits based on particle length, the volume of bioaccessible particles (1-10 µm) is first calculated using an expanded version of equation S11 (equation S12) which estimates volume for elongated spheres (Koelmans et al 2020).

Equation S12 $$V_i = \frac{4}{3}\pi abc$$

Where V_i is the volume for a given particle *i*, and *a*, *b*, and *c* are radii along the principal axes, corresponding to one-half times the length, width, and height of an ellipsoid.

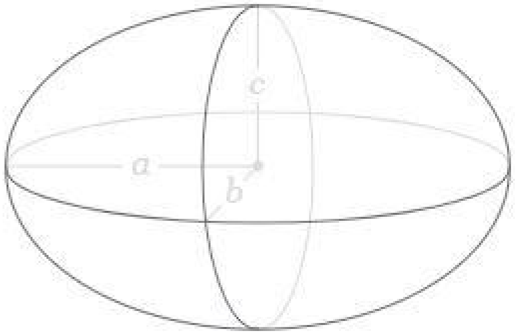

Figure of an ellipsoid with principal radii shown (a, b, c).

The above equation can be applied to fragments, thin films, microbeads, spheres, or fibers given a known length to width ratios for such shapes, with the height assumed to be equivalent to 0.67 x width which is the average for environmental microplastics (Kooi et al 2021). Width to length ratios differ for microplastics differ by compartment, with averages

ranging from 0.67 to 0.77 (Kooi et al 2021). Averaged values may be used to estimate the volume of polydisperse environmental mixtures of microplastics.

In the case of a 5 um spherical PS particle example, the bioaccessible ranges for length are 1 to 10 µm. Using the above equation for the volume of an ellipsoid results in minimum and maximum bioaccessible particle volumes of: x_{UL}= 157.48 um^3 and x_{LL} =0.16 um^3 .Substitution of these x_{UL} and x_{LL} values in the above equation results in $\mu_{v,poly} = 2.74\ um^3$The polydisperse concentration for a volume-based TRM therefore is calculated using equation 12. For the 5 µm PS sphere example $EC_{poly,v}$= 2.3 x10^8 particles/L.

Mass TRM

In the case of a TRM of interest being total mass, $\mu_{x,mono}$ is equivalent to the average mass of a 5 µm PS sphere (i.e. $\mu_{m,mono}$), calculated using equation S13.

Equation S13 $$m = pV * \frac{1}{1e12} * 1e6$$

Where *m* is the mass (µg), *p* is density (g/cm^3; 1.05 for polystyrene), *V* is volume (µm^3) which is calculated by the cube of the radius of each particle (i.e. 1/2 * length, or 2.5 µm), and additional conversion factors for g to µg (1x10^6) and cm^3 to um^3 (1x10^{-12}). For the example of a 5 µm PS sphere, $\mu_{m,mono}$= 6.87 x 10^{-5} µg.

Since the probability distribution of ERM m (mass) follows a power law regime, the mean ERM value for the polydisperse particles, $\mu_{m,poly}$, can be calculated using equation S7. Where UL and LL are respectively defined as the upper and lower limit in ERM m (mass) for which the mean is calculated, and a_m is the power law exponent of mass. In the case of freshwater surface water, an a_m of 1.65 ± 0.071 is utilized (Kooi et al 2021).

For mass, UL and LL are mass-based upper and lower limits of bioaccessibility based on the width of particles, respectively. To estimate mass-based limits based on size, the volume of bioaccessible particles (1-10 µm) is first calculated using the equation for the volume of an ellipsoid, then multiplied by the average density of particles in the 1-5,000 um distribution in surface freshwater (1.04 g/cm^3) (Kooi et al 2021).

Equation S14 $$X_{LL/UL} = pV * \frac{1}{1e12} * 1e6$$

Where $X_{LL/UL}$ is the lower/upper limit of bioaccessible mass (µg), *p* is density (g/cm^3), *V* is volume (µm^3), and additional conversion factors for mg to g (1000) and cm^3 to µm^3 (1x10^{-12}).In the case of tissue translocation, the bioaccessible ranges for length are 1 to 10 µm based on the lower default range and the upper translocatable size (Rieux et al 2005; Mowat 2003; Hodges et al 1995; Jani et al 1992).

Using the above equation for the volume of an ellipsoid and an average density of 1.04 g/cm^3 results in minimum and maximum bioaccessible particle mass of x_{UL} = 8.7x10^{-5} µg and x_{LL}= 8.7x10^{-8} µg. Substitution of these x_{UL} and x_{LL} values in equation S7 results in $\mu_{m,poly} =$ 1.67x10^{-6} µg. The polydisperse concentration for a mass-based TRM is again calculated using equation 12. For the 5 µm PS sphere example, $EC_{poly,m}$= 3.9 x10^8 particles/L.

Specific Surface Area TRM

In the case of an TRM of interest being specific surface area, $\mu_{ssa,mono}$ is equivalent to the surface area of a 5 µm PS sphere (i.e. $\mu_{sa,mono}$) divided by the mass (i.e. $\mu_{m,mono}$), calculated as follows (equation S15):

Equation S15 $$SSA = \frac{SA}{m}$$

Where *SA* is the surface area (µm^2) of the particle (5 µm PS sphere), and *m* is the mass (µg).

Since the reciprocal of the probability distribution of ERM ssa (specific surface area) follows a power law regime, the mean TRM value for the polydisperse particles, $\mu_{ssa,poly}$, can be can be calculated by first calculating $\mu_{1/ssa,poly}$ then taking the inverse to obtain the $\mu_{ssa,poly}$ using equation S15. In equation S2, X_{UL} and X_{LL} are respectively defined as the inverse of the upper and lower limit in ERM ssa (i.e. 1/ssa) for which the mean is calculated, and $a_{1/ssa}$ is the power law exponent of the inverse of the specific surface area. In the case of freshwater surface water, the inverse specific surface area power law ($a_{1/ssa}$) of 2.71 ± 0.009 is utilized (Kooi et al 2021).

For specific surface area, X_{UL} and X_{LL} correspond (respectively) to the specific surface area of the upper and lower limits of bioaccessible particles based on particle length as a limiting factor. To calculate the specific surface area of the upper and lower bioaccessible particles (determined by length for tissue translocation) within the 1-5,000 µm distribution, the translocatable surface area limits are divided by the translocatable mass-based limits as calculated above.

$\mu_{1/SSA,poly}$ may now be calculated given these limits, using an alpha value of $a_{1/ssa}$ of 2.71 for the freshwater surface water environment (Kooi et al 2021). Note that 1/SSA is used in equation S7, so the inverse must be taken to derive $\mu_{SSA,poly}$, which equates to 6.5 x 10^{-8} µm^2/µg. Now that $\mu_{ssa,poly}$, $\mu_{ssa,mono}$, and EC_{mono} are known for this example for specific surface area, the *bioaccessible polydisperse* effect concentration EC_{poly} for the specific surface area ERM may then be calculated, equivalent to: 1.6 x 10^7 particles/L.

Benchmark Dose Modelling Sensitivity Analysis

As a sensitivity analysis of the impact of benchmark dose modelling methods in the derived PODs, output from EPA's BMDS software was compared to the RIVM National Institute for Public Health and the Environment's PROAST software version 70.3 using R programming language (https://www.rivm.nl/en/proast). In the case of PROAST derivations, confidence intervals were calculated using a model averaging approach as described in Slob (2018). Benchmark dose modelling results from PROAST and BMDS were compared using linear regression for endpoints deemed 'reliable' and 'somewhat reliable' by experts with both recommended BMD models by BMDS and, when no model was recommended by BMDS, the best fit model was used for comparison-sake only (Figure S6).

Comparison of benchmark doses from PROAST and BMDS by linear regression for 'reliable' and 'somewhat reliable' endpoints resulted in a poor, non-significant linear correlation

when all benchmark doses were used, including those deemed unfit by the BMDS software (R^2= 0.02, p = 0.61) (Figure S7A). When comparing benchmark doses from PROAST and BMDS software for endpoints exclusively deemed ‘reliable’ by experts and using models deemed viable by BMDS software, a strong and significant correlation was found (R^2= 0.97, p = 2 x10^{-6}) (Figure S7B).

Supplementary Tables

Table S1. Summary of all mammalian studies for microplastics found through literature search

doi	authors	year	Exposure category	Average Particle Length (um)	polydisperse	Polymer
10.1039/c9tx00147f	Amereh	2019	Ingestion	0.03892	monodisperse	PS
10.1016/j.envpol.2020.114158	Amereh	2020	Ingestion	0.03892	polydisperse	PS
10.1016/j.tox.2020.152665	An	2021	Ingestion	0.5	monodisperse	PS
10.1007/s12013-015-0705-6	Barshtein	2016	In Vitro	0.14645	polydisperse	PS
10.1016/j.jhazmat.2020.123308	Choi	2020	In Vitro	15	polydisperse	PS
10.1039/c9en00523d	Cortes	2020	In Vitro	0.210855	polydisperse	PS
10.1016/j.jhazmat.2020.123263	daCostaAraujo	2020	Ingestion	35.46	monodisperse	PE
10.1038/srep46687	Deng	2017	Ingestion	5	monodisperse	PS
10.1016/j.jhazmat.2018.06.017	Deng	2018	Ingestion	0.75	polydisperse	PS

10.1016/j.envint.2020.105916	Deng	2020	Ingestion	50	polydisperse	PE
10.1016/j.jhazmat.2019.121575	Dong	2020	In Vitro	4.06	polydisperse	PS
10.1039/c7an00461c	Efeoglu	2017	In Vitro	0.1	monodisperse	PS
10.1016/j.tiv.2015.11.006	Forte	2016	In Vitro	0.05455	monodisperse	PS
10.1016/j.biomaterials.2016.01.064	Fuchs	2016	In Vitro	0.117	monodisperse	PS
10.1002/jbmr.5650080907	Glant	1993	In Vitro	0.32	monodisperse	PMMA
10.1067/mlc.2001.114677	Gorbet.Sefton	2001	In Vitro	0.45	monodisperse	PS
10.1002/jbm.a.30354	Gorbet.Sefton	2004	In Vitro	0.45	monodisperse	PS
10.1046/j.1525-1594.2003.07107.x	Gourlay	2003	In Vitro	NA	monodisperse	PVC
10.1016/0142-9612(96)83279-0	Gretzer	1996	In Vitro	1	monodisperse	PS
10.1016/S0142-9612(01)00290-3	Gretzer	2002	In Vitro	1	monodisperse	PS
10.1016/j.scitotenv.2020.138183	He	2020	In Vitro	0.05	polydisperse	PS
10.1016/j.tiv.2019.104610	Hesler	2019	In Vitro	0.0463	monodisperse	PS

10.1016/j.jhazmat.2020.124028	Hou	2020	Ingestion	5.011	monodisperse	PS
10.1016/j.ecoenv.2021.112012	Hou	2021	Ingestion	0.5	monodisperse	PS
10.1016/j.scitotenv.2019.05.071	Hwang	2019	In Vitro	13.5	monodisperse	PP
10.1007/s10904-013-9856-3	Irfan	2013	In Vitro	5.002	polydisperse	PA
10.1016/j.ecoenv.2021.112345	Jiang	2021	Ingestion	5	monodisperse	PS
10.1016/j.scitotenv.2018.08.353	Jin	2019	Ingestion	5	monodisperse	PS
10.1016/j.jhazmat.2020.123430	Jin	2020	Ingestion	0.5	monodisperse	PS
10.1021/es900754q	Kawata	2009	In Vitro	0.015	monodisperse	PS
10.7546/CRABS.2018.10.07	Koprinarova	2018	In Vitro	0.05	monodisperse	PS
10.1007/s00204-020-02750-1	Lehner	2020	In Vitro	76	polydisperse	PA
10.1007/s11356-021-13911-9	Li	2021 b	Ingestion	0.5	monodisperse	PS
10.1016/j.chemosphere.2019.125492	Li	2020 a	Ingestion	80	polydisperse	PE
10.1016/j.envpol.2020.115025	Li	2020 b	Ingestion	0.5	monodisperse	PS

10.1016/j.jhazmat.2020.123933	Li	2021a	Ingestion	4.98	monodisperse	PS
10.1016/j.biomaterials.2011.07.037	Liu	2011	In Vitro	0.05714	monodisperse	PS
10.1016/j.scitotenv.2018.03.051	Lu	2018	Ingestion	0.5	monodisperse	PS
10.1016/j.envpol.2019.113122	Luo	2019a	Ingestion	0.5	monodisperse	PS
10.1021/acs.est.9b03191	Luo	2019b	Ingestion	5	monodisperse	PS
10.1007/s10565-021-09616-x	Merkley	2021	In Vitro	10	monodisperse	PS
10.2147/IJN.S161369	Meszaros	2018	In Vitro	0.1943	monodisperse	PS
10.1016/j.toxlet.2020.01.008	Park	2020	Ingestion	16.9	monodisperse	PE
10.1166/jnn.2019.16347	Phuc	2019	In Vitro	NA	monodisperse	PS
10.1016/j.chemosphere.2017.11.076	Rafiee	2018	Ingestion	0.03892	polydisperse	PS
10.1016/j.envres.2017.08.043	Schirinzi	2017	In Vitro	5.02	polydisperse	PS
10.1016/j.jhazmat.2021.125962	Shengchen	2021	Ingestion	0.005	polydisperse	PS
10.1007/s00204-019-02478-7	Stock	2019	In Vitro	1.05	monodisperse	PS

j.ecoenv.2021.112340	Sun	2021	Ingestion	5	polydisperse	PE
10.1016/j.ecoenv.2021.112296	Sun	2021	Ingestion	5	monodisperse	PS
10.1002/pat.3749	Vorotnikova	2015	In Vitro	0.85	monodisperse	PS
10.1016/j.jhazmat.2020.124536	Wang	2020	In Vitro	0.5	monodisperse	PS
10.1289/EHP7612.	Wang	2021	Ingestion	2	monodisperse	PS
10.1002/tox.23095	Wei	2020	Ingestion	0.5104	monodisperse	PS
10.1080/17435390.2016.1218080	Whitewell	2016	In Vitro	0.0531	monodisperse	PS
10.1016/j.chemosphere.2019.01.056	Wu	2019	In Vitro	0.16	monodisperse	PS
10.1002/tox.22885	Wu	2019	In Vitro	5	monodisperse	PS
10.1016/j.ecoenv.2019.110133	Xie	2020	Ingestion	5.45	polydisperse	PS
10.1016/j.jhazmat.2021.126092	Xu	2021	Ingestion	0.1	monodisperse	PS
10.1016/j.scitotenv.2019.133794	Xu	2019	In Vitro	0.025	monodisperse	PS
10.1016/j.scitotenv.2020.143085	Zheng	2021	Ingestion	5	monodisperse	PS

Table S2. Additional summary of all *in vivo* mammalian studies for microplastics found through literature search

**Experimental Design**		**Particle Characterization**	**Biokinetics**		**Toxicodynamics**		
**Study type and age of animals used**	**Exposure scheme**	**Microplastic type, and concentration**	**Presence of microplastic in (target) organ**	**MNP quantification method**	**Tissues, endpoint**	**results**	**comments**
Jani et al., 1989 Female Sprague Dawley rats	Oral gavage 10 days	100, 500, 1,000 and 3,000 nm non-ionic, carboxylated fluorescent polystyrene spheres 1.5% w/v (1.25 mg/kg-day)	Non-ionized PS spheres (120, 950 nm) showed strong uptake in Peyer's patches, mesentery node; moderate uptake in colon, liver, low uptake in small intestine, stomach. No uptake in kidney, heart or lungs. Negatively charged (carboxylated) PS spheres showed low uptake in Peyer's patches, colon, liver; very low uptake in Stomach, Small Intestine. No uptake in Spleen, Kidney,	Fluorescence microscopy			

**Experimental Design**		**Particle Characterization**	**Biokinetics**		**Toxicodynamics**		
**Study type and age of animals used**	**Exposure scheme**	**Microplastic type, and concentration**	**Presence of microplastic in (target) organ**	**MNP quantification method**	**Tissues, endpoint**	**results**	**comments**
			Heart, Lungs				

Jani et al, 1990	Oral gavage 1.25 mg/kg (0.1 ml volume) daily for 10 days.<br><br>Animals were sacrificed 2 days after last dose.<br>Female Sprague Daley rats (15-20 weeks old).<br><br>100 and 1 μm nm PS MNP also ^{125}I labelled materials were used 0.4 ml (3.2 x10^{12} particles; 2.00 mg/kg) by oral gavage for 8 days	50, 100, 300, 500 nm and 1 μm, 3 μm non-ionized PS MNP with covalently lined fluorescein from Polyscienes Ltd.	Here only the results of 1 and 3 μm PS MNP are listed.<br>1 μm MNP were detected in stomach (percent of administered dose) (0.27 ± 0.12 stomach<br>1.082 ± 0.12 PPs<br>2.43 ± 0.46 Colon<br>0.54 ± 0.03 Liver<br>0.24 ± 0.01 Spleen<br>Not detected in other tissues<br>4.562 ± 0.73 total)<br><br>For the 3 μm<br>1.376 ± 0.08 stomach<br>3.627 0.05 PPs (potentially explained by adsorption)<br>7.53 ± 0.5 Colon (no systemic uptake)<br><br>Labelled expserimens suggest minimal	MNP were extracted from stomach, small intestinal segments, colon, liver, spleen, heart, kidney and lungs.<br>Recovery of extraction was assessed.	Not studied	Not studied	

			systemic availability of MNPs				

**Experimental Design**		**Particle Characterization**	**Biokinetics**		**Toxicodynamics**		
**Study type and age of animals used**	**Exposure scheme**	**Microplastic type, and concentration**	**Presence of microplastic in (target) organ**	**MNP quantification method**	**Tissues, endpoint**	**results**	**comments**
Amereh et al., 2020<br>Male, Wistar rats<br>60 days old (180 ± 10 g)<br>·<br>10.1016/j.envpol.2020.114158	Oral gavage during 35 days.<br>Control, 1, 3, 6 and 10 mg/kg bw-day.	- 'virgin' PS MNPs 25 and 50 nm, Kisker Biotech, MNP-0.025, 1%w/v 10ml and MNP-0.05 2.5% 15 ml. deinonized water (in a binary mixture).<br>-Also fluorescently labelled, no further information.<br>No physical characterisation of MNPs was performed.<br>No chemical analysis (of MNP associated chemicals)	Presence of 1 and 6 mg/kg day fluorescent particles was studied	Whole body scan of two rats are shown that indicate high concentrations in testes (only)	Hormones: LH, FSH, T<br>Semen quality<br>Testicular histology<br>RT-qPCR in testis<br>Fluorescent imaging (1 and 6 mg/kg day)	- T lower than control at all doses; LH dose related reduction; FSH lower in 1, 3 and 6 but bot 10 mg/kg groups<br>- dose related reduction in sperm concentration, effects on sperm viability.<br>- only few histology pictures provided, no information on analysis<br>- gene expression: control data is not	Qualitative analysis of MNP occurrence. Images are not quantified nor explained. Materials for biokinetics are different from the MNPs used in the toxicodynamics experiments.

Experimental Design		Particle Characterization	Biokinetics		Toxicodynamics		
**Study type and age of animals used**	**Exposure scheme**	**Microplastic type, and concentration**	**Presence of microplastic in (target) organ**	**MNP quantification method**	**Tissues, endpoint**	**results**	**comments**
						shown, often 10 mg/kg group is distinct.<br>- On imaging: specific localization in testis, not in rest of body	

**Experimental Design**		**Particle Characterization**	**Biokinetics**		**Toxicodynamics**		
**Study type and age of animals used**	**Exposure scheme**	**Microplastic type, and concentration**	**Presence of microplastic in (target) organ**	**MNP quantification method**	**Tissues, endpoint**	**results**	**comments**
Xie et al., 2020 Male Balb/c mice 5-6 weeks old, 20-25 gram 10.1016/j.ecoenv.2019.110133	Oral gavage for 42 days Control, 100 mg/kg bw/day NAC; 5 mg/kg bw/day SB203580; 0.01 mg/day PS; 0.1 mg/day PS; 1 mg/day PS; 1 mg/day PS + NAC; 1 mg/day + SB203580 Estimated dose: 0.43, 4.25, 43.77 mg/kg-day	5.0-5.9 µm PS MNP, Shanghai Macklin Biochemical Co, Ltd. Ultrapure water N-Acetyl Cysteine (NAC) SB203580 a p38 MAPK-inhibitor. No physical characterisation of MNPs was performed. No chemical analysis (of MNP associated chemicals)	Biokinetics not studied	Biokinetics not studied	Blood, testes, sperm counts and quality, oxidative stress in testes	-no quantification of histology, only 1 picture per group -reduced sperm counts in all MNP groups, dose related increased rate of teratosperm Reduced T-levels in plasma (slight dose dependency)	No biokinetics data, thus no amount of MNP in testes provided. No histological quantification. MNPs were not characterized.

Experimental Design		Particle Characterization	Biokinetics		Toxicodynamics		
**Study type and age of animals used**	**Exposure scheme**	**Microplastic type, and concentration**	**Presence of microplastic in (target) organ**	**MNP quantification method**	**Tissues, endpoint**	**results**	**comments**
Hou et al., 2021<br>#<br>Wistar rats<br>6 weeks old<br><br>https://doi.org/10.1016/j.ecoenv.2021.112012	Separately housed, drinking water for 90 days<br>Control, 0.015, 0.15 and 1.5 mg/day	0.5 µm PS MNPs from Tianjin Baseline ChromTech research Center<br>Limited physical characterisation of MNPs was performed.<br>No chemical analysis (of MNP associated chemicals)	TEM pictures that point to MNPs in ovary of 0.15 mg/d group	TEM	Histology<br>Oxidative stress markers in ovaries<br>Panel of cytokines<br>Apoptotic markers	Number of growing follicles was reduced dose related (starting from 0.15 mg/d<br>Dose related effects on oxidative stress markers, cytokines and apoptosis markers	TEM<br>Occurrence was only show in 1 ovary of 1 exposure group.<br>Usually immunohistochemistry is very challenging to quantify robustly.<br>In TEM picture MNP is shown as a dark spot<br>Question is a MNP visible (electron dense?

**Experimental Design**		**Particle Characterization**	**Biokinetics**		**Toxicodynamics**		
**Study type and age of animals used**	**Exposure scheme**	**Microplastic type, and concentration**	**Presence of microplastic in (target) organ**	**MNP quantification method**	**Tissues, endpoint**	**results**	**comments**
An et al., 2021 #<br>Wistar rats<br>6 weeks old<br><br>https://doi.org/10.1016/j.tox.2020.152665	Separately housed, drinking water for 90 days<br>Control, 0.015, 0.15 and 1.5 mg/day	0.5 µm PS MNPs from Tianjin Baseline ChromTech research Center<br>Limited physical characterisation of MNPs was performed.<br>No chemical analysis (of MNP associated chemicals)	TEM pictures that point to MNPs in ovary of 0.15 mg/d group	TEM	Histology<br>Oxidative stress in ovaries and primary cells<br>Collagen fibers<br>Fibronectin<br>Apoptotic markers	Number of growing follicles was reduced dose related (significant in highest dose group)<br>Dose related effects on oxidative stress (significant from 0.15 mg/d group)<br>Increased optical densities for collagen formation and fibronectin expression (signf in 1.5 mg/d)<br>From 0.15 mg/d exposure	Occurrence was only show in 1 ovary of 1 exposure group.<br>Usually immunohistochemistry is very challenging to quantify robustly.<br>In TEM picture MNP is shown as a dark spot<br>Question is a MNP visible (electron dense?

Experimental Design		Particle Characterization	Biokinetics		Toxicodynamics		
Study type and age of animals used	Exposure scheme	Microplastic type, and concentration	Presence of microplastic in (target) organ	MNP quantification method	Tissues, endpoint	results	comments
						group signf. increased staining of apoptosis markers	

**Experimental Design**		**Particle Characterization**	**Biokinetics**		**Toxicodynamics**		
**Study type and age of animals used**	**Exposure scheme**	**Microplastic type, and concentration**	**Presence of microplastic in (target) organ**	**MNP quantification method**	**Tissues, endpoint**	**results**	**comments**
Li et al., 2020 #<br>Wistar rats<br>6 weeks old<br><br>https://doi.org/10.1016/j.envpol.2020.115025	Separately housed, drinking water for 90 days<br>0.5 mg/L (about 7.18 x 10^{9} particle/L)<br>5 mg/L (about 7.18 x 10^{10} particle/L)<br>50 mg/L (about 7.18 x 10^{11} particle/L)<br>Shaken by ultrasound for 20 min<br>8 hours fasting before sacrifice	0.5 µm PS MNPs from Tianjin Baseline ChromTech research Center<br>Limited physical characterisation of MNPs was performed.<br>No chemical analysis (of MNP associated chemicals)	TEM pictures that point to MNPs in cardiac cells	TEM	Oxidative stress<br>Collagen formation<br>Apoptosis<br>Wnt/b-catenin pathway	Dose related effects on CAT, GSH, MDA and SOD<br>Dose related increased optical densities in slides stained for apoptosis and fibronectin<br>Dose related effects on Wnt/b-catenin pathway	Limited ADME data in only cardiac cells.<br>No quantification of histology<br>Usually immunohistochemistry is very challenging to quantify robustly.<br>In TEM picture MNP is shown as a dark spot<br>Question is a MNP visible (electron dense?

Hou et al., 2021<br>#<br>ICR male mice<br>4-5 weeks old<br>no body weight given<br><br>https://doi.org/10.1016/j.jhazmat.2020.124028	Drinking water per group for 35 days<br>Control 100 µg/L; 1000 µg/L, 10 mg/L<br>Average daily MNP exposure for each mouse (calculated by authors): 0.6-0.7 µg/day, 6-7 µg/day; 60-70 µg/day<br>Estimated daily dose for each mouse: 0.017, 0.173, 1.729 mg/kg-day	5 µm PS MNP from Tianjin Bestra Chromatography Technology Development Center.<br>Particles were sonicated before administration.<br>Suspensions in distilled water.<br>No physical characterisation of MNPs was performed.<br>No chemical analysis (of MNP associated chemicals)	Biokinetics not studied	Biokinetics not studied	Blood, testes and epididymis, sperm quality<br>Oxidate proteins<br>Nrf-2 and HO-1 gene expression<br>In vitro studies (cell lines)	No body weight differences, no dose response relative testis weight differences.<br>In all MNP groups reduced rate of living sperm, perhaps more in 60-70 µg/day group.<br>Dose related sperm malformations<br>No quantification of testes histology (on 1 picture per exposure group<br>Dose related NFkB protein expression in testes	No ADME data<br>MNPs were not characterized.<br><br>[compare concentrations with Xie]

						(predominantly in highest group), dose related reduced mRNA expression Nrf2 Increased cytokine levels in tests (dose related)	

Park et al 2020 ICR mice 6 weeks old<br><br>https://doi.org/10.1016/j.toxlet.2020.01.008	Oral gavage for 90 days 0.125, 0.5 or 2 mg (given in 200ul/day): resulting in 3.75, 15, 60 mg/kg bw day)	Surface modified PE MNP 40-48 µm (to contain acid and hydroxyl groups) Sigma-Aldrich).<br>Suspensions in drinking water.<br>No physical characterisation of MNPs was performed.<br>No chemical analysis (of MNP associated chemicals)	TEM	Stomach and Spleen was analysed using TEM, some ”PE MNP-like materials” detected in mast cells of stomach but not in spleen.	Body weight<br>TEM in spleen and stomach<br>Pathological screen in several tissues<br>BW of pups, spleen from dams<br>Immunoglobulin quantification in blood	Incidental effects on blood parameters (no dose relationships).<br>Body weight effects not correctly analysed.<br>Only in some animals histological findings are reported, while the number of observation seem to increase with increase dose, no clear dose response trends<br>No results of TEM analysis of tissues is reported.<br>Some effects on lymphocyte	Some kinetic data on stomach and spleen, but not on reproductive organs (not sampled).<br><br>Some statistical results are reported with p<0.5 and p<0.1<br><br>In this summary or effects with p<0.05 are listed.<br>Why do authors discuss potential uptake via stomach?<br>No results from intestine reported<br><br>Surface modified MNPs!

						s in dams are reported (ration CD8/CD4 dose related decrease. BW of dams 4 days after birth lower, dose related	

Experimental Design		Particle Characterization	Biokinetics		Toxicodynamics		
**Study type and age of animals used**	**Exposure scheme**	**Microplastic type, and concentration**	**Presence of microplastic in (target) organ**	**MNP quantification method**	**Tissues, endpoint**	**results**	**comments**
Wei et al., 2020<br>6 week old 180 ± 20 g<br>DOI: 10.1002/tox.23095	Control; 0.5 5 and 50 mg/L in deionized water<br>Drinking water (not clear if individual or group administration)	510.4 nm (hydrodynamic size) pristine sizes not provided<br>PS MNP from Tianjin Baseline ChromTech research Center.<br>Physical characterisation of MNPs was performed.<br>No chemical analysis (of MNP associated chemicals)	Biokinetics not studied	Biokinetics not studied	Heart histology, proteins<br>Cytokines in plasma	Dose related effects on MDA, SOD, GSH and CAT in heart tissue.<br>At highest concentrations inflammatory factors increased<br>Dose related induction of NFkB pathway.	No ADME data

Experimental Design		Particle Characterization	Biokinetics		Toxicodynamics		
**Study type and age of animals used**	**Exposure scheme**	**Microplastic type, and concentration**	**Presence of microplastic in (target) organ**	**MNP quantification method**	**Tissues, endpoint**	**results**	**comments**
Stock et al., 2019<br>Male Hmox1 reporter mice on a C57BL/6NTac background<br>16-20 weeks<br>.<br>10.1007/s00204-019-02478-7	Oral gavage. Control (vehicle [0.5% (w/v) carboxymethyl cellulose) or 1 µm (4.55 x 10^7 particles; 4 µm (4.55 x 10^7 particles; 10 µm (1.49 x 10^7 particles in CMC 10mL/kg BW; 3 times per week for 28 days. Animals sacrificed 3 days after last exposure.	Carboxyalted fluorescent PS MNP; 1 µm carboxylated PS from Thermo Fischer Scientific, 4 (3.6-4.5) µm and 10 (10.0-14.0)µm both sulfate PS from Kisker Biotech GmbH. Physical characterisation of MNPs was performed. No chemical analysis (of MNP associated chemicals)	Median lob of liver, duodenum, ileum, jejunum, testes, large intestine, lung, heart, spleen, kidneys	Confocal miroscopy	Only some MNPs were observed in intestinal walls, due to limited number not quantified. No MNPs in liver, spleen and kidney (testes were not mentioned while tissue was sampled)	No histological changes were observed in intestinal samples, kidney, spleen and liver (testes not reported). Also no effects on the induction of β galatosidase induction) reporter (oxidative stress/ inflammation) was reported	No daily administration (only 3 times per week), 3 day wash out period. No quantification of histology performed. In vitro results are not summarized in this table. Negatively charged MNPs

**Experimental Design**		**Particle Characterization**	**Biokinetics**		**Toxicodynamics**		
**Study type and age of animals used**	**Exposure scheme**	**Microplastic type, and concentration**	**Presence of microplastic in (target) organ**	**MNP quantification method**	**Tissues, endpoint**	**results**	**comments**
Jin et al., 2021<br>6 weeks old BALB/C mice<br>https://doi.org/10.1016/j.jhazmat.2020.123430	Oral gavage of 0.5, 4 and 10 µm PS MNPs 100µl dd water with 10 mg/ml daily during 28 days Mice were also exposed to fluorescent MNPs of the same size	PS MNP (1.0% w/v, 10 ml) from Tianjin Baseline ChromTech research Center.<br>Physical characterisation of MNPs was performed.<br>No chemical analysis (of MNP associated chemicals)	Fluorescent intensity was observed in gastric tissues and (limitedly) in testis of 4 and 10 um MNP exposed groups	Gastric tissue and testis were excides and (ex vivo) imaged after the last exposure	Food intake and body weight was monitored<br>Testis histology and testosterone measurements<br>Sperm viability<br>Cytokine expression	Reduced food intake and bodyweight in 10 µm group.<br>Histology was not quantified<br>Serum testosterone levels were reduced in 10 µm group<br>Sperm viability was reduced 10 µm group<br>MNP exposure increased cytokine expression in testis	MNPs had a negative Zeta potential<br>Ex vivo fluorescence was not calibrated.<br>One concentration per size.<br><br>Dr. Katja Teerds - proper control group included, so experimental design for this experiment is OK, 1. group size of 3 is small and difficult to analyze

Experimental Design		Particle Characterization	Biokinetics		Toxicodynamics		
**Study type and age of animals used**	**Exposure scheme**	**Microplastic type, and concentration**	**Presence of microplastic in (target) organ**	**MNP quantification method**	**Tissues, endpoint**	**results**	**comments**
							statistically; Body weight, water intake, testis weight reliable - Fig. 3; Testis structure not reliable (qualtitative) - Fig. 4A; Testosterone reliable, however LH and FSH should also be analyzed- Fig. 4B; Sperm viability and abnormality somewhat reliable

**Experimental Design**		**Particle Characterization**	**Biokinetics**		**Toxicodynamics**		
**Study type and age of animals used**	**Exposure scheme**	**Microplastic type, and concentration**	**Presence of microplastic in (target) organ**	**MNP quantification method**	**Tissues, endpoint**	**results**	**comments**
							(unclear how many epididymi analyzed or total sperm) - Fig. 5; TNF-a, IL-6, MCP-1, CXCL10 somewhat reliable - Figure 6; Blood-testis barrier disruption not very reliable (qualitative) - Fig. 7

**Experimental Design**		**Particle Characterization**	**Biokinetics**		**Toxicodynamics**		
**Study type and age of animals used**	**Exposure scheme**	**Microplastic type, and concentration**	**Presence of microplastic in (target) organ**	**MNP quantification method**	**Tissues, endpoint**	**results**	**comments**
Li et al., 2021 C57 male mice 5 weeks old<br><br>https://doi.org/10.1016/j.jhazmat.2020.123933	20 mg/kg bw/day for 30 day via drinking water (housing in groups)	Green fluorescent 5 µm (2.5 % w/v) PS MNPs from Tianjin Baseline ChromTech research Center. Physical characterisation of MNPs was performed. No chemical analysis (of MNP associated chemicals)	Fluorescence's of liver slices was shown, but at different magnifications and not quantified nor caribrated. Also background signal in control group	Fluorescence	Inflammation and apoptosis in liver	No effects on ALT and AST, increased expression of cytokines	Only liver was studied in vivo

**Experimental Design**		**Particle Characterization**	**Biokinetics**		**Toxicodynamics**		
**Study type and age of animals used**	**Exposure scheme**	**Microplastic type, and concentration**	**Presence of microplastic in (target) organ**	**MNP quantification method**	**Tissues, endpoint**	**results**	**comments**
Luo et al., 2019<br>ICR mice<br>7 weeks old<br><br>http://dx.doi.org/10.1021/acs.est.9b03191	100, 1000 ug/L via drinking water (housed in breeding pairs)	5 μm PS spheres<br>Microspheres-Nanospheres (New York)<br>No chemical analysis of associated contaminants			Metabolic disorders in maternal mice (gut microbiota dysbiosis, gut barrier dysfunction). Metabolic changes in F1 and F2. Hepatic lipid accumulation in F1.		

Experimental Design		Particle Characterization	Biokinetics		Toxicodynamics		
**Study type and age of animals used**	**Exposure scheme**	**Microplastic type, and concentration**	**Presence of microplastic in (target) organ**	**MNP quantification method**	**Tissues, endpoint**	**results**	**comments**
Zheng et al (2021)<br>C57 mice<br>7 weeks old<br>https://doi.org/10.1016/j.scitotenv.2020.143085	500 ug/L in drinking water<br>28 day exposure	5 µm PS spheres<br>Tianjin BaseLine ChromTech Research Centre			Exaggerated inflammation and lipid homeostasis in mice with induced acute colitis		

**Experimental Design**		**Particle Characterization**	**Biokinetics**		**Toxicodynamics**		
**Study type and age of animals used**	**Exposure scheme**	**Microplastic type, and concentration**	**Presence of microplastic in (target) organ**	**MNP quantification method**	**Tissues, endpoint**	**results**	**comments**
Huang et al (2015) Pregnant FVB/N mice http://dx.doi.org/10.1016/j.placenta.2015.10.007	300 ug jugular vein injection	20 - 500 nm Yellow-green fluorescent PS spheres (carboxylate-modified)	Fluorescence microscopy and TEM demonstrate uptake into placenta, brain, lung and liver				

**Experimental Design**		**Particle Characterization**	**Biokinetics**		**Toxicodynamics**		
**Study type and age of animals used**	**Exposure scheme**	**Microplastic type, and concentration**	**Presence of microplastic in (target) organ**	**MNP quantification method**	**Tissues, endpoint**	**results**	**comments**
Han et al (2020) Six-day pregnant mice https://doi.org/10.1007/s43188-020-00086-7	0, 6, 60 ug/mouse in drinking water (housed in groups of 3 mice) and seven intratracheal administration during pregnancy and five after delivery	10 - 45 um red fluorescent polyethylene spheres Cospheric (Santa Barbara, CA)	Fluorescence microscopy. Tissue were homogenized		No significant alterations in number of offspring or body weight	No significant effects	

Experimental Design		Particle Characterization	Biokinetics		Toxicodynamics		
**Study type and age of animals used**	**Exposure scheme**	**Microplastic type, and concentration**	**Presence of microplastic in (target) organ**	**MNP quantification method**	**Tissues, endpoint**	**results**	**comments**
Li et al (2021) Six-week old (180 g) male Wistar rats https://link.springer.com/article/10.1007/s11356-021-13911-9	0, 0.015, 0.15, 1.5 mg/day via drinking water.<br><br>Final weight not reported. Estimated dose:<br>0.083, 0.83, 8.3 mg/kg-day<br>8 mice/group<br>90 day exposure	0.5 um polystyrene spheres<br>Tianjian Baseline Chromtech Research Centre (Tianjin, China)	Uptake not characterized		Only testis tissue examined.<br><br>Significant increase in sperm abnormality at 1.5 mg/d, and significant decrease in sperm concentration and motility at highest dose.<br><br>Histopathological changes observed in 0.15 mg/d and 1.5 mg/d groups, including shedding of spermatogenic cells and intraepithelial vacuolization.<br>Oxidative Stress: MDA upregulated in 0.15 and 1.5 mg/d. CAT, GSH-PS, and SOD decreased in 0.15 and 1.5 mg/d.<br><br>Apoptosis: Bax immunopositive products in cytoplasm of spermatogenic cells significantly increased and Bcl-2 decreased in 0.15 mg/d and 1.5 mg/d groups. Increased fluorescence via TUNEL staining in testis at 0.15 mg/d and 1.5 mg/d		Final body weight not determined<br>.

Experimental Design		Particle Characterization	Biokinetics		Toxicodynamics		
**Study type and age of animals used**	**Exposure scheme**	**Microplastic type, and concentration**	**Presence of microplastic in (target) organ**	**MNP quantification method**	**Tissues, endpoint**	**results**	**comments**
					doses.<br>P38 phosphorylation and Nrf2 increased in 0.15 and 1.5 mg/d groups.<br><br>Significant decreases in blood-brain barrier protein expression in 0.15 mg/d and 1.5 mg/d.		

Experimental Design		Particle Characterization	Biokinetics		Toxicodynamics		
**Study type and age of animals used**	**Exposure scheme**	**Microplastic type, and concentration**	**Presence of microplastic in (target) organ**	**MNP quantification method**	**Tissues, endpoint**	**results**	**comments**
Li et al (2021a)<br>C57 male mice (5 weeks old)<br>https://doi.org/10.1016/j.jhazmat.2020.123933	20 mg/kg-day in drinking water (RO water) (n = 6)<br>Sodium hydrosulfide intraperitoneal administration (75 umol/kg-day)<br>control; PS-exposure only; sodium hydrosulfide only; sodium hydrosulfide + PS exposure<br>NaHS used as protective agent for oxidative stress.<br>30 day exposure	Green fluorescent 5 um polystyrene spheres (TianJin Baseline Chromtech Research Center - China).	Uptake in liver confirmed	Transmission electron microscopy, fluorescence microscopy	Histology analysis of liver tissues<br>Serum alanine aminotransferase and aspartate amniotransferase analysis<br>Superoxide disumatase, catalase, reduced glutathione, malondialdehyde analysis<br>RT-qPCR analysis of pro-inflammatory cytokines<br>Nrf2, Keap1, HO-1, NQO1, and B-actin in liver via Western blot<br>ROS and apoptosis analysis with primary hepatocytes<br>Mitochondrial membrane potential detection in hepatocytes	Significant decrease in T-SOD, GSH and increase in MDA with microplastics exposure. NaHS attenuated effects.<br>'Severe' vacuolar degeneration, chronic inflammatory infiltration, hepatocellular edema. Attenuation of histopathological liver changes with NaHS.<br>Increased interleukin-1B, tumor necrosis	

Experimental Design		Particle Characterization	Biokinetics		Toxicodynamics		
**Study type and age of animals used**	**Exposure scheme**	**Microplastic type, and concentration**	**Presence of microplastic in (target) organ**	**MNP quantification method**	**Tissues, endpoint**	**results**	**comments**
						factor-a, monocyte chemotactic protein-1, G-X-G motif chemokine ligand 2, caspas01. NaHS partly attenuated inflammatory cytokine levels. PS exposure increased ROS levels in primary hepatocytes and cellular apoptosis. Both effects attenuated by NaHS. Nrf2 significantly increased in primary hepatocytes	

Experimental Design		Particle Characterization	Biokinetics		Toxicodynamics		
**Study type and age of animals used**	**Exposure scheme**	**Microplastic type, and concentration**	**Presence of microplastic in (target) organ**	**MNP quantification method**	**Tissues, endpoint**	**results**	**comments**
						(cytoplasm). NaHS increased Nrf2 protein levels and nuclear translocation. PS increased Keap1 protein levels, but attenuated by NaHS. Nrf2 knockdown in primary hepatocytes decreased oxidative damage by PS.	

Table S3. QA/QC scoring guidance for *in vivo* study criteria

[Note that this table is replicated from Gouin et al (2021).

Criteria	Suggested guidance for scoring studies
**A: Particle Characterization**	
1. Particle size	1. Reported, but limited to average size as obtained from a supplier
	2. Reported, with specific information on particle size variance
2. Particle shape	1. Reported, but limited to average shape as obtained from a supplier
	2. Reported, with verification provided using high resolution digital images
3. Polymer type	1. Reported, but limited to composition obtained from supplier
	2. Reported, with verification using FTIR, Raman or other applicable approach
4. Source of particles	1. Reported, but limited to name of supplier/manufacturer
	2. Reported, with specific details related to how the particles were produced
5. Particle surface chemistry	1. Reported, but limited to information obtained from supplier
	2. Reported, with verification of the particle surface chemistry properties, such as charge, hydrophobicity, etc. in the test medium
6. Chemical purity	1. Reported, but limited to information obtained from supplier. No steps taken to clean particles or to remove chemical impurities, such as surfactants, stabilisers, emulsifiers, etc.
	2. Reported, with verification and/or evidence to demonstrate that particles appropriately cleaned
7. Microbial contamination	1. Reported as potentially present/absent. No steps taken to verify or remove
	2. Reported, with verification of the presence or absence of endotoxin.
**B: Experimental design**	
1. Particle concentration units	1. Reported, but limited to a single metric, such as mass/volume or number/volume

Criteria	Suggested guidance for scoring studies
	2. Reported with details provided for both mass/volume and number/volume
2. Particle stability [a]	1. Studies that provide limited (qualitative/semi-quantitative) information supporting particle stability within the test medium as assessed by the evaluator.
	2. Studies that measure and verify particle stability within the test medium, inlcuding details of aggregation kinetics and/or high resolution digital images, or other method assessed as appropriate by the evaluator, such as characterization of zeta-potentials and behaviour in water versus medium.
3. Test medium and/or delivery vehicle	1. Partial inclusion of information, such as when more than one test condition is used.
	2. Test medium or vehicle used to dose particles is fully reported.
4. Applied dose/concentration	1. Nominal test doses or concentrations are reported in the exposure media, with dilution factors.
	2. Test doses or concentrations in the exposure media, with dilution factors are reported, which are verified analytically, i.e. represent the actual dose or concentration.
5. Homogeneity of exposure	1. Limited information pertaining to the homogeneity of the exposure dose is reported and which is defined as insufficient with explanation by the evaluator. MPs administered should ideally be representative of well-mixed or dispersed in solutions within the in vitro test system.
	2. Verification pertaining to the homogeneity of the exposure dosed. MPs administered are demonstrated to be well-mixed or dispersed within the in vitro test system. Characterization of polydispersity index (PDI) may represent a substitute to support an understanding that particles have a high homogeneity in the particle population (i.e. PDI <0.2 strengthens assumption of high homogeneity)
6. Description of fundamental in vitro model test system elements	1. Description must include, for example, type of cells or tissue used: primary cells, cell lines, reconstructed tissue, isolated (parts of) organs, bacteria, yeast cells. Assign a value of '1' if origin/source of test system not described, examples for such information are:<br>- laboratory/scientist providing cell lines,<br>- commercial provider of test systems,<br>- origin of ex vivo organs, tissues, primary cells etc.
	2. Description may include, for example, type of cells or tissue used: primary cells, cell lines, reconstructed tissue, isolated (parts of) organs, bacteria, yeast cells. A value of '2' assigned where description of source/origin of test system is included.

**Criteria**	**Suggested guidance for scoring studies**
7. Inclusion of additional test system parameters	1. Limited information providing additional important information: eg. Cell density used, sample volume, well surface, vehicle or solvent used; maximum concentration of solvent; explanation of specific conditions applying during exposure (static, dynamic, with light, darkness...); application of new medium on or after exposure; limited information describing how endpoints were determined (cytotoxicity, ROS generation, misfolded proteins, cytokines, etc)
	2. Exceptional details provided describing the method: eg. Cell density used, sample volume, well surface, vehicle or solvent used; maximum concentration of solvent; explanation of specific conditions applying during exposure (static, dynamic, with light, darkness...); application of new medium on or after exposure; Detailed information describing how endpoints were determined (cytotoxicity, ROS generation, misfolded proteins, cytokines, etc)
8. Sample size / Replicates (e.g. min 10^6 cells/well; 2-3 replicates)	1. Depending on the in vitro test system model used and experimental design the sample size is defined as insufficient by the evaluator with explanation.
	2. Satisfactory reporting of the sample size / replicates.
9. Frequency and duration of exposure	1. Insufficient information reported to fully evaluate the frequency and duration of the exposure, as well as time-points or concerns raised regarding the relevance of the duration of the exposure with evaluator explanation.
	2. Satisfactory reporting on the frequency and duration of the exposure, as well as time-points of observations. Time points of observations may not be mentioned when the experimental set-up makes clear that observation takes place immediately after end of exposure (considered sufficient). Please check also figures and tables for respective information.
10. Controls	1. Study includes a negative/vehicle control
	2. Study includes the reporting of a particle and/or positive control
**C. Applicability for risk assessment**	
1. Statistical analysis	1. Statistical methods should be suitable for the dataset under analysis and the goal of the analysis. In case statistical methods are provided but the evaluator is not able to judge the suitability of the statistical methods, this question should be scored by 1 with explanation.
	2. Statistical methods should be suitable for the dataset under analysis and the goal of the analysis.
2. Endpoints [b]	1. Endpoints insufficient to assess if endpoint relates to an adaptive or adverse effect in humans
	2. Endpoints sufficient to assess response as either adaptive or adverse effect in humans

**Criteria**	**Suggested guidance for scoring studies**
3. Dose-response relationship [c]	1. Dose-response relationships based on ≥3 exposure concentrations with a concentration range ≥ 2.5x, excluding the control.
	2. Dose-response relationships based on ≥3 exposure concentrations with a concentration range ≥2.5x, excluding the control. Is there a concentration (or other particle descriptor) dependent response? Score '2' if reasonable explanation given to interpret dose-response relationship.
4. Concentration range [d]	1. Unable to demonstrate environmentally relevant concentration range, supported with arguments.
	2. Concentration range is reported to be consistent with environmentally relevant concentrations.
5. Effect threshold	1. Effect thresholds reported as No observed effect concentration/no observed adverse effect level (NOEC/NOAEL), lowest observed effect concentration (LOEC), or when no error data are provided. Alternatively, if no thresholds reported, the study provides access to raw data to enable threshold values to be derived.
	2. Effect threshold concentrations, accompanied with estimates of error or uncertainty, and which reflect the L(E)Cx dervied from dose-response relationship modelling, with error data (95% confidence interval, standard error or standard deviation).
6. Test particle relevance[e]	1. The diversity of the particles is limited to assessing only one or two properties, such as looking at different particle sizes, shapes, surface charge or densities of the same polymer or comparing between two different polymers of same properties.
	2. Studies that use multiple types of particles and combinations of properties that reflect a variety of sizes, shapes, surface charge and densities in one mixture exposure.

Assign a score of zero (0) if:
[a] only nominal concentration reported.
[b] not reported or insufficient to assess if endpoint relates to adaptive or adverse effect.
[c] not reported or uses ≤2 exposure concentrations.
[d] concentration range much greater than the range of environmentally relevant concentrations.
[e] Not reported or only limited to a single type of particle <u>and</u> single property.

Table S4. Summary of Screening and Prioritization Criteria Scores for *in vivo* oral ingestion studies (minimal criteria noted with asterisks *)

Study	Particle Size*	Particle Shape*	Polymer Type*	Particle Source*	Surface Chemistry	Chemical Purity	Microbial Contamination	Concentration Units	Particle Stability	Test Vehicle*	Administered Dose*	Homogeneity of Exposure	Administration Route*	Test Species*	Feeding/Housing Conditions	Sample Size*	Frequency/Duration of Exposure*	Controls*	Replicates	Internal Dose Confirmation	Statistical Analysis	Endpoints*	Dose-Response*	Concentration Range	Effect Thresholds*	Test Particle Relevance
Amereh (2019)	2	1	1	1	2	0	0	1	2	2	1	1	2	2	2	2	2	1	2	0	2	1	1	2	1	0
Amereh (2020)	2	1	1	2	2	0	0	1	2	2	1	1	2	2	2	2	2	1	2	0	2	1	1	2	1	0
An (2021)	2	2	2	1	0	0	0	1	2	1	1	1	1	2	1	1	2	1	1	1	2	1	2	2	1	0
daCostaAraujo (2020)	2	2	1	1	0	0	0	2	1	1	1	0	1	1	1	1	1	1	2	1	2	2	0	2	0	1
Deng (2017)	2	2	2	1	0	0	0	2	1	2	1	0	2	2	2	1	2	1	2	1	2	1	1	2	1	1
Deng (2018)	2	2	2	1	0	0	0	2	0	1	1	0	1	2	2	2	1	1	2	1	2	2	0	1	0	1

Deng (2020)	2	2	2	1	0	2	0	2	0	1	1	1	1	1	2	1	2	1	2	1	2	1	0	2	0	1
Hou (2021a)	1	1	1	1	0	0	0	1	1	1	1	1	1	2	2	2	2	1	2	0	2	2	1	0	1	0
Hou (2021b)	2	2	2	1	0	0	0	1	0	2	1	2	1	2	2	2	1	1	0	0	2	1	1	0	1	0
Jiang (2021)	1	1	1	1	0	0	0	1	0	2	1	0	1	2	2	1	2	1	1	0	2	1	0	1	0	0
Jin (2019)	1	1	1	1	0	0	0	2	0	1	1	1	1	1	2	2	1	1	2	1	2	1	0	0	0	0
Jin (2020)	2	2	2	1	1	0	0	1	0	2	1	1	1	1	1	1	2	1	1	1	2	2	0	0	0	1
Li (2021b)	2	2	2	1	0	0	0	2	1	2	1	2	1	1	2	2	1	1	2	0	2	2	2	2	1	1
Li (2020a)	1	1	1	1	0	0	0	1	0	1	1	0	1	1	0	2	2	1	2	0	2	1	1	1	1	1
Li (2020b)	2	2	2	1	0	0	0	2	1	1	1	0	1	1	1	1	2	1	2	1	2	1	1	0	1	0
Li (2021a)	2	2	1	1	0	0	0	1	0	1	1	0	1	1	0	1	2	1	1	1	2	1	0	0	0	0
Lu (2018)	1	2	1	1	0	0	0	2	0	1	1	0	1	1	2	2	2	1	2	0	2	2	1	0	0	1
Luo (2019a)	1	1	1	1	0	0	0	1	0	1	1	1	1	2	2	1	1	1	1	0	2	2	1	1	0	1

Luo (2019b)	1	1	1	1	0	0	0	1	1	1	1	0	1	2	1	0	1	1	2	0	1	2	1	1	0	0
Luo (2019b)	1	1	1	1	0	0	0	1	1	1	1	0	1	2	1	0	1	1	2	0	1	2	1	1	0	0
Luo (2019b)	1	1	1	1	0	0	0	1	1	1	1	0	1	2	1	0	1	1	2	0	1	2	1	1	0	0
Molugu (2006)	1	1	1	1	0	0	1	1	0	1	1	0	1	1	0	1	1	1	1	0	2	2	0	0	0	1
Park (2020)	2	2	1	1	1	0	0	2	0	1	1	1	2	2	1	2	2	1	2	1	1	2	1	0	1	0
Park (2020)	2	2	1	1	1	0	0	2	0	1	1	1	2	2	1	2	2	1	2	1	1	2	1	0	1	0
Rafiee (2018)	2	1	1	2	2	0	0	0	0	2	1	1	2	2	2	1	2	1	1	0	2	2	1	0	0	0
Shengchen (2021)	2	2	2	1	0	0	0	1	0	2	1	0	2	2	1	2	2	1	2	0	2	2	0	0	0	2
Stock (2019)	2	2	1	1	1	0	0	1	0	2	1	0	1	2	2	1	2	1	2	1	2	1	0	1	0	1
Sun (2021)	2	2	1	1	0	1	0	2	0	2	1	0	1	2	2	1	2	1	1	1	2	1	0	1	1	0
Sun (2021)	2	2	2	1	0	0	0	2	0	2	1	1	1	2	2	1	2	1	2	0	2	1	0	1	1	0

Wang (2021)	2	2	1	1	2	0	0	2	0	2	1	0	2	2	2	1	2	1	1	2	2	1	0	1	1	0
Wei (2020)	2	2	2	1	1	0	0	1	1	1	1	0	1	2	1	1	2	1	2	0	2	1	1	0	1	0
Xie (2020)	1	1	1	1	0	0	0	1	1	1	1	1	1	2	2	2	2	1	2	0	2	2	1	0	1	0
Xu (2021)	2	2	2	1	2	0	0	1	0	2	1	0	2	2	1	1	2	1	1	1	2	2	0	0	0	2
Zheng (2021)	1	2	2	1	0	1	0	1	0	1	1	0	1	1	1	2	2	1	2	0	1	2	0	0	0	0

Table S5. Summary of Outside Expert Reviews

Study	Dr. Jodi Flaws (U of Illinois)	Ruben Gandia (Health Canada)	Dr. Katja Teerds (Wageningen)	Dr. Leon Earl Gray (USEPA)	Dr. Jonathan Powell (U of Cambridge)	Dr. Aimen Farraj (USEPA)	Dr. Pauliina Damdimopolou (Karolinska Institutet)
Wei et al (2021)						X	
Li et al (2020)			X				
Li et al (2021)	X						
Hou et al (2021a)	X	X	X	X			
Hou et al (2021b)	X		X				X
Xie et al. (2020)	X	X	X	X			
Amereh et al (2019)			X	X			
An et al (2021)	X		X				X
Amereh et al (2020)	X						
Jin et al (2021)			X				
Deng et al (2020)					X		
Deng et al (2017)					X		

doi	Author (year)	General Endpoint Category	More specific endpoint category	Most specific endpoint category	Reliability (NA = unrated)	Relevance to human health (NA = not relevant or unrated)
10.1039/c9tx00147f	Amereh (2019)	Stress	General Stress	Glutamicoxaloaceticttransminase Concentration	NA	NA
		Stress	General Stress	Glutamicpyruvictransaminase Concentration	NA	NA
		Endocrine Signaling	Thyroid	Free T3 Concentration	unreliable	NA
		Endocrine Signaling	Thyroid	Free T4 Concentration	unreliable	NA
		Endocrine Signaling	Thyroid	T3/T4 Ratio	unreliable	NA
		Endocrine Signaling	Thyroid	Free T3/Free T4 Ratio	unreliable	NA
		Endocrine Signaling	Thyroid	TSH Concentration	unreliable	NA
		Metabolism	Lipid Metabolism	Cholesterol Concentration	reliable	NA
		Metabolism	Lipid Metabolism	HDL Concentration	NA	NA
		Metabolism	Lipid Metabolism	Low Density Lipoprotein Concentration	NA	NA
10.1016/j.envpol.2020.114158	Amereh (2020)	Endocrine Signaling	Reproductive Endocrine Signaling	Testosterone Concentration	reliable	relevant
		Endocrine Signaling	Reproductive Endocrine Signaling	Luteinizing hormone Concentration	reliable	relevant
		Endocrine Signaling	Reproductive Endocrine Signaling	Follicle Stimulating Hormone Concentration	reliable	relevant
		Fitness	Reproduction	Sperm Motility	reliable	relevant

doi	Author (year)	General Endpoint Category	More specific endpoint category	Most specific endpoint category	Reliability (NA = unrated)	Relevance to human health (NA = not relevant or unrated)
		Fitness	Reproduction	Sperm Viability	reliable	relevant
		Fitness	Reproduction	Sperm Maturity	reliable	relevant
		Fitness	Reproduction	Sperm Deformity	reliable	relevant
		Fitness	Reproduction	Sperm DNA damage	reliable	relevant
		Fitness	Reproduction	plzf mRNA expression	reliable	NA
		Fitness	Reproduction	dazl mRNA expression	reliable	NA
		Fitness	Reproduction	abp mRNA expression	reliable	NA
		Endocrine Signaling	Reproductive Endocrine Signaling	Follicle Stimulating Hormone mRNA expression	unreliable	relevant
		Fitness	Reproduction	Sperm Count	reliable	relevant
		Fitness	Reproduction	Seminiferous Tubules Diameter	reliable	relevant
		Fitness	Reproduction	Germinal Epithelium Cell Height	reliable	relevant
		Fitness	Reproduction	Testicular Capsule Diameter	reliable	relevant
10.1016/j.tox.2020.152665	An (2021)	Endocrine Signaling	Reproductive Endocrine Signaling	Anti Mullerian Hormone Concentration	reliable	relevant
		Fitness	Reproduction	Number of Growing Follicles	unreliable	relevant
		Metabolism	Oxidative Stress	Malondialdehyde Concentration	unreliable	NA
		Metabolism	Oxidative Stress	Catalase Activity	unreliable	NA
		Metabolism	Oxidative Stress	Glutathione Concentration	unreliable	NA
		Metabolism	Oxidative Stress	SOD Activity	unreliable	NA
		Metabolism	Oxidative Stress	ROS Production	unreliable	NA
		Fitness	Reproduction	Collagenous Fiber Staining	unreliable	NA
		Fitness	Reproduction	Fibronectin protein expression (Reproductive Tissue)	unreliable	NA
		Cell Growth and Proliferation	Apoptosis and Cell Cycle	BAX protein expression	unreliable	NA

doi	Author (year)	General Endpoint Category	More specific endpoint category	Most specific endpoint category	Reliability (NA = unrated)	Relevance to human health (NA = not relevant or unrated)
		Cell Growth and Proliferation	Apoptosis and Cell Cycle	BCL2 Protein Expression	unreliable	NA
		Cell Growth and Proliferation	Apoptosis and Cell Cycle	wnt protein expression	unreliable	NA
		Cell Growth and Proliferation	Apoptosis and Cell Cycle	tbfb protein expression	unreliable	NA
		Fitness	Reproduction	Beta Catenin Protein Expression (Reproductive Tissue)	unreliable	NA
		Fitness	Reproduction	Phosphorylated Beta Catenin Protein Expression (Reproductive Tissue)	unreliable	NA
		Fitness	Reproduction	Alpha Smooth Muscle Actin Protein Expression (Reproductive Tissue)	unreliable	NA
		Fitness	Reproduction	Callogen Protein Expression	unreliable	NA
		Fitness	Reproduction	Callogen 3 Protein Expression	unreliable	NA
10.1016/j.jhazmat.2020.123263	daCostaAraujo (2020)	Behavioral, Sensory, Neurological	Exploration	Distance Traveled (Open Field Test)	NA	NA
		Behavioral, Sensory, Neurological	Locomotion	Locomotion Speed (Open Field Test)	NA	NA
		Behavioral, Sensory, Neurological	Locomotion	Anxiety Index (Open Field Test)	NA	NA

doi	Author (year)	General Endpoint Category	More specific endpoint category	Most specific endpoint category	Reliability (NA = unrated)	Relevance to human health (NA = not relevant or unrated)
		Behavioral, Sensory, Neurological	Anxiety	Shaking Frequency	NA	NA
		Behavioral, Sensory, Neurological	Predator Avoidance	Within Predator Field of View	NA	NA
10.1038/srep46687	Deng (2017)	Fitness	Body Condition	Body Weight	reliable	relevant
10.1016/j.jhazmat.2018.06.017	Deng (2018)	Behavioral, Sensory, Neurological	Nervous System	AchE Activity	NA	NA
		Metabolism	Oxidative Stress	Catalase Activity	NA	NA
		Metabolism	Energy Metabolism	Lactate Dehydrogenase Activity	NA	NA
10.1038/srep46687	Deng (2017)	Behavioral, Sensory, Neurological	Nervous System	AchE Activity	reliable	NA
		Metabolism	Energy Metabolism	ATP Concentration	reliable	NA
		Metabolism	Oxidative Stress	Catalase Activity	reliable	NA
		Metabolism	Lipid Metabolism	Cholesterol Concentration	reliable	NA
		Metabolism	Oxidative Stress	Glutathione Peroxidase Activity	reliable	NA
		Metabolism	Energy Metabolism	Lactate Dehydrogenase Activity	reliable	NA
		Metabolism	Oxidative Stress	SOD Activity	reliable	NA
		Metabolism	Lipid Metabolism	Triglyceride Concentration	reliable	NA
10.1016/j.envint.2020.105916	Deng (2020)	Fitness	Body Condition	Body Weight	NA	NA
		Alimentary, Excretory	Intestinal Permeability	D Lactate Concentration	NA	NA
		Alimentary, Excretory	Intestinal Permeability	DAO Activity	NA	NA

doi	Author (year)	General Endpoint Category	More specific endpoint category	Most specific endpoint category	Reliability (NA = unrated)	Relevance to human health (NA = not relevant or unrated)
		Alimentary, Excretory	Digestive Tract Histological Abnormalities	Inflammatory Cell Infiltration	NA	NA
10.1038/srep46687	Deng (2017)	Alimentary, Excretory	Liver Histological Abnormalities	Liver Tissue Inflammation	reliable	relevant
		Alimentary, Excretory	Liver Histological Abnormalities	Lipid Droplets	reliable	relevant
10.1016/j.chemosphere.2020.129476	Estrela (2021)	Stress	DNA Damage	NA	NA	NA
		Metabolism	Oxidative Stress	NA	NA	NA
		Behavioral, Sensory, Neurological	Nervous System	NA	NA	NA
		Behavioral, Sensory, Neurological	Nervous System	AchE Activity	NA	NA
		Metabolism	Lipid Metabolism	Triglyceride Concentration	NA	NA
		Metabolism	Lipid Metabolism	NA	NA	NA
		Metabolism	Lipid Metabolism	Cholesterol Concentration	NA	NA
		Behavioral, Sensory, Neurological	NA	NA	NA	NA
10.1016/j.jhazmat.2020.124028	Hou (2021a)	Fitness	Body Condition	Testis Weight	unreliable	relevant
		Fitness	Reproduction	Sperm Deformity	reliable	relevant
		Endocrine Signaling	Reproductive Endocrine Signaling	Anti Mullerian Hormone Concentration	reliable	relevant
		Fitness	Reproduction	Testis Somatic Index	unreliable	relevant
		Fitness	Reproduction	Sperm Count	unreliable	relevant
		Fitness	Reproduction	Testis Histology	unreliable	relevant
		Immune	Inflammation	ikba protein expression	reliable	NA

doi	Author (year)	General Endpoint Category	More specific endpoint category	Most specific endpoint category	Reliability (NA = unrated)	Relevance to human health (NA = not relevant or unrated)
		Immune	Inflammation	nfkbp75 protein expression	reliable	NA
		Immune	Inflammation	p-nfkbp75 protein expression	reliable	NA
		Immune	Inflammation	IL1b protein expression	reliable	NA
		Immune	Inflammation	IL6 protein expression	reliable	NA
		Immune	Inflammation	tnfa protein expression	reliable	NA
		Immune	Inflammation	nrf2 protein expression	reliable	NA
		Immune	Inflammation	nrf2 mRNA expression	reliable	NA
		Stress	General Stress	ho1 protein expression	reliable	NA
		Cell Growth and Proliferation	Apoptosis and Cell Cycle	BAX/BCL2 protein expression	reliable	NA
		Metabolism	Oxidative Stress	NA	reliable	NA
		Metabolism	Oxidative Stress	Malondialdehyde Concentration	reliable	NA
		Metabolism	Oxidative Stress	Catalase Activity	reliable	NA
		Metabolism	Oxidative Stress	SOD Activity	reliable	NA
		Immune	Inflammation	IL18 Concentration	reliable	NA
		Immune	Inflammation	IL1b Concentration	reliable	NA
		Cell Growth and Proliferation	Apoptosis and Cell Cycle	TUNEL staining	reliable	NA
		Cell Growth and Proliferation	Apoptosis and Cell Cycle	Cleaved Caspase 3 protein expression	reliable	NA
		Immune	Inflammation	nlrp3 protein expression	reliable	NA
		Immune	Inflammation	p-nfkB protein expression	reliable	NA
		Immune	Inflammation	IL1b protein expression	reliable	NA
		Immune	Inflammation	Cleaved Gasdermin D protein expression	reliable	NA
		Immune	Inflammation	IL18 protein expression	reliable	NA

doi	Author (year)	General Endpoint Category	More specific endpoint category	Most specific endpoint category	Reliability (NA = unrated)	Relevance to human health (NA = not relevant or unrated)
		Immune	Inflammation	Cleaved Caspase 1 protein expression	reliable	NA
		Immune	Inflammation	ASC protein expression	reliable	NA
		Fitness	Reproduction	Number of Growing Follicles	unreliable	relevant
		Immune	Inflammation	Cleaved Caspase 1 protein expression	unreliable	NA
		Immune	Inflammation	nlrp3 protein expression	unreliable	NA
10.1016/j.ecoenv.2021.112345	Jiang (2021)	Metabolism	Lipid Metabolism	Triglyceride Concentration	NA	NA
		Alimentary, Excretory	Liver & Kidney Products	Aspartate Transaminase Concentration	NA	NA
		Immune	Inflammation	Interferon y Concentration	NA	NA
		Immune	Inflammation	NA	NA	NA
		Alimentary, Excretory	Bile Acid	Bile Acid Concentration	NA	NA
		Alimentary, Excretory	Bile Acid	cyp7a1 mRNA expression	NA	NA
		Alimentary, Excretory	Bile Acid	cyp8b1 mRNA expression	NA	NA
		Alimentary, Excretory	Bile Acid	cyp27a1 mRNA expression	NA	NA
		Alimentary, Excretory	Bile Acid	fxr mRNA expression	NA	NA
		Alimentary, Excretory	Bile Acid	mrp3 mRNA expression	NA	NA
		Alimentary, Excretory	Bile Acid	mrp2 mRNA expression	NA	NA
		Alimentary, Excretory	Bile Acid	NA	NA	NA
		Alimentary, Excretory	Bile Acid	ntcp mRNA expression	NA	NA

doi	Author (year)	General Endpoint Category	More specific endpoint category	Most specific endpoint category	Reliability (NA = unrated)	Relevance to human health (NA = not relevant or unrated)
		Microbiome	Diversity	Shannon Index	NA	NA
		Alimentary, Excretory	Liver Histological Abnormalities	Liver Tissue Inflammation	NA	relevant
10.1016/j.scitotenv.2018.08.353	Jin (2019)	Alimentary, Excretory	Bile Acid	Bile Acid Concentration	NA	NA
		Alimentary, Excretory	Bile Acid	abcb11 mRNA expression	NA	NA
		Alimentary, Excretory	Bile Acid	cyp27a1 mRNA expression	NA	NA
		Alimentary, Excretory	Intestinal Mucus Secretion	Gut Muscus Secretion	NA	NA
		Alimentary, Excretory	Intestinal Mucus Secretion	muc1 mRNA expression	NA	NA
		Metabolism	Amino Acid Metabolism	Arginine Concentration	NA	NA
		Alimentary, Excretory	Intestinal Mucus Secretion	muc2 mRNA expression	NA	NA
		Alimentary, Excretory	Intestinal Mucus Secretion	klf4 mRNA expression	NA	NA
		Alimentary, Excretory	Intestinal Mucus Secretion	retnlb mRNA expression	NA	NA
		Alimentary, Excretory	Intestinal Ion Transport	cftr mRNA expression	NA	NA
		Alimentary, Excretory	Intestinal Ion Transport	slc26a6 mRNA expression	NA	NA
		Alimentary, Excretory	Intestinal Ion Transport	nkcc1 mRNA expression	NA	NA
		Alimentary, Excretory	Intestinal Ion Transport	nhe3 mRNA expression	NA	NA

doi	Author (year)	General Endpoint Category	More specific endpoint category	Most specific endpoint category	Reliability (NA = unrated)	Relevance to human health (NA = not relevant or unrated)
		Alimentary, Excretory	Intestinal Ion Transport	cftr protein expression	NA	NA
		Alimentary, Excretory	Intestinal Ion Transport	nkcc1 protein expression	NA	NA
		Alimentary, Excretory	Intestinal Ion Transport	ano1 mRNA expression	NA	NA
		Microbiome	Firmicutes	Firmicutes Genomic DNA	NA	NA
		Microbiome	Protebacteria	a Proteobacteria Genomic DNA	NA	NA
		Microbiome	Protebacteria	B Proteobacteria Genomic DNA	NA	NA
		Microbiome	Protebacteria	y Proteobacteria Genomic DNA	NA	NA
		Metabolism	Amino Acid Metabolism	Fumarylacetoacetatehydrolase Concentration	NA	NA
		Metabolism	Lipid Metabolism	Triglyceride Concentration	NA	NA
10.1016/j.jhazmat.2020.123430	Jin (2020)	Endocrine Signaling	Reproductive Endocrine Signaling	Testosterone Concentration	NA	NA
		Fitness	Reproduction	Sperm Viability	NA	NA
		Fitness	Reproduction	Sperm Deformity	NA	NA
		Immune	Inflammation	tnfa mRNA expression	NA	NA
		Immune	Inflammation	IL6 mRNA expression	NA	NA
		Immune	Inflammation	mcp1 mRNA expression	NA	NA
		Immune	Inflammation	cxc10 mRNA expression	NA	NA
		Immune	Inflammation	TNFa Concentration	NA	NA
		Immune	Inflammation	Interleukin 6 Concentration	NA	NA
		Immune	Inflammation	mcp1 Concentration	NA	NA
		Immune	Inflammation	cxc10 concentration	NA	NA
		Fitness	Reproduction	Blood Testes Barrier Disruption	NA	NA
		Fitness	Reproduction	zo1 protein expression	NA	NA
		Fitness	Reproduction	Occludin protein expression	NA	NA

doi	Author (year)	General Endpoint Category	More specific endpoint category	Most specific endpoint category	Reliability (NA = unrated)	Relevance to human health (NA = not relevant or unrated)
		Fitness	Reproduction	N-cadherin protein expression	NA	NA
		Fitness	Reproduction	Beta Catenin Protein Expression (Reproductive Tissue)	NA	NA
		Fitness	Reproduction	Focal Adhesion Kinase protein expression	NA	NA
		Behavioral, Sensory, Neurological	Feeding	Food Intake	NA	NA
		Fitness	Body Condition	Body Weight	NA	NA
		Fitness	Reproduction	Testis Histology	NA	NA
10.1007/s11356-021-13911-9	Li (2021b)	Fitness	Reproduction	Sperm Deformity	reliable	relevant
10.1016/j.chemosphere.2019.125492	Li (2020a)	Immune	Immune Other	gcsf mRNA expression	NA	NA
		Immune	Inflammation	IL10 Release	NA	NA
		Immune	Inflammation	IL1a Release	unreliable	NA
		Immune	Immune Other	IL2 Release	NA	NA
		Immune	Immune Other	IL5 Release	NA	NA
		Immune	Inflammation	IL6 Release	NA	NA
		Immune	Immune Other	IL9 Release	NA	NA
		Alimentary, Excretory	Intestinal Inflammation	ap1 protein expression	NA	NA
		Immune	Inflammation	RANTES Release	NA	NA
		Immune	Immune Cells	TH17 Cell Count	unreliable	NA
		Microbiome	Diversity	Chao Diversity Index	unreliable	NA
		Microbiome	Diversity	Shannon Index	unreliable	NA
		Microbiome	Diversity	Unifrac Diversity	unreliable	NA
		Microbiome	Firmicutes	Lactobacillus Genomic DNA	NA	NA
		Microbiome	Firmicutes	Staphylococcus Genomic DNA	NA	NA
		Microbiome	Firmicutes	dubosiella Genomic DNA	NA	NA

doi	Author (year)	General Endpoint Category	More specific endpoint category	Most specific endpoint category	Reliability (NA = unrated)	Relevance to human health (NA = not relevant or unrated)
		Microbiome	Bacteriodetes	Bacteriodetes Genomic DNA	NA	NA
		Microbiome	Firmicutes	Blautia Genomic DNA	NA	NA
		Microbiome	Bacteriodetes	Parabacteroides Genomic DNA	NA	NA
		Microbiome	Melainabacteria	Melainabacteria Genomic DNA	NA	NA
		Microbiome	Bacteriodetes	Muribaculum Genomic DNA	NA	NA
		Microbiome	Protebacteria	Desulfovibrio Genomic DNA	NA	NA
		Microbiome	Firmicutes	Clostridiales Genomic DNA	NA	NA
		Microbiome	Verrucomicrobiae	Akkermansia Genomic DNA	NA	NA
		Immune	Immune Cells	Treg Count	unreliable	NA
		Alimentary, Excretory	Intestinal Inflammation	tlr4 protein expression	NA	NA
		Alimentary, Excretory	Intestinal Inflammation	irf5 protein expression	NA	NA
		Alimentary, Excretory	Intestinal Inflammation	Intestinal Tissue Inflammation	NA	relevant
10.1016/j.envpol.2020.115025	Li (2020b)	Circulatory	Heart Tissue	Heart Histology	NA	relevant
		Metabolism	Oxidative Stress	Malondialdehyde Concentration	NA	NA
		Metabolism	Oxidative Stress	Catalase Activity	NA	NA
		Metabolism	Oxidative Stress	Glutathione Peroxidase Activity	NA	NA
		Metabolism	Oxidative Stress	SOD Activity	NA	NA
		Circulatory	Heart Tissue	Collagenous Fiber Staining	NA	NA
		Circulatory	Heart Tissue	Fibronectin protein expression (Cardiovascular Tissue)	NA	NA
		Cell Growth and Proliferation	Apoptosis and Cell Cycle	BAX protein expression	NA	NA
		Cell Growth and Proliferation	Apoptosis and Cell Cycle	BCL2 Protein Expression	NA	NA
		Circulatory	Heart Tissue	Troponin 1 Concentration	NA	NA

doi	Author (year)	General Endpoint Category	More specific endpoint category	Most specific endpoint category	Reliability (NA = unrated)	Relevance to human health (NA = not relevant or unrated)
		Circulatory	Heart Tissue	Creatine Kinase Myocardial Band	NA	NA
		Cell Growth and Proliferation	Apoptosis and Cell Cycle	wnt protein expression	NA	NA
		Cell Growth and Proliferation	Apoptosis and Cell Cycle	tbfb protein expression	NA	NA
		Circulatory	Heart Tissue	Beta Catenin Protein Expression (Cardiovascular Tissue)	NA	NA
		Circulatory	Heart Tissue	Phosphorylated Beta Catenin Protein Expression (Cardiovascular Tissue)	NA	NA
		Circulatory	Heart Tissue	Alpha Smooth Muscle Actin Protein Expression (Cardiovascular Tissue)	NA	NA
		Circulatory	Heart Tissue	Callogen Protein Expression	NA	NA
		Circulatory	Heart Tissue	Callogen 3 Protein Expression	NA	NA
		Fitness	Reproduction	Sperm Count	unreliable	relevant
		Fitness	Reproduction	Sperm Motility	unreliable	relevant
		Fitness	Reproduction	Testis Histology	unreliable	relevant
		Metabolism	Oxidative Stress	Catalase Activity	reliable	NA
		Metabolism	Oxidative Stress	Glutathione Peroxidase Activity	reliable	NA
		Metabolism	Oxidative Stress	Malondialdehyde Concentration	reliable	NA
		Metabolism	Oxidative Stress	SOD Activity	reliable	NA
		Cell Growth and Proliferation	Apoptosis and Cell Cycle	BAX protein expression	unreliable	NA

doi	Author (year)	General Endpoint Category	More specific endpoint category	Most specific endpoint category	Reliability (NA = unrated)	Relevance to human health (NA = not relevant or unrated)
		Cell Growth and Proliferation	Apoptosis and Cell Cycle	BCL2 Protein Expression	unreliable	NA
		Stress	General Stress	p-p38 protein expression	reliable	NA
		Immune	Inflammation	nrf2 protein expression	reliable	NA
		Fitness	Reproduction	Occludin protein expression	reliable	NA
		Fitness	Reproduction	Connexin 43 protein expression	reliable	NA
		Fitness	Reproduction	N-cadherin protein expression	reliable	NA
		Fitness	Reproduction	claudin11 protein expression	reliable	NA
		Cell Growth and Proliferation	Apoptosis and Cell Cycle	TUNEL staining	reliable	NA
10.1016/j.jhazmat.2020.123933	Li (2021a)	Alimentary, Excretory	Liver Histological Abnormalities	Suzuki Score	NA	NA
		Immune	Inflammation	IL1b mRNA expression	NA	NA
		Immune	Inflammation	tnfa mRNA expression	NA	NA
		Immune	Inflammation	mcp1 mRNA expression	NA	NA
		Immune	Inflammation	cxcl2 mRNA expression	NA	NA
		Cell Growth and Proliferation	Apoptosis and Cell Cycle	Caspase 1 mRNA expression	NA	NA
		Immune	Inflammation	nrf2 protein expression	NA	NA
		Metabolism	Oxidative Stress	keap1 protein expression	NA	NA
		Metabolism	Oxidative Stress	SOD Activity	NA	NA
		Metabolism	Oxidative Stress	Glutathione Peroxidase Activity	NA	NA
		Metabolism	Oxidative Stress	Malondialdehyde Concentration	NA	NA
10.1016/j.scitotenv.2018.03.051	Lu (2018)	Metabolism	Lipid Metabolism	acc mRNA expression	NA	NA
		Metabolism	Lipid Metabolism	acl mRNA expression	NA	NA
		Alimentary, Excretory	Intestinal Mucus Secretion	Gut Muscus Secretion	NA	NA

doi	Author (year)	General Endpoint Category	More specific endpoint category	Most specific endpoint category	Reliability (NA = unrated)	Relevance to human health (NA = not relevant or unrated)
		Alimentary, Excretory	Intestinal Mucus Secretion	klf4 mRNA expression	NA	NA
		Alimentary, Excretory	Intestinal Mucus Secretion	muc1 mRNA expression	NA	NA
		Metabolism	Lipid Metabolism	Cholesterol Concentration	NA	NA
		Metabolism	Carb Metabolism	chrebp mRNA expression	NA	NA
		Fitness	Body Condition	Body Weight	NA	NA
		Fitness	Body Condition	Liver Index	NA	NA
		Fitness	Body Condition	Fat Index	NA	NA
		Microbiome	Firmicutes	Firmicutes Genomic DNA	NA	NA
		Microbiome	Bacteriodetes	Bacteriodetes Genomic DNA	NA	NA
		Microbiome	Actinobacteria	Actinobacteria Genomic DNA	NA	NA
		Microbiome	Protebacteria	a Proteobacteria Genomic DNA	NA	NA
		Metabolism	Carb Metabolism	cs mRNA expression	NA	NA
		Metabolism	Lipid Metabolism	dgat1 mRNA expression	NA	NA
		Metabolism	Lipid Metabolism	dgat2 mRNA expression	NA	NA
		Metabolism	Lipid Metabolism	fas mRNA expression	NA	NA
		Metabolism	Lipid Metabolism	fat mRNA expression	NA	NA
		Metabolism	Lipid Metabolism	fatp2 mRNA expression	NA	NA
		Metabolism	Lipid Metabolism	gpat mRNA expression	NA	NA
		Metabolism	Carb Metabolism	pk mRNA expression	NA	NA
		Metabolism	Lipid Metabolism	ppar a mRNA expression	NA	NA
		Metabolism	Lipid Metabolism	ppar y mRNA expression	NA	NA
		Metabolism	Carb Metabolism	Pyruvate Concentration	NA	NA
		Metabolism	Lipid Metabolism	Triglyceride Concentration	NA	NA
10.1016/j.envpol.2019.113122	Luo (2019a)	Metabolism	Lipid Metabolism	3 Hydroxyhexadecanoylcarnitine Concentration	NA	NA

doi	Author (year)	General Endpoint Category	More specific endpoint category	Most specific endpoint category	Reliability (NA = unrated)	Relevance to human health (NA = not relevant or unrated)
		Metabolism	Lipid Metabolism	3 Hydroxyhexadecenoylcarnitine Concentration	NA	NA
		Metabolism	Lipid Metabolism	Adipylcarnite Concentration	NA	NA
		Metabolism	Amino Acid Metabolism	Alanine Concentration	NA	NA
		Metabolism	Lipid Metabolism	C0/C16/C18 Ratio	NA	NA
		Metabolism	Lipid Metabolism	Cholesterol Concentration	NA	NA
		Metabolism	Amino Acid Metabolism	Citrulline Concentration	NA	NA
		Metabolism	Lipid Metabolism	fat mRNA expression	NA	NA
		Metabolism	Lipid Metabolism	fatp2 mRNA expression	NA	NA
		Metabolism	Lipid Metabolism	Free Carnitine Concentration	NA	NA
		Metabolism	Lipid Metabolism	Hexadecenoylcarnitine Concentration	NA	NA
		Metabolism	Lipid Metabolism	HDL Concentration	NA	NA
		Metabolism	Amino Acid Metabolism	Leucine Concentration	NA	NA
		Metabolism	Lipid Metabolism	Linoleylcarnitine Concentration	NA	NA
		Metabolism	Lipid Metabolism	Low Density Lipoprotein Concentration	NA	NA
		Metabolism	Amino Acid Metabolism	Methionine Concentration	NA	NA
		Metabolism	Lipid Metabolism	Nonestrified Fatty Acid Concentration	NA	NA
		Metabolism	Lipid Metabolism	Palmitoylcarnitine Concentration	NA	NA
		Metabolism	Lipid Metabolism	scd1 mRNA expression	NA	NA
		Metabolism	Lipid Metabolism	Triglyceride Concentration	NA	NA
		Metabolism	Amino Acid Metabolism	Tyrosine Concentration	NA	NA

doi	Author (year)	General Endpoint Category	More specific endpoint category	Most specific endpoint category	Reliability (NA = unrated)	Relevance to human health (NA = not relevant or unrated)
		Metabolism	Amino Acid Metabolism	Valine Concentration	NA	NA
		Metabolism	Lipid Metabolism	3 Hydroxyoctadecenoylcarnitine Concentration	NA	NA
		Metabolism	Lipid Metabolism	acl mRNA expression	NA	NA
		Metabolism	Lipid Metabolism	acox mRNA expression	NA	NA
		Metabolism	Amino Acid Metabolism	Arginine Concentration	NA	NA
		Metabolism	Lipid Metabolism	cpt1a mRNA expression	NA	NA
		Metabolism	Lipid Metabolism	fabp1 mRNA expression	NA	NA
		Metabolism	Lipid Metabolism	fas mRNA expression	NA	NA
		Metabolism	Lipid Metabolism	Malonylcarnitine Concentration	NA	NA
		Metabolism	Lipid Metabolism	mcad mRNA expression	NA	NA
		Metabolism	Lipid Metabolism	Oleylcarnitine Concentration	NA	NA
		Metabolism	Lipid Metabolism	ppar a mRNA expression	NA	NA
		Metabolism	Lipid Metabolism	ppar y mRNA expression	NA	NA
		Metabolism	Amino Acid Metabolism	Proline Concentration	NA	NA
		Metabolism	Lipid Metabolism	Propionylcarnitine Concentration	NA	NA
		Metabolism	Lipid Metabolism	srebp1c mRNA expression	NA	NA
		Metabolism	Lipid Metabolism	Stearoylcarnitine Concentration	NA	NA
10.1021/acs.est.9b03191	Luo (2019b)	Metabolism	Lipid Metabolism	acc mRNA expression	NA	NA
		Metabolism	Lipid Metabolism	Acetylcarnitine Concentration	NA	NA
		Metabolism	Lipid Metabolism	acl mRNA expression	NA	NA
		Alimentary, Excretory	Intestinal Tight Junctions	zo1 mRNA expression	NA	NA
		Metabolism	Lipid Metabolism	acox mRNA expression	NA	NA
		Metabolism	Amino Acid Metabolism	Alanine Concentration	NA	NA

doi	Author (year)	General Endpoint Category	More specific endpoint category	Most specific endpoint category	Reliability (NA = unrated)	Relevance to human health (NA = not relevant or unrated)
		Metabolism	Amino Acid Metabolism	Arginine Concentration	NA	NA
		Alimentary, Excretory	Intestinal Mucus Secretion	Gut Muscus Secretion	NA	NA
		Alimentary, Excretory	Intestinal Ion Transport	cftr protein expression	NA	NA
		Alimentary, Excretory	Intestinal Ion Transport	slc26a6 protein expression	NA	NA
		Alimentary, Excretory	Intestinal Mucus Secretion	muc1 mRNA expression	NA	NA
		Metabolism	Lipid Metabolism	Cholesterol Concentration	NA	NA
		Metabolism	Carb Metabolism	chrebp mRNA expression	NA	NA
		Metabolism	Carb Metabolism	coar mRNA expression	NA	NA
		Metabolism	Carb Metabolism	coas mRNA expression	NA	NA
		Alimentary, Excretory	Intestinal Mucus Secretion	muc2 mRNA expression	NA	NA
		Alimentary, Excretory	Intestinal Mucus Secretion	muc mRNA expression	NA	NA
		Alimentary, Excretory	Intestinal Mucus Secretion	klf4 mRNA expression	NA	NA
		Metabolism	Lipid Metabolism	cpt1a mRNA expression	NA	NA
		Alimentary, Excretory	Intestinal Mucus Secretion	renlb mRNA expression	NA	NA
		Alimentary, Excretory	Intestinal Mucus Secretion	meprinb mRNA expression	NA	NA
		Alimentary, Excretory	Intestinal Ion Transport	nkcc1 mRNA expression	NA	NA
		Alimentary, Excretory	Intestinal Ion Transport	cftr mRNA expression	NA	NA

doi	Author (year)	General Endpoint Category	More specific endpoint category	Most specific endpoint category	Reliability (NA = unrated)	Relevance to human health (NA = not relevant or unrated)
		Alimentary, Excretory	Intestinal Ion Transport	slc26a3 mRNA expression	NA	NA
		Metabolism	Carb Metabolism	cs mRNA expression	NA	NA
		Alimentary, Excretory	Intestinal Ion Transport	slc26a6 mRNA expression	NA	NA
		Alimentary, Excretory	Intestinal Ion Transport	nhe3 mRNA expression	NA	NA
		Alimentary, Excretory	Intestinal Ion Transport	ano1 mRNA expression	NA	NA
		Metabolism	Lipid Metabolism	dgat1 mRNA expression	NA	NA
		Metabolism	Lipid Metabolism	dgat2 mRNA expression	NA	NA
		Metabolism	Lipid Metabolism	fabp1 mRNA expression	NA	NA
		Metabolism	Lipid Metabolism	fas mRNA expression	NA	NA
		Metabolism	Lipid Metabolism	fat mRNA expression	NA	NA
		Metabolism	Lipid Metabolism	fatp2 mRNA expression	NA	NA
		Metabolism	Lipid Metabolism	Free Carnitine Concentration	NA	NA
		Metabolism	Carb Metabolism	gk mRNA expression	NA	NA
		Metabolism	Carb Metabolism	Glucose Concentration	NA	NA
		Metabolism	Carb Metabolism	glut2 mRNA expression	NA	NA
		Metabolism	Lipid Metabolism	gpat mRNA expression	NA	NA
		Metabolism	Lipid Metabolism	HDL Concentration	NA	NA
		Metabolism	Amino Acid Metabolism	Leucine Concentration	NA	NA
		Metabolism	Lipid Metabolism	Low Density Lipoprotein Concentration	NA	NA
		Metabolism	Lipid Metabolism	mcad mRNA expression	NA	NA
		Metabolism	Amino Acid Metabolism	Methionine Concentration	NA	NA
		Metabolism	Lipid Metabolism	mtp mRNA expression	NA	NA

doi	Author (year)	General Endpoint Category	More specific endpoint category	Most specific endpoint category	Reliability (NA = unrated)	Relevance to human health (NA = not relevant or unrated)
		Metabolism	Lipid Metabolism	Nonestrified Fatty Acid Concentration	NA	NA
		Fitness	Body Condition	Liver Index	NA	NA
		Metabolism	Amino Acid Metabolism	Ornithine Concentration	NA	NA
		Metabolism	Amino Acid Metabolism	Phenylalanine Concentration	NA	NA
		Metabolism	Carb Metabolism	pk mRNA expression	NA	NA
		Metabolism	Lipid Metabolism	ppar a mRNA expression	NA	NA
		Metabolism	Lipid Metabolism	ppar r mRNA expression	NA	NA
		Metabolism	Amino Acid Metabolism	Proline Concentration	NA	NA
		Metabolism	Lipid Metabolism	Propionylcarnitine Concentration	NA	NA
		Metabolism	Carb Metabolism	Pyruvate Concentration	NA	NA
		Metabolism	Lipid Metabolism	scd1 mRNA expression	NA	NA
		Microbiome	Diversity	Shannon Index	NA	NA
		Microbiome	Actinobacteria	Actinobacteria Genomic DNA	NA	NA
		Alimentary, Excretory	Intestinal Tight Junctions	claudin1 mRNA expression	NA	NA
		Alimentary, Excretory	Liver Histological Abnormalities	Ballooning Degeneration	NA	NA
		Metabolism	Lipid Metabolism	srebp1c mRNA expression	NA	NA
		Metabolism	Lipid Metabolism	Triglyceride Concentration	NA	NA
10.2147/IJN.S161369	Meszaros (2018)	Circulatory	Circulatory	Pulmonary Arterial Pressure	NA	NA
10.1166/jbn.2006.004	Molugu (2006)	Respiratory	Lung Histological Abnormalities	Lung Tissue Inflammation	NA	NA
10.1016/j.toxlet.2020.01.008	Park (2020)	Fitness	Body Condition	Body Weight	NA	relevant
		Immune	Immune Cells	White Blood Cell Count	NA	NA

doi	Author (year)	General Endpoint Category	More specific endpoint category	Most specific endpoint category	Reliability (NA = unrated)	Relevance to human health (NA = not relevant or unrated)
		Circulatory	Blood	Red Blood Cell Count	NA	NA
		Circulatory	Blood	Mean Corpuscular Volume	NA	NA
		Circulatory	Blood	Mean Corpuscular Hemoglobin	NA	NA
		Circulatory	Blood	Mean Corpuscular Hemoglobin Concentration	NA	NA
		Circulatory	Blood	Cellular Hemoglobin Concentration	NA	NA
		Circulatory	Blood	Red Blood Cell Distribution Width	NA	NA
		Circulatory	Blood	Mean Platelet Volume	NA	NA
		Immune	Immune Cells	Neutrophil Count	NA	NA
		Immune	Immune Cells	Lymphocyte Count	NA	NA
		Immune	Immune Cells	Monocyte Count	NA	NA
		Immune	Immune Cells	Eosinophil Count	NA	NA
		Immune	Immune Cells	Basophil Count	NA	NA
		Circulatory	Blood	Reticulocyte	NA	NA
		Circulatory	Blood	Hemoglobulin Concentration	NA	NA
		Circulatory	Blood	Hematocrit	NA	NA
		Circulatory	Blood	Hemoglobulin Distribution Width	NA	NA
		Circulatory	Blood	Platelet Count	NA	NA
		Respiratory	Lung Histological Abnormalities	Lung Histology	NA	relevant
		Alimentary, Excretory	Kidney Histological abnormalities	Kidney Histology	NA	relevant
		Alimentary, Excretory	Spleen Histological Abnormalities	Spleen Histology	NA	relevant
		Fitness	Reproduction	Testis Histology	NA	relevant

doi	Author (year)	General Endpoint Category	More specific endpoint category	Most specific endpoint category	Reliability (NA = unrated)	Relevance to human health (NA = not relevant or unrated)
		Alimentary, Excretory	Digestive Tract Histological Abnormalities	Stomach Histology	NA	relevant
		Fitness	Reproduction	Seminal Vesicle Histology	NA	relevant
		Fitness	Reproduction	Ovary Histology	NA	relevant
		Immune	Immune Cells	Natural Killer Cell Count	NA	NA
		Immune	Immune Cells	CD4+/CD8+ Ratio	NA	NA
		Immune	Immune Cells	CD11b+/CD11c- Ratio	NA	NA
		Immune	Immune Cells	CD11b+/CD11c+ Ratio	NA	NA
		Immune	Immune Cells	CD11b-/CD11c+ Ratio	NA	NA
		Immune	Immune Other	IgA Concentration	NA	NA
		Immune	Immune Other	IgG Concentration	NA	NA
		Immune	Immune Other	IgM Concentration	NA	NA
		Fitness	Reproduction	Number of Live Births	NA	relevant
		Immune	Immune Cells	Invariant Natural Killer Cells	NA	NA
		Immune	Immune Cells	T Cell Count	NA	NA
		Immune	Immune Cells	B Cells	NA	NA
10.1002/tox.23095	Wei (2020)	Circulatory	Heart Tissue	Heart Histology	unreliable	relevant
		Metabolism	Oxidative Stress	Malondialdehyde Concentration	reliable	NA
		Metabolism	Oxidative Stress	SOD Activity	reliable	NA
		Metabolism	Oxidative Stress	Glutathione Peroxidase Activity	reliable	NA
		Metabolism	Oxidative Stress	Catalase Activity	reliable	NA
		Circulatory	Heart Tissue	Creatine Kinase Myocardial Band	reliable	NA
		Circulatory	Heart Tissue	Cardiac Troponin I	reliable	NA
		Immune	Inflammation	IL18 Concentration	reliable	NA
		Immune	Inflammation	IL1b Concentration	reliable	NA

doi	Author (year)	General Endpoint Category	More specific endpoint category	Most specific endpoint category	Reliability (NA = unrated)	Relevance to human health (NA = not relevant or unrated)
		Cell Growth and Proliferation	Apoptosis and Cell Cycle	Apoptosis	NA	NA
		Immune	Inflammation	p-nfkB/nfkB Ratio protein expression	reliable	NA
		Immune	Inflammation	nlrp3 protein expression	reliable	NA
		Immune	Inflammation	Cleaved Caspase 1 protein expression	reliable	NA
		Immune	Inflammation	ASC protein expression	reliable	NA
		Immune	Inflammation	Cleaved Gasdermin D protein expression	reliable	NA
		Immune	Inflammation	IL1b protein expression	reliable	NA
		Immune	Inflammation	IL18 protein expression	reliable	NA
10.1021/nn700256c	Xia (2008)	Immune	Immune Cells	Neutrophil Count	NA	NA
		Stress	General Stress	Protein Concentration	NA	NA
		Metabolism	Energy Metabolism	Lactate Dehydrogenase Concentration	NA	NA
		Immune	Immune Cells	Macrophage Count	NA	NA
10.1016/j.ecoenv.2019.110133	Xie (2020)	Fitness	Body Condition	Body Weight	reliable	relevant
		Fitness	Reproduction	Spermatogenic Cell Concentration	reliable	relevant
		Fitness	Reproduction	Sperm Count	reliable	relevant
		Fitness	Reproduction	Sperm Deformity	reliable	relevant
		Metabolism	Energy Metabolism	Succinate dehydrogenase Activity	reliable	NA
		Metabolism	Energy Metabolism	Lactate Dehydrogenase Activity	reliable	NA
		Endocrine Signaling	Reproductive Endocrine Signaling	Testosterone Concentration	reliable	relevant

doi	Author (year)	General Endpoint Category	More specific endpoint category	Most specific endpoint category	Reliability (NA = unrated)	Relevance to human health (NA = not relevant or unrated)
		Metabolism	Oxidative Stress	ROS Production	reliable	NA
		Metabolism	Oxidative Stress	Malondialdehyde Concentration	reliable	NA
		Metabolism	Oxidative Stress	Glutathione Concentration	reliable	NA
		Stress	General Stress	p38 Phosphorylation	unreliable	NA
		Cell Growth and Proliferation	Apoptosis and Cell Cycle	Caspase 3 Concentration	reliable	NA
		Immune	Inflammation	TNFa Concentration	NA	NA
		Immune	Inflammation	IL1b Concentration	NA	NA
		Immune	Inflammation	Interleukin 6 Concentration	NA	NA
10.1016/j.jhazmat.2021.126092	Xu (2021)	Metabolism	Lipid Metabolism	Cholesterol Concentration	NA	NA
		Circulatory	Blood	Platelet Count	NA	NA
		Immune	Immune Cells	White Blood Cell Count	NA	NA
		Fitness	Body Condition	NA	NA	NA
		Fitness	Body Condition	Weight Gain	NA	NA
		Fitness	Body Condition	Testis Weight	NA	NA
10.1016/j.scitotenv.2020.143085	Zheng (2021)	Alimentary, Excretory	Liver Histological Abnormalities	Fat Vacuoles	NA	NA
		Alimentary, Excretory	Liver Histological Abnormalities	Inflammatory Cell Infiltration	NA	NA
		Immune	Inflammation	IL1b Concentration	NA	NA
		Immune	Inflammation	TNFa Concentration	NA	NA
		Immune	Inflammation	Interferon y Concentration	NA	NA
		Metabolism	Lipid Metabolism	Triglyceride Concentration	NA	NA
		Metabolism	Lipid Metabolism	ppar y Concentration	NA	NA
		Metabolism	Oxidative Stress	Malondialdehyde Concentration	NA	NA
		Alimentary, Excretory	Intestinal Permeability	Intestinal Permeability	NA	NA

Table S6. Summary of *in vivo* mammalian microplastics studies that passed all ‘red’ criteria and were evaluated by outside experts

Ref	Species (sex)	N/ dose	Polymer	Sizes	Shape	Exposure duration and route of exposure	Dose(s) (mg/ kg-day)	Reported statistically significant effect(s)	Summary of Expert Opinion(s)
Xie et al. 2020 10.1016/j.ecoenv.2019.110133	**Mice (male)**	10	PS	5 - 5.9 µm	Spheres	42 days oral gavage	0.43, 4.25, 43.77	↓ sperm count<br>↑ sperm deformity rate	**Dr. Gray** - Most robust and reliable of reproductive tox papers available;<br>Methods and analyses well described;<br>Small sample sizes;<br>Hemocytometer outdated method for counting sperm but can be done right;<br>Sperm numbers in epididymis similar to other studies for strain;<br>~4% abnormal sperm is low and 50% reduction in sperm count unlikely to impact fertility in mice<br><br>**Ruben Gandia -**<br>No dose-response;<br>Oxidative stress not firmly linked to decreased sperm count<br><br>**Dr. Flaws** - highly reliable sperm analysis;<br>Unusual dose-response is not uncommon;<br>Endpoints measured and associated pathways

Ref	Species (sex)	N/ dose	Polymer	Sizes	Shape	Exposure duration and route of exposure	Dose(s) (mg/ kg-day)	Reported statistically significant effect(s)	Summary of Expert Opinion(s)
									are highly conserved between rodents and humans<br><br>**Dr. Katja Teerds -**<br>proper controls are present, so design of experimental groups is OK<br>Histopathological examination of testes has too small group size (n=3) and is not done quantitatively or objectively;<br>Sperm quality analysis unreliable due to improper method of sperm collection);<br>Testosterone measurements in figure 3.c(E) are reliable;<br>Oxidative stress biomarkers are reliable (ROS, MDA,GHS)- figure 4;<br>Intact Caspase 3 is an unclear indicator of apoptosis, whereas activated CAspase 3 is better biomarker (figure 6A).
Park et al. 2020	**Mice (male/ female)**	10/15	PE	40-48 µm	Fragments	90 days oral gavage	3.75, 15, 60	↓ CD8/CD4 T Cells	**Dr. Gray** - statistics not significant (re-calculated); no significant effects observed

Ref	Species (sex)	N/ dose	Polymer	Sizes	Shape	Exposure duration and route of exposure	Dose(s) (mg/ kg-day)	Reported statistically significant effect(s)	Summary of Expert Opinion(s)
10.1016/j.toxlet.2020.01.008									
Deng et al. 2017 10.1038/srep46687	**Rats (male)**	5	PS	5, 20 µm	Spheres	28 days oral gavage	0.01,0.1, 0.5 mg/day	↑SOD, AChE, GSH-Px, LDH<br>↓ATP, T-CHO, TG, CAT<br>↑ inflammation, lipid droplets in liver	**Dr. Powell** - best (least poor) of three gut papers.Gavage is stressful and un-realistic exposure. hepatic inflammation, lipid droplet accumulation somewhat reliable. Metabolic data suggest dose-response and appear plausible.
An et al (2021) 10.1016/j.tox.2020.152665	**Wistar rats (female)**	8	PS	0.5 µm	Spheres (implied)	90 days Drinking water	0.083, 0.83, 8.3	↓ growing follicle count<br>↓ anti-Müllerian hormone<br>Oxidative stress, apoptosis of granulosa cells, ovary fibrosis<br>↑ Wnt, β-catenin, p-β-catenin,	**Dr. Susanne Brander -** in-line with AOP<br><br>**Dr. Flaws -** most reliable dose-response information<br><br>**Dr. Pauliina Damdimopoulou -**<br>Likely duplicative reporting with Hou et al 2021b Follicle counting procedure inadequately described and not systematic

Ref	Species (sex)	N/ dose	Polymer	Sizes	Shape	Exposure duration and route of exposure	Dose(s) (mg/ kg-day)	Reported statistically significant effect(s)	Summary of Expert Opinion(s)
								transforming growth factor-β (TGF-β), fibronectin, α-smooth muscle actin (α-SMA)	Oxidative stress markers measured using kits with inadequate descriptions<br>Western blot analyses not reported in standardized way,<br>Immunostainings do not have negative controls and do not report sample numbers or replicates<br>No negative controls included in flow cytometry<br>ELISA kit performance not explained<br>Unclear how many rats used for granulosa cell isolation<br>Microplastics well-characterized<br>Solid proof for microplastics distribution to ovaries<br>Long exposure period sufficient for follicle growth period<br>Relatively high number of animals per group<br>Most relevant endpoint to humans is AMH<br>OVERALL: low quality<br><br>**Dr. Katja Teerds -**<br>proper control present, design of experimental groups is OK;

Ref	Species (sex)	N/ dose	Polymer	Sizes	Shape	Exposure duration and route of exposure	Dose(s) (mg/ kg-day)	Reported statistically significant effect(s)	Summary of Expert Opinion(s)
									Follicle count is unreliable (unclear how follicle numbers were counted and not serial sectioned)- Figure 2A,B;<br>AMH count is reliable (Fig 2.C);<br>Collagen fiber expression unreliable (unclear descriptions of methods);<br>Fibronectin expression unreliable (lack of sample size reporting and appropriate controls for specificity of primary antibodies or endogenous peroxidase activity)- Fig 5;<br>Bax staining unreliable - fig 6A, B;<br>Bcl-2 staining somewhat reliable- fig 6C;<br>Wnt/β-catenin<br>signaling pathway-related protein expression Western blot data reliable (Fig 7)
Amereh et al (2020) doi.org/10.1	**Wistar rats (male)**	6	PS	0.025, 0.50 µm	Spheres	35 days Oral gavage	0, 1, 3, 6, 10	↓ testosterone<br>↓ luteinizing hormone<br>↓ follicle-stimulating hormone<br>↑ Lesions<br>↑ DNA damage	**Dr. Gray** - too much uncertainty including lack of variance estimate, small sample sizes, no standard histopathological evaluation, statistical significance for small effects;<br>Sperm count unreliable;<br><br>**Dr. Flaws** - reliable dose-response information

Ref	Species (sex)	N/ dose	Polymer	Sizes	Shape	Exposure duration and route of exposure	Dose(s) (mg/ kg-day)	Reported statistically significant effect(s)	Summary of Expert Opinion(s)
016/j.envpol.2020.114158								↑ sperm morphological alterations<br>↓ sperm viability<br>↓ sperm count<br>↓ *PLZF*<br>↓ *DAZL*<br>↓ *FSH*<br>↓ *LH*	**Dr. Katja Teerds -**<br>proper control present, design experimental groups is OK;<br>Testosterone, luteinizing hormone, follicle-stimulating hormone reliable (however FSH values are not perfectly dose-dependent)- figure 1;<br>Sperm count data reliable - table 1;<br>Sperm quality reliable -Figure 2;<br>Testicular histology mostly reliable (unclear how many tubules analysed and in how many animals) - Table 2 and Figure 3;<br>Tissue bioaccumulation reliable - fig. 4;<br>PLZF, DAZL, ABP reliable - fig 5A-C;<br>GnRH data somewhat reliable (figure seems to be cutoff and poorly labeled) - FIgure 5-D;<br>FSH and LH gene expression unreliable (did not report which tissue analyzed and counter plasma data) - Figure 5E-F
Li et al	**Rats**	8	PS	0.5 µm	Spheres	90 days		↑ Troponin I, creatine kinase-MB serum levels	**Dr. Brander -** in-line with mechanical damage AOP

Ref	Species (sex)	N/ dose	Polymer	Sizes	Shape	Exposure duration and route of exposure	Dose(s) (mg/ kg-day)	Reported statistically significant effect(s)	Summary of Expert Opinion(s)
(2020) 10.1016/j.envpol.2020.115025						Drinking water		Structure damage & apoptosis of myocardium, Collagen proliferation of heart	**Dr. Sue Marty -** uncertainties with test material
Li et al (2020) 10.1016/j.c	**C57BL/6 Mice (Male)**	20	PE	10-150 µm	Spheres	35 days food	2,20,200 ug/g feed	↑ gut microbial species, bacterial abundance, flora diversity<br>↑ Staphylococcus abundance<br>↓ Parabacteroides abundance<br>↑ interleukin-1α<br>↓ % of Th17 and Treg cells among CD4+ cells<br>↑ Intestinal inflammation	**Dr. Brander -** in-line with AOP, particles may be too large to translocate<br><br>**Dr. Powell -** methodology descriptions, experimental control poor. Increased gut diversity is positive outcome, poorly controlled study with ‘shotgun approach’

Ref	Species (sex)	N/ dose	Polymer	Sizes	Shape	Exposure duration and route of exposure	Dose(s) (mg/ kg-day)	Reported statistically significant effect(s)	Summary of Expert Opinion(s)
								↑TLR4, AP-1, and IRF5 expression	
Amereh et al. (2019) 10.1039/c9tx00147f	**Rats (Male)**	6	PS	25 and 50 nm	Spheres	35 days gavage	1,3,6,10 mg/kg body weight/day	↓ Free T3 and T4 serum concentrations<br>↑ T3/T4 ratio free (10 mg/kg) and total (1 mg/kg)<br>↑ GOT and GPT enzyme concentrations<br>↑ Cholesterol and LDL<br>↓ HDL	
Wei et al. 2020 10.1016/j.scitotenv.20	**Wistar rats (six-week)**	8	PS	510.4 nm	spheres	90 day Drinking water	0.087, 0.865, 8.56 mg/kg-body weight-day	↑ creatine kinase-MB<br>↑ cardiac troponin<br>Impaired mitochondria<br>↑ malondialdehyde	**Dr. Susanne Brander -** in-line with AOP<br><br>**Dr. Farraj -** study design and endpoints have merit, but missing key info (ingestion rates, animal sex, treatment regimen) and histopath is shaky. Cardiac tissue proteins (e.g. superoxide dismutase, IL-6, IL-18, caspase) are good

Ref	Species (sex)	N/ dose	Polymer	Sizes	Shape	Exposure duration and route of exposure	Dose(s) (mg/ kg-day)	Reported statistically significant effect(s)	Summary of Expert Opinion(s)
20.139935							(assumed! Drinking water rate not reported)	↓ superoxide dismutase<br>↓ glutathione peroxidase<br>↓ catalase<br>↑ interleukin-1β, IL-18<br>↑ NOD-like receptor protein 3 inflammasomes in cardiac tissue<br>↑ oxidative stress	indicators of inflammation, oxidative stress and are sound. Blood troponin and IL-6, IL-18 good indicators and sound.
Li et al (2021) https://link.springer.com/article/10.10	**Wistar rats (male, six-weeks)**	8	PS	0.500 um	spheres	90 day drinking water	0.083, 0.833, 8.33 mg/kg-day (based on initial body weight) and	↓ Sperm count, motility (8.3 mg/kg-d)<br>↑ Sperm abnormality (8.3 mg/kg-d)<br>↑ Bax (0.83, 8.3 mg/kg-d)<br>↓ Bcl-2 (0.83, 8.3 mg/kg-d)	**Dr. Jodi Flaws -** Consistencies with other studies<br><br>**Dr. Katja Teerds -**<br>proper control is present, so experimental design is OK;<br>Sperm quality (smear) reliable - Fig. 1A;<br>Sperm abnormity reliable - Fig. 1B;<br>Sperm concentration not very reliable (collected by cutting whole epidydmis) - Fig 1.C;

Ref	Species (sex)	N/ dose	Polymer	Sizes	Shape	Exposure duration and route of exposure	Dose(s) (mg/ kg-day)	Reported statistically significant effect(s)	Summary of Expert Opinion(s)
07/s11356-021-13911-9							reported dose in mg/d	↑TUNEL testis (0.83, 8.3 mg/kg-d)<br>↑ p38 phosphorylation, Nrf2 (0.83, 8.3 mg/kg-d)<br>↑ blood-brain barrier protein expression (0.83, 8.3 mg/kg-d)	Sperm motility not very reliably (collected by cutting whole epidydmis) - Fig 1.C;<br>Testicular histopathology not reliable due to non-quantitative data- Fig. 2;<br>Oxidative stress data reliable (CAT, GHS-PX, MDA, SOD) - Fig.3;<br>Immunohistochemical analysis not reliable due to improper or lack of controls (Bcl2, BAX) - Fig. 4;<br>Apoptosis via TUNEL staining somewhat reliable, but not perfect indicator of apoptosis - Fig.5;<br>P38 MAPK signaling reliable (p38, p-p-38, Nrf2) - Fig.6;<br>Blood-brain barrier protein expressions in rat testis reliable (Occludin, Connexin-43, N-Cadherin, Claudin-11) - Fig.7;<br>Nrf2, HO-1 reliable - Fig. 8;<br>Bax, Bcl2 reliable - Fig. 9;<br>Apoptosis via TUNEL staining somewhat reliable - Fig. 10;<br>Apoptosis of GC-2 cells via flow cytometry reliable - Fig.11

Ref	Species (sex)	N/ dose	Polymer	Sizes	Shape	Exposure duration and route of exposure	Dose(s) (mg/ kg-day)	Reported statistically significant effect(s)	Summary of Expert Opinion(s)
Hou et al (2021 a) 10.1016/j.jhazmat.2020.124028	**ICR rats (male, four-five weeks old)**	10	PS	5 μm	spheres	35 day drinking water	0.017, 0.17, 1.73 mg/kg-day	↓ testis weight (0.17 mg/kg-d)<br>↓ sperm survival<br>↑ sperm malformation<br>↑ NF-kB, p65<br>↑ inflammatory factors (IL-1β, IL-6,TNF)<br>↓ NRF2<br>↓ Bcl2 (anti-apoptotic)<br>↑ Bax (apoptotic)<br>↑ apoptosis (TUNEL)	**Dr. Gray -** too much uncertainty overall; Unclear determination of live sperm count; Reduction in testis weight not dose-dependent<br><br>**Dr. Flaws -** consistent and reliable endpoints when other studies considered<br><br>**Dr. Katja Teerds -**<br>proper control is present, so experimental design is OK;<br>Body weight and weight change reliable -Fig. 1-A,B;<br>Testis and epididymis organ coefficients not reliable (unclear reporting)- Fig. 1-C,D;<br>Sperm living rate not very reliable (unclear reporting) - Fig. 2;<br>Sperm abnormality rate reliable- Fig. 3, 4;<br>Testicular morphology unreliable due to subjective, qualitative assessment- Fig. 5;<br>Nf-kBp65 related expression reliable (IkBa, p-IkBa, p-NF-kBp65, NF-kBp65)- Fig. 6;<br>IL-1β, IL-6, TNFα protein expression in testis reliable - Fig. 7

Ref	Species (sex)	N/ dose	Polymer	Sizes	Shape	Exposure duration and route of exposure	Dose(s) (mg/ kg-day)	Reported statistically significant effect(s)	Summary of Expert Opinion(s)
Hou et al (2021 b) https://doi.org/10.1016/j.ecoenv.2021.112012	**Wistar rats (female, six-weeks old)**	8	PS	0.5 µm	spheres	90 day Drinking water	0.015, 0.15, 1.5 mg/kg-day	↓ growing follicles, thickness (0.17 mg/kg-d)<br>↑ oxidative stress (MDA)<br>↓ CAT, SOD, GSH-px<br>↑ NLRP3, cleaved-caspase-I in granulosa cells of ovary (gene expression, protein levels)<br>↓ AMH<br>↑ IL-1β, IL-18<br>↑ apoptosis (TUNEL granulosa cells in ovary)<br>↑ NLRP3, IL-1β, Cleaved-GSDMD, ASC protein expression	**Dr. Jodi Flaws -**<br>Consistencies with other studies and uses consistent methods<br><br>**Dr. Pauliina Damdimopoulou -**<br>Likely duplicative reporting with An et al 2021<br>Follicle counting procedure inadequately described and not systematic<br>Oxidative stress markes measured using kits with inadequate descriptions<br>Western blot analyses not reported in standardized way,<br>Immunostainings do not have negative controls and do not report sample numbers or replicates<br>No negative controls included in flow cytometry<br>ELISA kit performance not explained<br>Unclear how many rats used for granulosa cell isolation<br>Microplastics well-characterized<br>Solid proof for microplastics distribution to ovaries<br>Long exposure period sufficient for follicle growth period<br>Relatively high number of animals per group

Ref	Species (sex)	N/ dose	Polymer	Sizes	Shape	Exposure duration and route of exposure	Dose(s) (mg/ kg-day)	Reported statistically significant effect(s)	Summary of Expert Opinion(s)
									Most relevant endpoint to humans is AMH<br>OVERALL: low quality<br><br>**Dr. Katja Teerds -**<br>proper control group present, design experimental groups is OK;<br>Follicle count unreliable - Fig 2.;<br>Oxidative stress biomarkers reliable (GSH-Px, MDA, CAT, SOD) - Fig. 3;<br>Cleaved-caspase1 and Nlrp3 not very reliable (poor controls, lack of N)- Fig. 4;<br>IL-1β, IL-18, AMH reliable - Fig. 5;<br>Apoptosis by TUNEL staining somewhat reliable, but not perfect indicator of double stranded DNA breaks- Fig. 6;<br>Granulosa cell apoptosis rate unreliable (improper reporting) - Fig. 6-D;<br>Granulosa cell apoptosis reliable by flow cytometry (fig. 6-C), cleaved-caspase3 (Fig 6-E), cleaved-caspase 3/GADPH (Fig 6-F);<br>Nlrp3 and Caspase-1 signaling reliable (NF-kB, p-NF-kB, NLRP3, GSDMD, cleaved-GSDMD, GADPH) - Fig. 7

Table S7. RIVM's PROAST benchmark dose results for endpoints deemed 'somewhat reliable' or 'reliable' and relevant to human health

**Study**	**Endpoint**	**Critical Effect Size** (unitless fraction)	**Single model BMD** (mg/kg-day)	**Single Model BMDL** (mg/kg-day)	**Single Model BMDU** (mg/kg-day)	**Bootstrap BMDL (90% CI)** (mg/kg-day)	**Bootstrap BMDU (90% CI)** (mg/kg-day)	**Model with lowest AICC**
Amereh et al (2020)	Luteinizing hormone concentration	0.08	0.11	0.01	0.54	0.02	1.22	expon. M3
Amereh et al (2020)	Follicle stimulating hormone concentration	0.07	9.14	5.79	9.51	5.79	9.51	inverse expon. M3
Amereh et al (2020)	Testosterone concentration	0.50	0.03	0.00	2.63	0.00	2.63	Full model
Amereh et al (2020)	Sperm count	0.04	0.49	0.14	11.12	0.14	1.12	expon. M3
Amereh et al (2020)	Sperm motility	0.10	1.16	0.55	2.21	0.42	2.48	inverse expon. M3
Amereh et al (2020)	Sperm DNA damage	0.29	0.80	0.28	1.08	0.19	1.19	expon m.5
Amereh et al (2020)	Sperm maturity	0.03	1.08	0.63	1.71	0.65	2.21	expon. M3
Amereh et al (2020)	Sperm viability	0.08	1.59	0.95	2.42	1.06	2.78	expon m.3
Amereh et al (2020)	Sperm Deformity	0.34	0.38	0.06	1.22	0.08	0.79	Ln m3
An et al	Anti-Mullerian hormone	0.09	0.00	0.00	0.01	0.00	0.03	inverse expon.

(2021)	concentration							M3
Deng et al (2017)	Liver Condition Index	0.06	4.52	0.58	14.90	0.62	14.20	inverse expon. M3
Deng et al (2017)	Liver Condition Index	0.05	12.27	2.10	12.50	2.10	12.50	inverse expon. M3
Hou et al (2021a)	Testis Weight	0.10	0.10	0.02	1.48	557000.00	0.48	expon. m3
Hou et al (2021a)	Sperm deformity	0.49	0.12	0.03	0.17	0.02	0.25	LN m5
Li et al (2021b)	Sperm deformity	0.11	0.21	0.09	0.40	0.09	0.50	LN m3
Xie et al (2020)	Body weight	NA	NA	NA	NA	NA	NA	NA
Xie et al (2020)	Sperm count	0.33	0.27	0.01	6.86	0.01	6.21	LM m3
Xie et al (2020)	Sperm deformity	0.44	0.04	0.00	1.13	0.00	2.51	LM m3
Xie et al (2020)	Testosterone concentration	0.42	1.42	0.02	1200.00	0.00	9.27	LM m3

Supplementary Figures

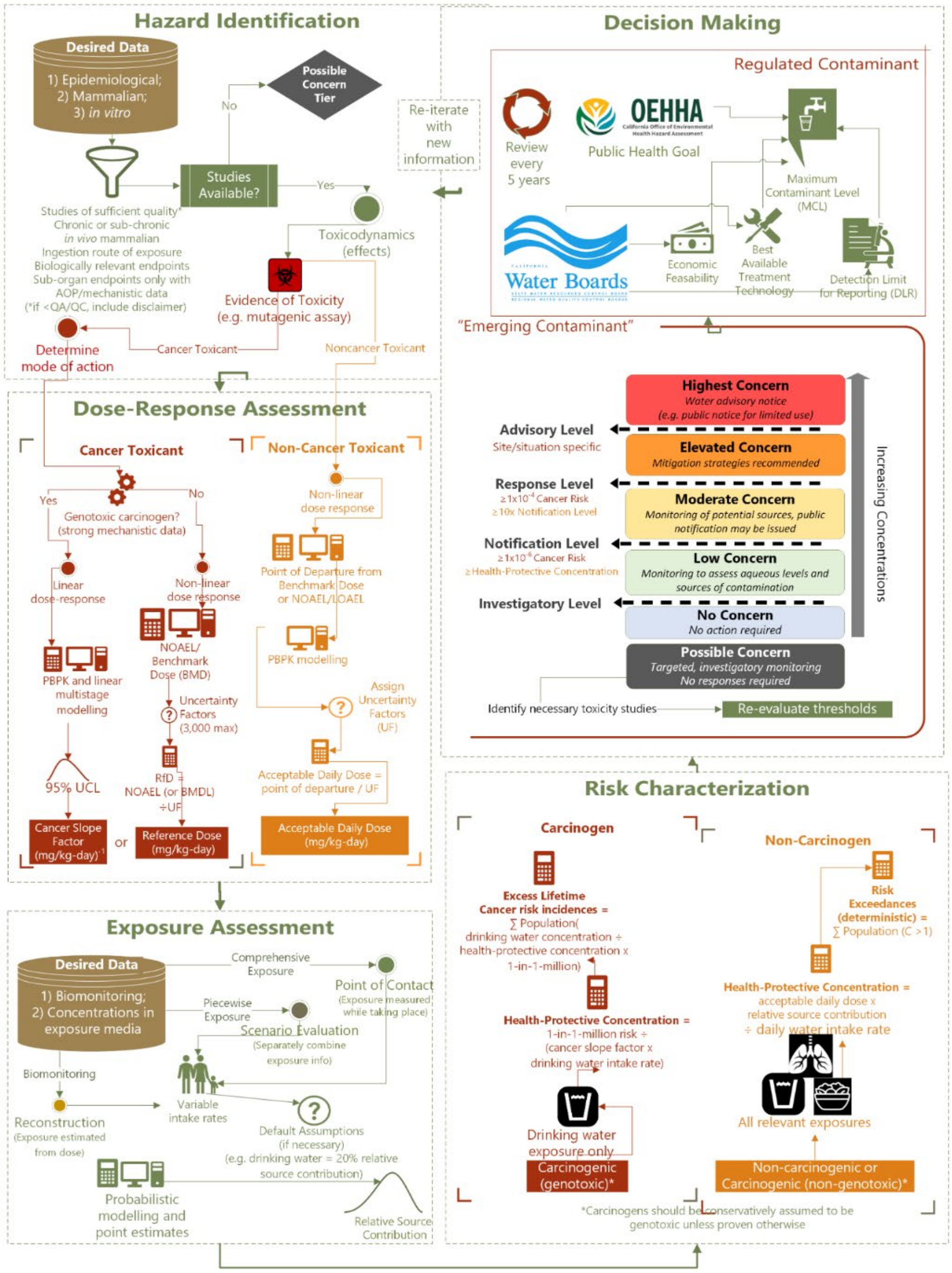

Figure S1. The overall framework utilized by the California State Water Resources Control Board for assessing and managing risks of drinking water contaminants.

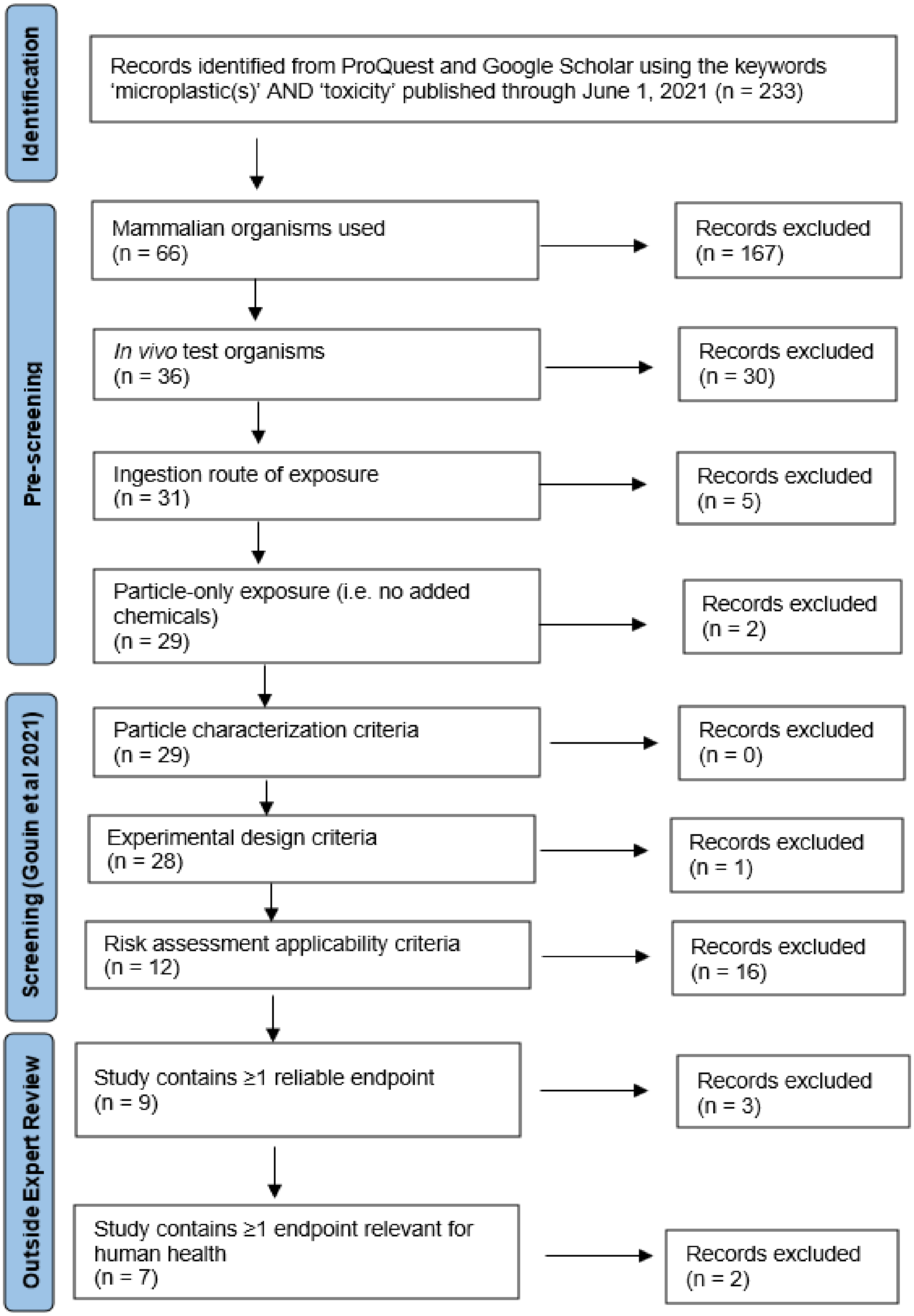

Figure S2. Diagram of identification, screening, and prioritization of microplastics toxicity studies.

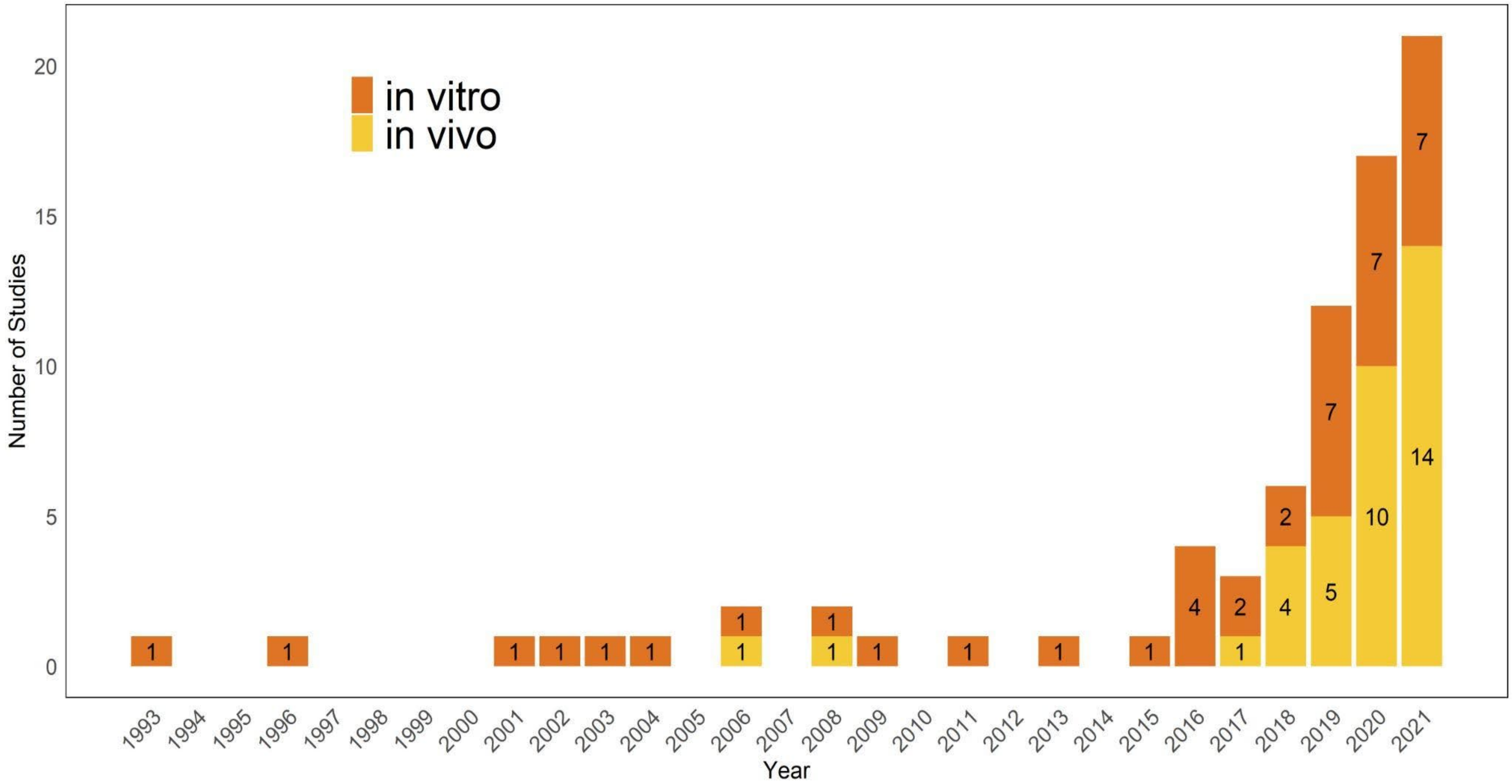

Figure S3. Overview of mammalian toxicity studies with ingestion route of exposure discovered in the systematic literature search arranged by year. Orange stacked bars represent in vitro studies, while yellow stacked bars represent in vivo studies. Note that studies that reported both in vivo and in vitro results are represented twice (i.e. as in vitro and in vivo studies).

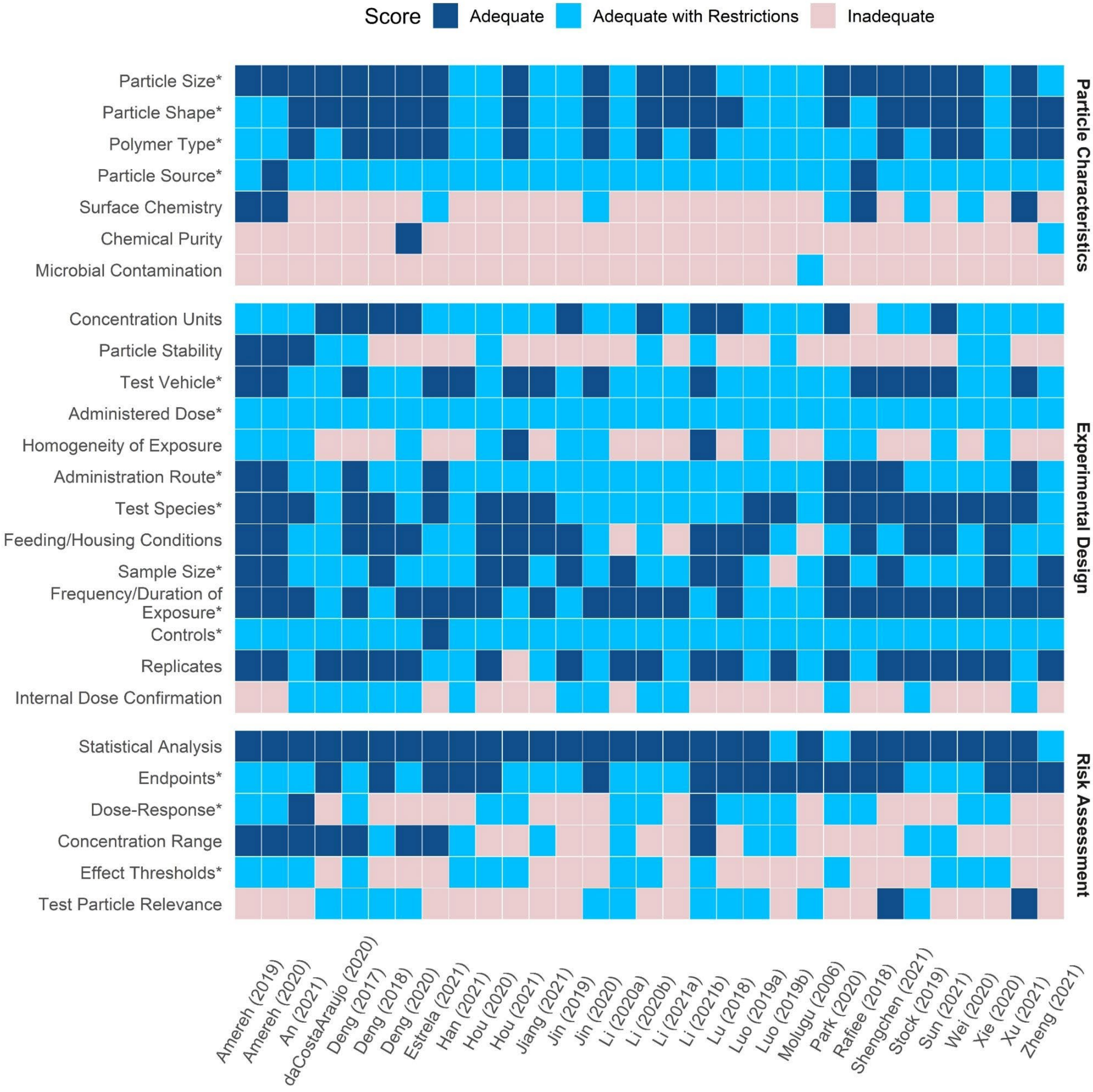

Figure S4. Heatmap of screening and prioritization criteria for in vivo mammalian toxicity studies with ingestion route of exposure. Pink represents a score of “0” (inadequate), light blue represents “1” (adequate with restrictions), and dark blue represents “2” (adequate). Criteria with asterisks (*) are minimal criteria that must be met to be considered ‘fit-for-purpose.’

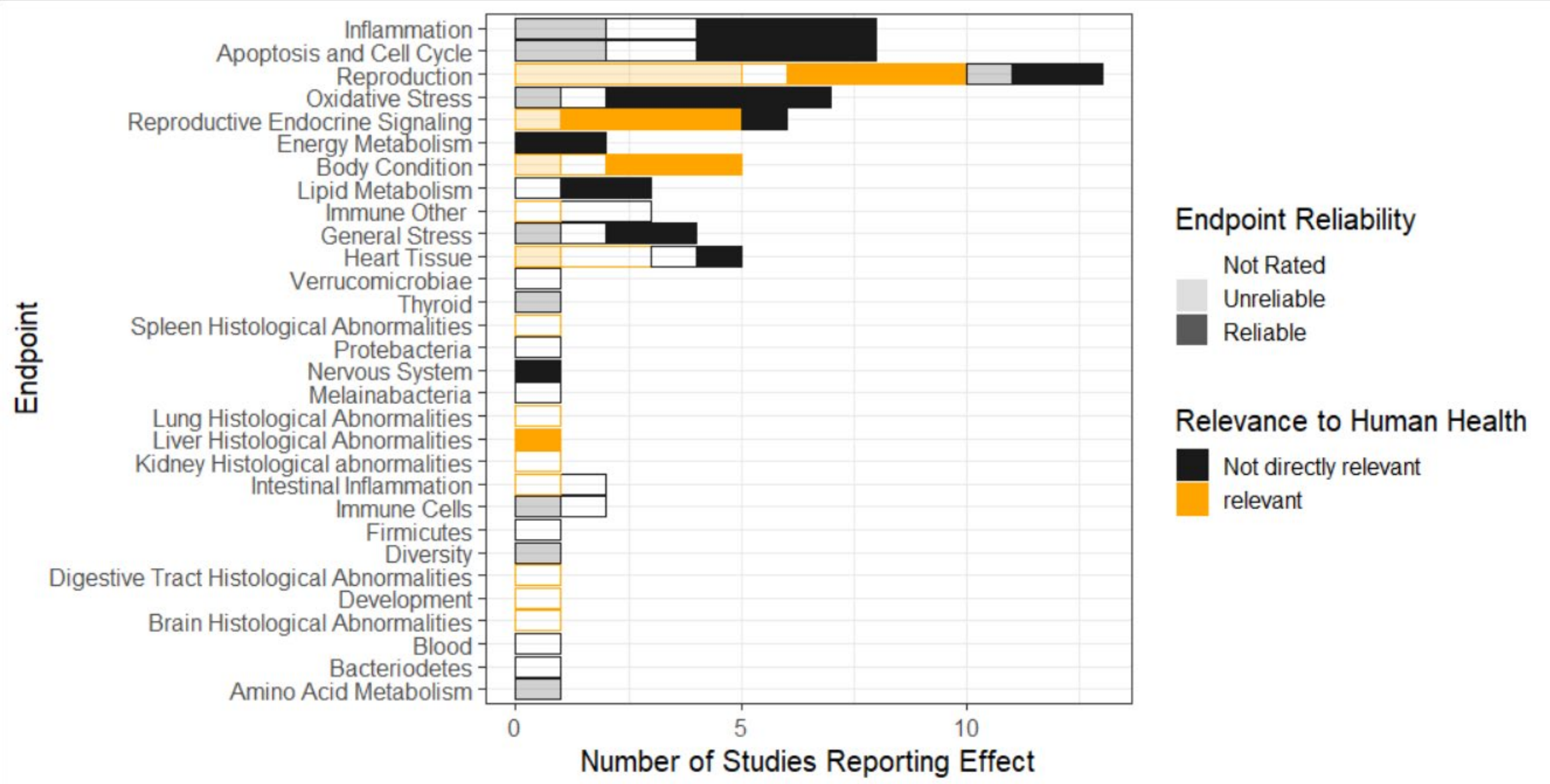

Figure S5. Summary of general endpoints reported in the database, colored by relevance to human health for risk assessment purposes. Transparency of bars indicates the number of studies/endpoints deemed reliable, somewhat reliable, unreliable, or not explicitly rated by outside experts. Only studies passing all ‘red criteria’ were assessed by outside experts.

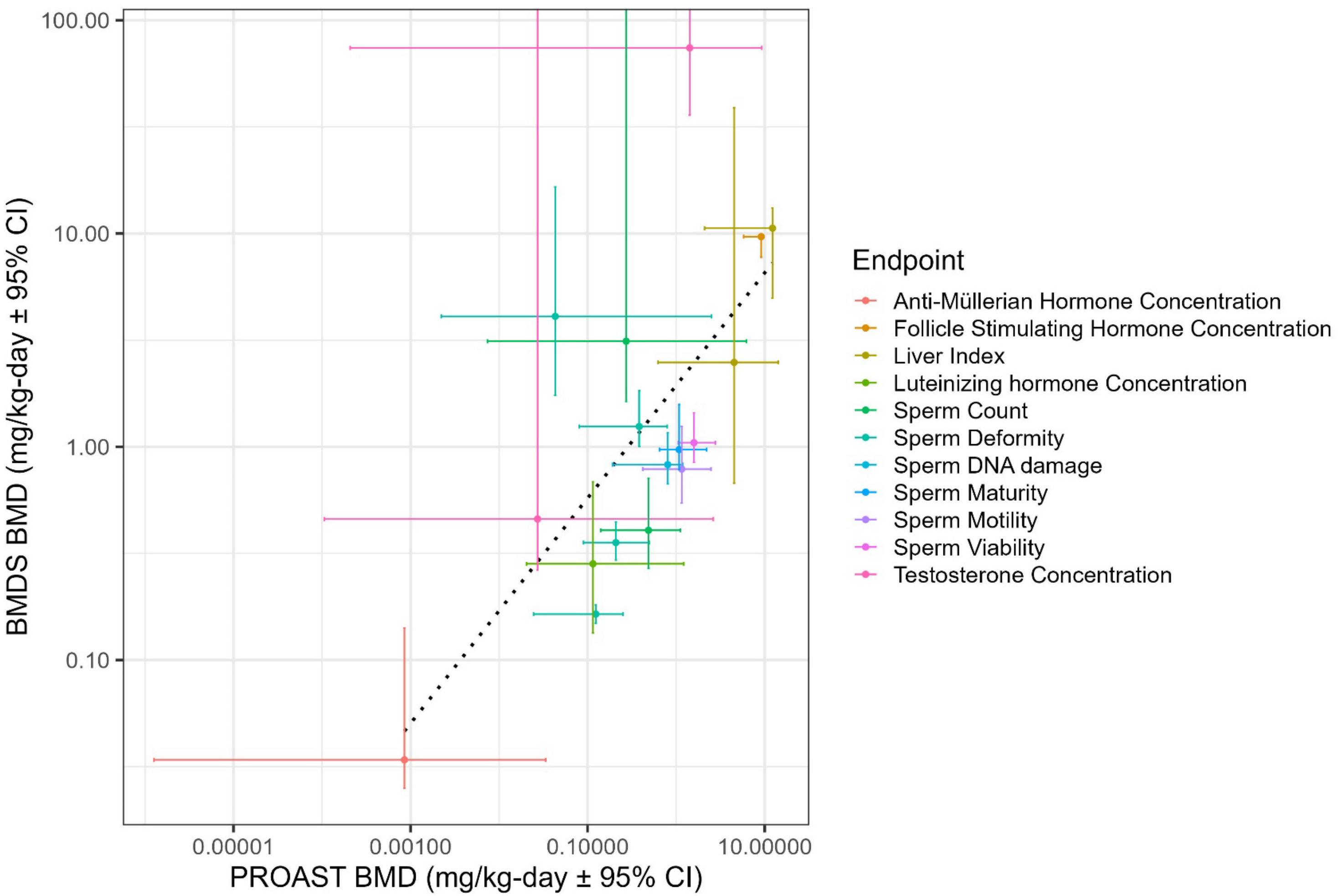

Figure S6. Benchmark dose model estimates (mg/kg-day) from RIVM's PROAST (x-axis) and EPA's BMDS (y-axis) for endpoints (color) deemed 'reliable' by experts. Points represent benchmark dose estimates with upper and lower 95% confidence intervals plotted as error bars.

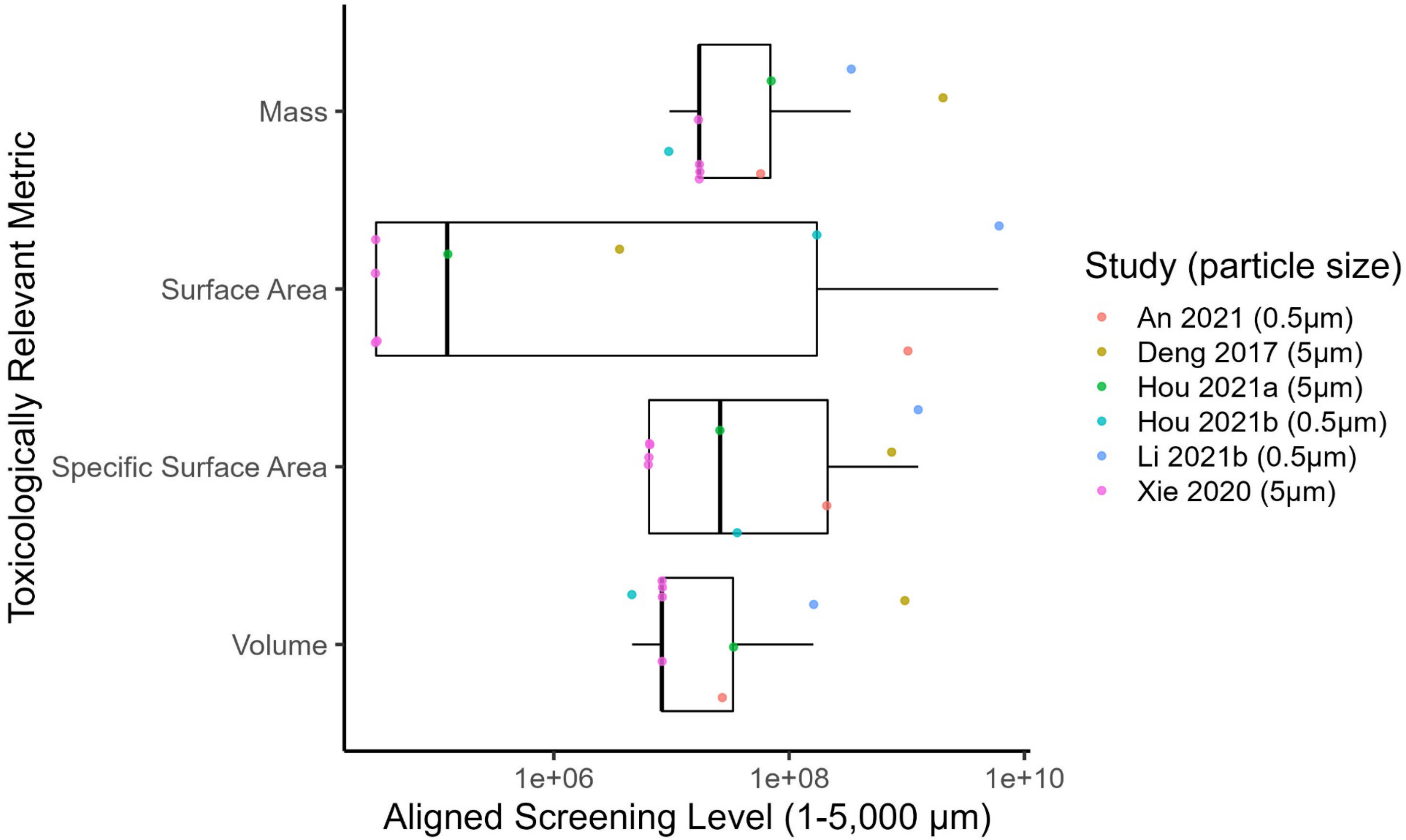

Figure S7. Sensitivity analysis of toxicologically relevant metrics (TRM) and point of departures. For each reliable endpoint point of departure, TRM-aligned screening levels were calculated based on particle mass, surface area, specific surface area, and volume and are compared in boxplots with all points shown. Colors correspond the study from which the aligned screening level was derived. The line

within each box marks the median, the left and right lines of the box indicate the first and third quartiles, and the whiskers indicate the minimum and maximum values.

Power Analysis for selected Publications

Xie et al. (2020)

In the study by Xie et al (Xie, et al., 2020), young adult mice were dosed daily by oral gavage with MPS for 42 days and then necropsied and the testis and sperm evaluated. Sperm from the epididymis were evaluated from 5 males per group using a hemocytometer microscope slide.The accuracy and precision of sperm count measurements in Xie et al (2020) were compared with data reported in published toxicology studies by Tyl et al. (Tyl et al., 2008a; Tyl et al., 2008b; Tyl et al., 2008c), (Cagen et al., 1999), and a review paper by Odet et al (Odet et al., 2015), and were deemed to be biologically plausible and fall within the range appropriate for this species. Xie et al (2020) reported that MPS exposed mice had significantly lower sperm counts than that in the control group and the sperm deformity rate gradually increased with dose. The sperm numbers in the epididymis are similar to those reported by Odet et al. (Odet, et al., 2015) (Table 1 and Figure 7 of that paper) for several mouse strains. It should be noted that the reduction in sperm count is not dose related over two orders of magnitude, and appears to plateau at around 50% of control and the increase in deformed sperm is dose related but even at the highest dose level is only about 4%.

Xie et al. (2020) reported a significant decrease in epididymal sperm count relative to saline vehicle control at all three exposure concentrations tested (0.43, 4.25, and 43.77 mg/kg-day). A linear relationship between sperm count and fertility was found over a wide range of concentrations in continuous breeding studies, indicating that there is no apparent threshold for adverse effects on reproduction (Chapin et al., 1997). While sperm count is not the most sensitive measure of testicular damage in rats or mice (Morrissey et al., 1988), epididymal sperm count has been demonstrated to be a more sensitive measure than spermatid head counts in testis (Gray et al. 1989; Ulbrich and Palmer, 1995).

No clear threshold exists for sperm counts in relation to fertility (Mangelsdorf and Buschmann, 2002). The World Health Organization provides lower range limits for semen characteristics which are associated with fertile men, summarized in Table 4-4 of Mangelsdorf and Buschmann (2002). Total sperm count of 40 x 10^6 are considered to be associated with fertility, among other parameters including semen volume, sperm concentration, motility, morphology and viability. According to Mangelsdorf and Buschmann (2002), sperm counts, sperm motility and sperm morphology are all different in fertile and infertile men in an analysis of several human reproductive studies. While fertility cannot be predicted on the basis of sperm counts alone, the incidence of infertility in humans increases continuously with reductions in sperm count (Meistrich and Brown, 1983). Sperm motility, followed by sperm count, is the best predictor of fertility according to a model that accurately predicts 70.4% of fertility in patients (Wang et al. 1988).

When comparing fertile men to infertile men in human studies, significant decreases in fertility are observed at ~50% reductions in sperm count (Macleod and Gold, 1951; Zukerman et al., 1977; David et al., 1979). As summarized in table 4-5 in (Mangelsdorf and Buschmann, 2002), percent fertility in men dramatically decreases from 60-200 x 10^6 sperm count/ml to 40-

60 x 10^6 sperm count/ml, with relative percent fertility decreases of 63 to 83% (Macleod and Gold, 1951; Zukerman et al., 1977; David et al., 1979).

To assess the adequacy of a sample size for a given endpoint to be suitable for detecting toxicity, the statistical power to detect effects should be considered for the endpoint and species of interest (Whitlock and Schluter 2015). Statistical power to detect effects is higher for endpoints with lower variability, and visa versa. Data (coefficient of variation or percentage difference which can be detected in a given sample size, or the power to detect a certain difference) for male reproductive toxicity endpoints for rats and mice from various publications are summarized in (Mangelsdorf and Buschmann 2002, 33–36).

When the standard deviation (σ) of an endpoint/species is known, the minimum detectable difference between means ($\mu_0 - \mu_1$; null hypothesis mean and alternative hypothesis mean, respectively) may be calculated for a given sample size (n) using the following equation:

Equation 1: $$\mu_0 - \mu_1 = \frac{\sqrt{21} * \sigma}{\sqrt{n}}$$

The constant in the numerator ($21^{1/2}$) is applicable for an α value of 0.05, where α is the probability of rejecting the null hypothesis when it is true (i.e. Type I error), and a β value of 0.10, where β is the probability of not rejecting the null hypothesis when it is false (i.e. Type II Error). Such values for Type I and Type II error are commonly used in biostatistics, as a β value of 0.10 corresponds to a statistical power of 0.90, and an α value of 0.05 corresponds to a 95% confidence interval (Belle and Millard 1998).

Mangelsdorf and Buschmann, (2002, 33–36) provide summary statistical power data for male reproductive toxicity endpoints in mice and rats in Tables 3-1, 3-2, 3-3, and 3-4, and utilize $\alpha = 0.05$ and power ($1 - \beta = 0.90$). Percent detectable differences for male reproductive toxicity endpoints for B6C3F1 mice from (Morrissey et al. 1988) are reported in Table 3-2. The authors report a minimum detectable difference of 50% for sperm count in epididymides in B6C3F1 mice for n = 10, β = 0.10 and α = 0.05.

Xie et al. (2020) used a sample size of n = 10 mice per exposure group and measured various toxicological endpoints for reproductive toxicity. A non-linear dose-dependent reduction in sperm count relative to control was reported for α = 0.05 in Figure 3.A of the respective manuscript. The authors did not report sperm count data in tabular form or in the main or supporting text, thus data were extracted using an online tool (Rohatgi 2014). Extracted and calculated data for this endpoint are presented in the table below.

Group	Calculated Polystyrene Exposure Concentration (mg/kg-day)	Sperm count (10^7/ml) Mean	Sperm count (10^7/ml) Standard deviation	P-value (Tukey HSD post-hoc, relative to control)	Percent Reduction relative to control
saline	0	0.994	0.29		0%
NAC	0	0.929	0.16		7%
SB203580	0	0.847	0.13		15%
0.01 mg/d PS	0.43	0.589	0.17	0.0043 **	41%
0.1 mg/d PS	4.25	0.603	0.19	0.006 **	39%
1 mg/d PS	43.77	0.451	0.31	0.001***	55%
1 mg/d PS + NAC	43.77	0.847	0.25		15%
1 mg/d PS + SB203580	43.77	0.69	0.28		31%

Xie et al. (2020) report a 41% reduction in sperm count for the lowest dose (0.43 mg/kg-day; total sperm count = 5.89 x 10^6/mL) relative to control (total sperm count = 9.94 x 10^6/mL) for BALB/C mice. Compared to the B6C3F1 mice in (Morrissey et al. 1988), the sample size used in Xie et al. (2020) (n = 10) would not be expected to be of large enough magnitude to detect a significant difference in sperm count at the alpha = 0.05 level and a power of 0.90. However, at the highest dose administered in Xie et al. (2020) of 43.77 mg/kg-day, a 55% reduction of sperm count relative to control was observed, which would be expected to be detectable according to the statistical power calculations in (Morrissey et al. 1988). Differences in mice strains, experimental design, and analytical methods may cause different variabilities in sperm count, and comparisons of measured parameters to other studies should therefore be used as a general guide for plausibility.

As noted by Mangelsdorf and Buschmann, (2002, 33–36), variations in sperm count in mice and rats are typically high, making their utility for assessing reproductive risks in humans of limited suitability. Mangelsdorf and Buschmann, (2002, 33–36) also note that sperm variability is lower in rats than in mice, and that sperm motility yields more reproducible results in all species.

Xie et al. (2020) reported a dose-dependent increase in sperm abnormality for exposure to polystyrene via drinking water in mice (n = 10). At the lowest concentration tested (0.43 mg/kg-day), sperm abnormality rate was 2.34%, or 65% higher than control. Given the sample size (n = 10), a 47% change in sperm abnormality would be expected to be detectable at the alpha = 0.05 level and a power of 0.90 for mice (Morrissey et al. 1988).

Group	n	Calculated Polystyrene Exposure Concentration (mg/kg-day)	Mean rate of teratosperm (%)	Standard Deviation of rate of teratosperm (%)	Percent change relative to control
saline	10	0	1.42	0.76	0%
NAC	10	0	1.83	0.69	29%
SB203580	10	0	2.07	0.50	46%
0.01 mg/d PS	10	0.43	2.34	0.60	65%
0.1 mg/d PS	10	4.25	2.59	1.09	83%
1 mg/d PS	10	43.77	3.76	0.86	164%
1 mg/d PS + NAC	10	43.77	2.00	0.60	41%
1 mg/d PS + SB203580	10	43.77	2.33	0.93	64%

Hou et al (2021)

Hou et al.(2021) reported significant decreases in testis weight at both the lowest and highest concentrations tested (0.017 and 1.73 mg/kg-day, respectively) relative to control (14.5 and 18.1% decrease, respectively). While testis weight changes do not indicate the nature of an effect, this endpoint is a sensitive indicator of male reproductive toxicity which is more sensitive than fertility, and such changes are generally considered sufficient evidence that an agent is a reproductive toxicant in animals (Moore et al., 1995; EPA, 1996).

Hou et al. (2021) used a sample size of n = 10 mice per exposure group and measured various toxicological endpoints for reproductive toxicity. A non-linear dose-dependent reduction in testis weight relative to control was reported for α = 0.05 in Figure 1.C of the respective manuscript. The authors did not report testis weight data in tabular form or in the main or supporting text, thus data were extracted from Figure 1.C using an online tool (Rohatgi 2014). Extracted and calculated data for this endpoint are presented in the table below.

**Exposure Group**	**Calculated Polystyrene Exposure Concentration (mg/kg-day)**	**N**	**Mean Testis Weight (g)**	**Standard Deviation**	**Percent Difference Relative to Control**
Control	0.000	10	0.0083	0.001	0%
L-Dose	0.017	10	0.0071	0.0006	14.46%
M-Dose	0.173	10	0.0074	0.0006	10.84%
H-Dose	1.729	10	0.0068	0.0008	18.07%

The authors reported a 14.46% reduction in total testis weight for the lowest dose tested (0.017 mg/kg-day) relative to control, which was deemed to be significant by the authors (α = 0.05). Morrissey et al. (1988) reported a detectable difference of 15% in testis weight for male B6C3F1 mice in a subchronic toxicity study for n = 10, β = 0.10 and α = 0.05. Accordingly, a reduction in testis weight of mice of such magnitude should be reliably detected at a 95% confidence level.

Hou et al. (2021) report a significant increase in sperm deformity rate relative to control in figure 4 of the respective manuscript. The authors did not report sperm malformation rate data in tabular form or in the main or supporting text, thus data were extracted from Figure 4 using an online tool (Rohatgi 2014). Extracted and calculated data for this endpoint are presented in the table below.

**Exposure Group**	**Calculated Polystyrene Exposure Concentration (mg/kg-day)**	**n**	**Sperm Malformation Rate (%) Mean**	**Sperm Malformation Rate (%) Standard Deviation**	**Absolute Percent Difference Relative to Control**
Control	0.000	10	9.16	5.48	0%
L-Dose	0.017	10	7.12	3.48	22.31%
M-Dose	0.173	10	14.18	1.43	54.81%
H-Dose	1.729	10	18.79	6.14	105.11%

Morrissey et al. (1988) reported a detectable difference of 47% in abnormal sperm percentage for male B6C3F1 mice in a subchronic toxicity study for $n = 10$, $\beta = 0.10$ and $\alpha = 0.05$. Accordingly, the observed sperm malformation rate relative to control of at the intermediate exposure concentration of 0.173 mg/kg-day should be detectable as significant at the $\beta = 0.10$ and $\alpha = 0.05$ levels, however it was not. The observed relative increase in sperm malformation for the highest exposure concentration of 1.729 mg/kg-day of 105% would be expected to be significant at the 95% confidence level, for which the authors reported that it indeed was.

Outside expert insights

Ruben Gandia, Health Canada

Xie et al. (2020)

There does seem to be a decrease in sperm count w MP alone...when they have the antagonist, it does seem to bring the count higher. the MOA seems far out that needs more thought...somehow the MP are causing oxidative stress which decr sperm count. There doesn't seem to be a dose response though.

Hou et al

Since its not a standard study its takes careful consideration on what the procedures actually were. And I dont see numbers… just graphs. They show selected slides but I would like to see actual numbers for that animal with some effects shown. It doesn't seem to have any effects on the final numbers of sperm count and or morphology though. So i dont see the concern here. Also i am wondering if there are indeed effects (and I don't think this study shows any real ones), it would be good to see the MPs in the testis since it would be physiologically improbable that sperm genesis is affected at all due to the testes=blood barrier.

Dr. Katja Teerds, Wageningen University
Katja Teerds, PhD
Human and Animal Sciences
Department of Animal Sciences
Wageningen University
De Elst 1
6709 WD
Wageningen
The Netherlands

Date received: June 4, 2021

Overall findings

The LH and testosterone data just did not follow the normal feedback rules which are that when testosterone is low LH should be high. They were both low. Reduced testosterone has an effect on spermatogenesis and in severe cases leads to a block in meiosis. FSH levels were in case of the lower doses decreased but in case of the highest dose not. Normally LH and FSH levels follow the same pattern. However one has to keep in mind that the major regulator of FSH production is not the negative feedback via testosterone but by inhibin produced by Sertoli cells. The data show that it is important not only to look directly at testis damage but also include the main endocrine regulators of spermatogenesis being LH, testosterone, FSH and preferably also inhibin.

Xie et al. (2020)

Experimental design:

Male mouse experiment - experimental group size n=10, proper controls are present, so design of experimental groups is OK (groups are: saline, NAC (antioxidant), SB203580 (p38MAPK inhibitor), 0.01 mg/d PS (5 – 5.9 um diameter), 0.1 mg/d PS, 1 mg/d PS, 1 mg/f PS + NAC, 1 mg/d PS + SB203580); duration exposure – 42 days)

Analysis reliability experiments performed:

- <u>Histopathological examination testis</u>
  - Figures 2 and S3A – group size n=3 (too small to draw reliable conclusions), no quantitative analysis of testis histology is performed, subjective conclusions drawn; immunohistochemical analysis – absence of a proper control regarding the specific binding of the primary antibody makes it difficult to draw conclusions from these data. Furthermore, not indicated whether all sections were stained in one run (required in case of quantification of immunohistochemical data). DAB staining quantified - not indicated whether there was corrected for background staining, also essential. Normally immunofluorescence is used for quantification

of immunohistochemical data as this method is more reliable than DAB staining. Together with the absence of a proper quantification and control these histology/immunohistochemical analysis data great caution needs to be considered by drawing any conclusions from these data.

- Sperm quality analysis
  - (figure 3A, B, n=5) as authors have not analysed sperm motility (which they could not do according to the method of sperm collection used, the data presented can be considered reliable.
  - Figure 3C-E data can be considered reliable.
  - Same holds true for the data presented in Figure 4.
  - Figure 6A Relevance of measuring testicular caspase 3 levels is unclear. Activated caspase 3 is an indicator of apoptosis, intact caspase 3 as measured by the authors does not provide any information – is present in most cell types in the testis.

An et al (2021)

Experimental design:

Female rat experiment – experimental group size n=8, proper control present, design of experimental groups is OK (groups are: 0, 0.015, 0.15 and 1.5 mg/d PS-MPs (0.5 um in diameter; duration exposure – 90 days)

Analysis reliability experiments performed:

- Histological analysis of ovaries (Figure 2, n=6)).
  - From the M&M it is unclear how follicle numbers were obtained. Authors indicate they have counted selected sections. Ovarian follicles are not evenly distributed throughout the ovary and therefore to obtain an impression on effects of a compound of ovarian follicle numbers, ovaries should be serial sectioned and every 5ht section should be analysed (for details see Slot et al 2006, Meng et al., 2016).
- Data presented in Figure 2A,B are not reliable, AMH levels in Figure 2C are fine.
- Data presented in Figure 3 seem fine.
- Figure 4 – It is not clear in how many different animals per group this analysis was performed, not is it described how many sections per ovary were analysed. Considering the highly variable structure of the ovary (within one ovary some areas will contain more connective tissue than others) I consider these data not reliable.
- The same holds true for Figure 5, although here immunohistochemistry was performed. In general, it becomes apparent from the methods section that it no controls for the specificity of the primary antibodies are described, as well a block of endogenous peroxidase activity is not included. This makes it impossible for authors to deduce background staining from their quantification of the immunostaining. As I indicated before, DAB staining is not an appropriated method to quantify immunostaining in

sections. Furthermore, authors have not indicated that all staining's have been performed in one run (essential if you intend to compare different animals) nor is indicated how many animals were included per group and how often the immunostaining was repeated. I consider the data presented in Figure 5 as unreliable.

- The same holds true for the data presented in Figure 6 A-C. Regarding the data in Figure 6C, this experiment seems fine, however from the M&M section it is not clear how frequently the experiment was repeated. What is shown is technical variation within one cell analysis experiment, not biological variation between repeated cell isolations (which is what one should do). Hence, one needs to take this into account when drawing any conclusions.
- Figure 7 Western blotting seems fine.

Amereh et al (2020)

Experimental design:

Rat experiments – experimental group size n=6, proper control present, design experimental groups is OK (0, 1, 3, 6, 10 mg/kg bw/d, particle size not indicated; duration exposure – 35 days)

Analysis reliability experiments performed:

- Data from Figure 1 seem reliable, though not straight forward. The fact that both testosterone and LH are significantly decreased suggests that there is an effect on the pituitary as well. FSH values are difficult to explain.
- Table 1 – sperm collected in the appropriate way; data are reliable.
- Same holds true for Figure 2.
- Figure 3 Magnification is somewhat low to be able to identify changes in spermatogenesis. Authors have performed a simple analysis on the seminiferous tubules, they measured tubule diameter and seminiferous epithelial height (Table 2). These are reliable analyses as such, however authors fail to describe in the M&M how they determined these parameters, how many tubules were analysed and in how many animals this analysis was performed. Same holds true for the measurement of the capsule thickness. These limitations need to be considered when interpreting these data.
- Data Figure 4 seem to be fine.
- Figure 5A-C data seem fine. It is unclear which gene is analysed in Figure 5D, neither did authors indicate in the figure legend in which tissue they analysed FSH and LH expression, I presume pituitary. If this is indeed the case, the qPCR data for LH and FSH expression are **not** in line with the hormone measurements (LH gene expression is increased while plasma LH levels are decreased).

Li et al (2021)

Experimental design:

Male rat experiment – experimental group size n=8, proper control is present, so experimental design is OK (groups are: 0, 0.015, 0.15 and 1.5 mg/d PS-MS, 0.5 um in diameter; duration exposure – 90 days)

Analysis reliability experiments performed:

- Figure 1A data is fine (I come back to Figure 1B below). Regarding Figure 1C, D - sperm was collected by cutting open the whole epididymis, which makes it difficult to analyse sperm motility in a reliable way as only sperm present in the cauda epididymis has acquired motility. Recent ejaculation by the mouse may affect the relative amount of motile sperm in the sample (as sperm from whole epididymis has been sampled). Therefore, effects on sperm motility are considered not very reliable and need to be considered with caution. Regarding sperm abnormality, this maybe overestimated when one compares this to other studies that have collected sperm from the cauda and not from the whole epididymis. However, sperm does not acquire abnormalities in the epididymis, this is initiated in the testis. As such the data from Figure 1B is OK.
- Figure 2 – this is a qualitative analysis. It is not clear from the M&M how many animals were included in this analysis, not how many tissue sections were studies. The absence of any form of quantitative analysis makes it very difficult to draw any conclusion regarding the histology. The abnormalities show can be found on rare occasions also in control animals. So data are inconclusive.
- Data Figure 3 seem fine.
- Figure 4 - immunohistochemical analysis for the presence of BcL2 and BAX – absence of a proper control regarding the specific binding of the primary antibody. Neither did authors indicate whether sections were stained in one run (essential if one intends to quantify immunostaining). DAB staining quantified, not indicated whether there was corrected for background staining. Furthermore, authors do not indicate the number of animals included in this analysis, nor how often the staining was repeated. Normally immunofluorescence is used for quantification of immunohistochemical data as this method is more reliable than DAB staining. Moreover, BcL2 and BAX are not the best markers for apoptosis, cCasp 3 is a better marker. Together these immunohistochemical data need to be considered with great caution.
- Figure 5 – Tunnel staining as marker for apoptosis has as disadvantage that Tunnel does not discriminate between single stranded DNA breaks (hallmark of apoptosis) and double stranded DNA breaks (hallmark of necrosis). Staining for the presence of cCasp3 is a better way to determine cellular apoptosis. Quantification of fluorescence is fine.
- Figure 6 seems fine. Same holds true for figure 7.

Hou et al (2021a)

Experimental design:

Female rat experiment – experimental group size n=8, proper control group present, design experimental groups is OK (groups are: 0, 0.015, 0.15, 1.5 mg/kg bw/d PS-MS 0.5 um in diameter; duration exposure – 90 days)

Analysis reliability experiments performed:

- Figure 2 - From the M&M it is unclear how follicle numbers were obtained. Authors indicate they have counted selected sections. Ovarian follicles are not evenly distributed throughout the ovary and therefore to obtain an impression on effects of a compound of ovarian follicle numbers, ovaries should be serial sectioned and every 5ht section should be analysed (for details see Slot et al 2006, Meng et al., 2016). Furthermore, it is unclear how many ovaries were analysed.
- Figure 3 seems fine.
- Figure 4 – Immunohistochemical staining for cCasp1 and Nlrp3 – absence of a proper control regarding the specific binding of the primary antibody. Neither did authors indicate whether sections were stained in one run (essential if one intends to quantify immunostaining). DAB staining quantified, not indicated whether there was corrected for background staining. Furthermore, authors do not indicate the number of animals included in this analysis, nor how often the staining was repeated. Normally immunofluorescence is used for quantification of immunohistochemical data as this method is more reliable than DAB staining. Due to these omission I qualify these data not very reliable.
- Figure 5 seems fine.
- Figure 6 – Tunnel staining as marker for apoptosis has as disadvantage that Tunnel does not discriminate between single stranded DNA breaks (hallmark of apoptosis) and double stranded DNA breaks (hallmark of necrosis). Staining for the presence of cCasp3 is a better way to determine cellular apoptosis. Quantification of fluorescence is fine. Figure 6D Unclear what authors have analysed here and how. This is not explained in the M&M nor in the figure legend. Figure 6 C, E and F are fine.
- Figure 7 is fine.

Hou et al (2021b)

Experimental design:

Male mouse experiment – experimental group size n=10, proper control is present, so experimental design is OK (groups are: 0, 100, 1000 and 10 ml/L PS-MPs, particle size 5 um in diameter; duration exposure 36 days)

Analysis reliability experiments performed:

- Figure 1A, B are fine. It is not clear how authors determined the organ coefficients in Figure 1C, D.
- Figure 2 – it is not clear what authors consider live sperm as they collected sperm from the complete epididymis and not from the cauda. This means that their sperm sample contains a mixture of motile and immature immotile sperm. The immotile sperm does not need to ne necessarily considered to be dead. I consider this data not very reliable. The number of animals is not indicated.
- Figures 3 and 4 are fine, though authors have not indicated in how many animals sperm morphology was determined.
- Figure 5 – Qualitative analysis of testicular morphology – conclusions are based on the author's impression not on a reliable quantification of the testicular tissue. Hence, without a proper quantitative analysis of the testis tissue I consider this data not reliable. Authors also have not indicated how many testicular areas were analysed nor the number of animals is indicated.
- Figures 6-9 seem fine.
- Figure 10 - Tunnel staining as marker for apoptosis has as disadvantage that Tunnel does not discriminate between single stranded DNA breaks (hallmark of apoptosis) and double stranded DNA breaks (hallmark of necrosis). Staining for the presence of cCasp3 is a better way to determine cellular apoptosis.
- Figure 11 seems fine.

Jin et al. (2021)

Experimental design:

Male mouse experiment – experimental group size n=12 for experiment 1, proper control group included, so experimental design for this experiment is OK (groups are: 0, 0.5, 4 and 10 um in diameter PS-MPs, one size per group; duration experiment 28 days); experimental group size experiment 2 n=3 (same groups given fluorescent PS-MPs, duration exposure 24 h) Comment – group size of 3 is small and difficult to analyze statistically

Analysis reliability experiments performed:

- Figure 3 seems fine.
- Figure 4A – Qualitative analysis of testicular morphology – conclusions are based on the author's impression not on a reliable quantification of the testicular tissue. Hence, without a proper quantitative analysis of the testis tissue I consider this data not reliable. Authors also have not indicated how many testicular areas were analysed nor the number of animals is indicated. Figure 4B seems fine but only testosterone levels do not say much. LH and FSH levels should also be analysed. In one of the other articles, it was shown that both testosterone and LH levels were reduced, indicative of an effect on the pituitary and not an effect on the testis (or both is also possible).

- Figure 5 seems more or less fine, though it is not clear how many epididymi were analysed as well as the number of sperm. In interpreting the sperm viability data one has to take into account that the authors have collected sperm from the whole epididymis, including the non-motile sperm. If the PS-MP treatment leads to a sperm maturation arrest in the epididymis, this can affect the viability as measured in this paper. A better way to determine sperm viability is by collecting sperm from the cauda epididymis and perform a swim up experiment as the authors did. Hence, I do not trust the viability data.
- Figure 6A The proper control to check the specificity of the primary antibody has not been performed. Staining is found in the interstitium where this has been described before. Authors have not performed quantification of the immunofluorescent data so one can accept these data as the are. Figure 6B seems fine.
- Figure 7 Authors claim that they show proof for a disruption of the blood-testis barrier due to PS-MP treatment. I have serious doubts about this conclusion as in that case with the high level of inflammatory markers one would expect an orchitis (infiltration of the seminiferous tubules by immune cells and massive disruption of spermatogenesis), which does not seem to be the case. I would therefore be very cautious regarding the conclusion by the authors that the blood-testis-barrier is disrupted. The TEM evidence is too limited for this. Furthermore, the Western blot for barrier markers (Figure 7C) was not quantified, hen provides only subjective information.

Dr. Leon Earl Gray Jr., U.S. EPA

Date received: 4/7/2021

Xie et al. (2020)

The publication by Xie et al., (Xie *et al.* 2020) seems to be the most robust and reliable study in this group of papers. The methods and analyses are reasonably well described among this group of publications for the assessment of the adult male reproductive toxicity of MPS in drinking water.

All these studies used fairly small samples sizes. Carl Sagan warned (Sagan 1995) that scientists should beware of statistics of small sample sizes which is a limitation of the literature on the reproductive toxicity of MPS at this time.

Another observation on the methods in the studies on the effects of MPS on epididymal sperm is that these studies all were done using a hemocytometer microscope slide, a method developed in the 19th century for counting blood cells. While many laboratories now use some automated method of counting sperm from the epididymis (some of these automated methods also calculate a variety of sperm motility parameters) rather than with a hemocytometer slide, all these methods can produce high quality data if done properly or inaccurate and highly variable data if not done properly. Detailed methods for cell counting using a hemocytometer are widely available in published protocols (Wang 2003), on the internet (for example, http://www.vivo.colostate.edu/hbooks/pathphys/reprod/semeneval/hemacytometer.html) and in government documents (WHO 2010).

In this regard, comparing the accuracy and precision of the data in each study being evaluated is important to determine if the results are biologically plausible; meaning that the control values fall with a range appropriate for this species and the data display reasonable precision. For example, studies with mice that can be used for comparison with the data in the study under review include published toxicology studies by Tyl et al. (Tyl *et al.* 2008a; Tyl *et al.* 2008b; Tyl *et al.* 2008c), (Cagen *et al.* 1999), and a review paper by Odet et al (Odet *et al.* 2015).

In the study by Xie et al (Xie *et al.* 2020), young adult mice were dosed daily by oral gavage with MPS for 42 days and then necropsied and the testis and sperm evaluated. Sperm from the epididymis were evaluated from 5 males per group; a relatively small sample size, but not different than the other studies that were evaluated.

The authors reported that MPS exposed mice had significantly lower sperm counts than the control group and the sperm deformity rate gradually increased with dose. The sperm numbers in the epididymis are like those reported by Odet et al. (Odet *et al.* 2015) (Table 1 and Figure 7 of that paper) for several mouse strains, although the sperm abnormality seems low. However, the reduction in sperm count is not dose related over two orders of magnitude and appears to plateau at around 50% of control and the increase in deformed sperm is dose related but even at the highest dose level is only about 4% (Figure below). These alterations alone

would not be expected to affect male fertility, which is consistent with the lack of effect of MPS on fertility at doses as high as 60 mg/kg/d in the study by Park et al. (2020). Reductions in sperm numbers less than 90% generally do not reduce fertility unless accompanied by other sperm defects or reproductive abnormality (Gray 2017; Meistrich 1989).

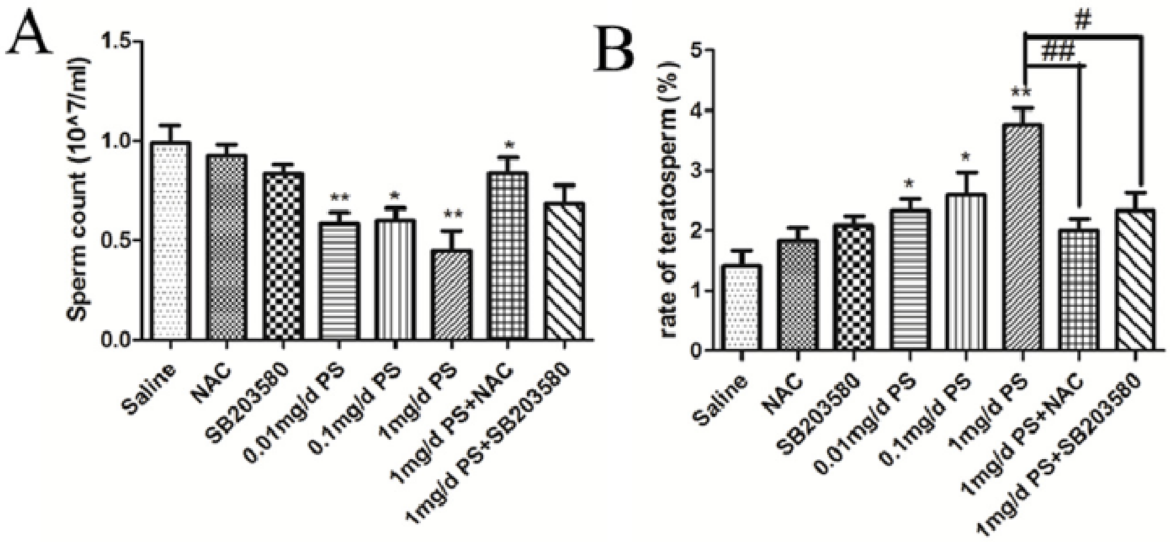

Amereh et al. (2020)

In the study by Amereh et al (Amereh *et al.* 2020) I believe there is too much uncertainty about the results to use for hazard assessment. The limitations include a lack of variance estimates for the data, small sample sizes, no standard histopathological evaluation of the testis or epididymis and statistical significance for surprisingly small effects.

They dosed six male mice per dose group for 5 weeks by oral gavage.

Sperm concentration, motility, and abnormalities were measured. Hormone levels also were measured using ELISAs rather than by RIAs or other more specific assays.

The methods state that “A piece of left caudal epididymis of each animal was minced and stored with 1 mL of human tubular fluid culture (HTF) containing 4 mg/mL BSA pre-warmed to 37°C in a CO2 incubator until analysis. The epididymal sperm was diluted to 1:20 in HTF.”

The sperm “count” data are reported as sperm concentration per mL but not as total sperm per epididymis. Since an undefined piece of the epididymis was taken for assessment it is not clear r what percent of the total epididymis was sampled it is not possible to determine how the reported value relates to total sperm numbers in the epididymis. It is also not possible to compare these results to those reported in the literature on mouse sperm counts.

More significantly, the data on sperm concentration in Table 1 and testis histology measures in Table 2 do not contain any estimates of variance, only mean values, as such it is not possible to critically review or reanalyze the reported statistically significant effects. In these tables some relatively small differences from control are reported as statistically significant.

Given the small sample sizes per group and the minimal magnitude of the reported effects of MPS, based upon similar data in the literature the reported differences do not seem credible.

Hou et al. (2021)

In the study by Hou et al. (Hou *et al.* 2021) I believe there is too much uncertainty about the validity of the analysis of these data for it to be used for hazard assessment of MPS in water.

They dosed 10 mice per dose group and analyzed 5 per group for testis histopathology (too few for a categorical endpoint).

Regarding relative testis weight from Figure 1, there was a variable non dose related reduction in relative testis weight but the absolute data on testis weight are not presented.

Further, sperm quality was presented as percent of living sperm. It is not clear how "living versus nonliving sperm" was determined. Figure 2 states that samples were counted 3 times but says the n=30, suggesting that the degrees of freedom and p values in the analysis are wrong. With 10 males per group and 3 counts per sample that would be a total sample size of 30 but the 3 recounts of a sample are not independent observations and should be averaged providing a single mean live sperm value per male and statistical n=10 for determining degrees of freedom. The significance of the effects therefore is uncertain.

No sperm count data are shown.

Park et al. (2020)

The study by Park et al. (Park *et al.* 2020) is a one generation study in which 15 male and female mice per dose group were exposed to MPS for 90 days by oral gavage. Five pairs of mice per group were mated at 80 to 89 days of age and at 90 days of age 10 non-mated females and 15 males per group were necropsied and evaluated. Dosing continued for five mated female mice/group until necropsy of the dam and pups at weaning. I used the means, standard deviations, and sample sizes in the tables in the paper and reanalyzed the data using one-way ANOVAs followed by Dunnett's multiple comparisons test (GraphPad Prism version 7.00 for Windows, GraphPad Software, La Jolla California USA, www.graphpad.com). None of the effects reported as significant in the paper appear to be statistically significant upon re-analysis (see the figures that follow).

My interpretation of these data is that MPS dosing for 90 days or longer had no effect on growth, viability, fertility, fecundity or viability in the parent or offspring generations.

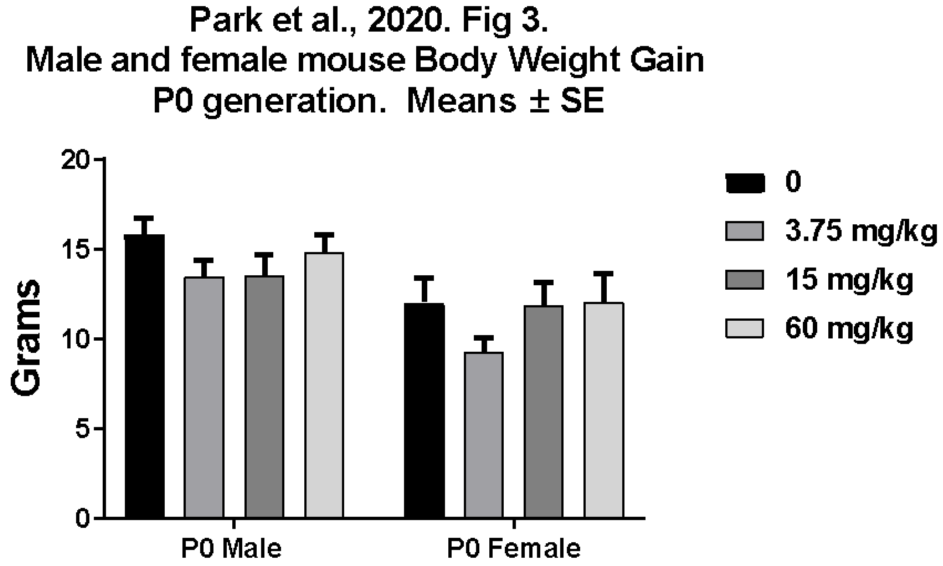

ANOVA summary	
F	0.4786
P value	0.7144
P value summary	ns
Significant diff. among means (P < 0.05)?	No
R squared	0.2641

Dunnett's multiple comparisons test	Mean Diff.	95.00% CI of diff.	Significant?	Summary	Adjusted P Value
0 vs. 3.75 mg/kg	2.600	-5.769 to 10.97	No	ns	0.5907
0 vs. 15 mg/kg	1.250	-7.119 to 9.619	No	ns	0.9046
0 vs. 60 mg/kg	0.5000	-7.869 to 8.869	No	ns	0.9920

Park et al., 2020. Fig 8, Section 3.7.
F1 postnatal day 21 Mouse Body Weight
Means ± SE

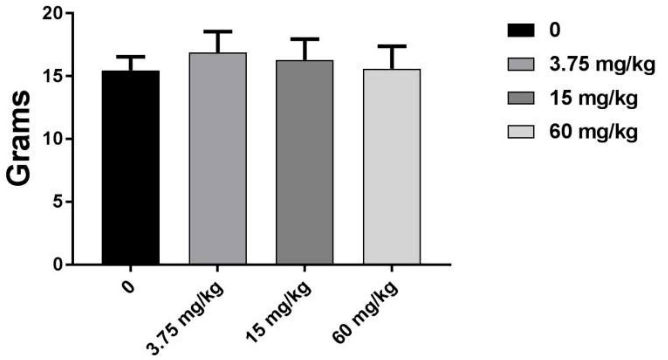

ANOVA summary	
F	0.1817
P value	0.9071
P value summary	ns
Significant diff. among means (P < 0.05)?	No
R square	0.03747

Dunnett's multiple comparisons test	Mean Diff.	95.00% CI of diff.	Significant?	Summary	Adjusted P Value
0 vs. 3.75 mg/kg	-1.45	-7.271 to 4.371	No	ns	0.8504
0 vs. 15 mg/kg	-0.84	-6.661 to 4.981	No	ns	0.9636
0 vs. 60 mg/kg	-0.14	-5.628 to 5.348	No	ns	0.9999

Park et al., 2020. Table 3.
F2 Mouse Birth Weight
Means ± SE
F2 PUPS n = 4 or 5 litters/group

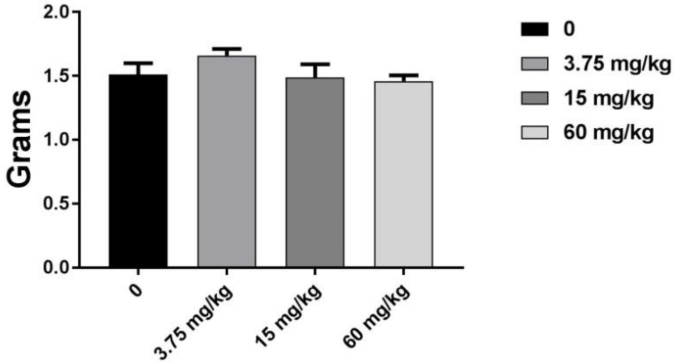

ANOVA summary	
F	1.334
P value	0.3031
P value summary	ns
Significant diff. among means (P < 0.05)?	No
R square	0.2223

Dunnett's multiple comparisons test	Mean Diff.	95.00% CI of diff.	Significant?	Summary	Adjusted P Value
0 vs. 3.75 mg/kg	-0.15	-0.429 to 0.129	No	ns	0.3838
0 vs. 15 mg/kg	0.02	-0.259 to 0.299	No	ns	0.9951
0 vs. 60 mg/kg	0.05	-0.2131 to 0.3131	No	ns	0.9237

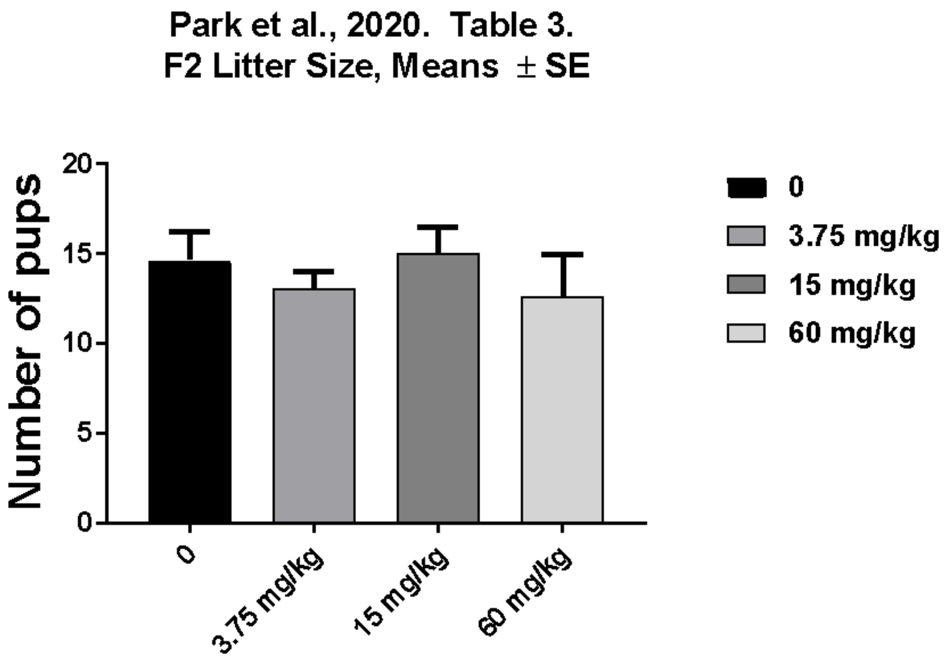

ANOVA summary	
F	0.4335
P value	0.7324
P value summary	ns
Significant diff. among means (P < 0.05)?	No
R squared	0.08500

Dunnett's multiple comparisons test	Mean Diff.	95.00% CI of diff.	Significant?	Summary	Adjusted P Value
0 vs. 3.75 mg/kg	1.600	-5.118 to 8.318	No	ns	0.8693
0 vs. 15 mg/kg	-0.4000	-7.118 to 6.318	No	ns	0.9973
0 vs. 60 mg/kg	2.000	-4.334 to 8.334	No	ns	0.7515

References

Amereh, F., Babaei, M., Eslami, A., Fazelipour, S., and Rafiee, M. (2020). The emerging risk of exposure to nano(micro)plastics on endocrine disturbance and reproductive toxicity: From a hypothetical scenario to a global public health challenge. *Environ Pollut* **261**, 114158.

Cagen, S. Z., Waechter, J. M., Dimond, S. S., Breslin, W. J., Butala, J. H., Jekat, F. W., Joiner, R. L., Shiotsuka, R. N., Veenstra, G. E., and Harris, L. R. (1999). Normal reproductive organ development in CF-1 mice following prenatal exposure to bisphenol A. *Toxicological Sciences* **50**(1), 36-44.

Hou, B., Wang, F., Liu, T., and Wang, Z. (2021). Reproductive toxicity of polystyrene microplastics: In vivo experimental study on testicular toxicity in mice. *J Hazard Mater* **405**, 124028.

Odet, F., Pan, W., Bell, T. A., Goodson, S. G., Stevans, A. M., Yun, Z., Aylor, D. L., Kao, C. Y., McMillan, L., de Villena, F. P.*, et al.* (2015). The Founder Strains of the Collaborative Cross Express a Complex Combination of Advantageous and Deleterious Traits for Male Reproduction. *G3 (Bethesda)* **5**(12), 2671-83.

Park, E. J., Han, J. S., Park, E. J., Seong, E., Lee, G. H., Kim, D. W., Son, H. Y., Han, H. Y., and Lee, B. S. (2020). Repeated-oral dose toxicity of polyethylene microplastics and the possible implications on reproduction and development of the next generation. *Toxicol Lett* **324**, 75-85.

Sagan, C. (1995). *The Demon-Haunted World: Science as a Candle in the Dark*. Random House.

Tyl, R. W., Myers, C. B., Marr, M. C., Castillo, N. P., Veselica, M. M., Joiner, R. L., Dimond, S. S., Van Miller, J. P., Stropp, G. D., Waechter, J. M.*, et al.* (2008a). One-generation reproductive toxicity study of dietary 17beta-estradiol (E2; CAS No. 50-28-2) in CD-1 (Swiss) mice. *Reproductive Toxicology* **25**(2), 144-160.

Tyl, R. W., Myers, C. B., Marr, M. C., Sloan, C. S., Castillo, N. P., Veselica, M. M., Seely, J. C., Dimond, S. S., Van Miller, J. P., Shiotsuka, R. N.*, et al.* (2008b). Two-generation reproductive toxicity study of dietary bisphenol A in CD-1 (Swiss) mice. *Toxicological Sciences* **104**(2), 362-384.

Tyl, R. W., Myers, C. B., Marr, M. C., Sloan, C. S., Castillo, N. P., Veselica, M. M., Seely, J. C., Dimond, S. S., Van Miller, J. P., Shiotsuka, R. S.*, et al.* (2008c). Two-generation reproductive toxicity evaluation of dietary 17beta-estradiol (E2; CAS No. 50-28-2) in CD-1 (Swiss) mice. *Toxicological Sciences* **102**(2), 392-412.

Wang, Y. (2003). Epididymal sperm count. *Curr Protoc Toxicol* **Chapter 16**, Unit16 6.
WHO (2010). WHO laboratory manual for the examination and processing of human semen - 5th ed. In (5TH ed. WHO Press, Geneva.

Xie, X., Deng, T., Duan, J., Xie, J., Yuan, J., and Chen, M. (2020). Exposure to polystyrene microplastics causes reproductive toxicity through oxidative stress and activation of the p38 MAPK signaling pathway. *Ecotoxicol Environ Saf* **190**, 110133.

Dr Jonathan J Powell, Head of Biomineral Research and Pro-VP Hughes Hall, University of Cambridge and CEO, NoBACZ Healthcare.

Date received: August 23, 2021

Microplastics and the Gut. Brief review of Li et al (2020), Deng et al (2017) and Deng et al (2020) as requested by Dr Scott Coffin of the California State Water Resources Control Board.

Authored by: Dr Jonathan J Powell, Head of Biomineral Research and Pro-VP Hughes Hall, University of Cambridge and CEO, NoBACZ Healthcare.

Conflicts of Interest. None. I have no plastics or microplastics interests directly or indirectly in my academic or commercial activities.

Scene setting. The author has studied particles in the gut since 1987- notably mineral particles with an emphasis on sub-micron sized particles but, from which, there should be many lessons learned to cross apply to plastic particle studies. In a review of particles in the gut, last year, we were critical of the lack of quality in animal models and animal experimentation in the area[1]. Many of those comments / criticisms have bearing on the papers here reviewed. Of particular note, particles may not adhere to conventional toxicology paradigms- for example, at increased concentrations they may agglomerate and lead to an effective *lower* exposure dose confounding interpretation. Key to any experimental interpretation is extensive characterization of the system and methodology- generally this is lacking.

Deng et al 2017

This is the slightly better of the 3 papers. Some effort is made to characterize the particles and their agglomeration although, for the latter, not under GI conditions (which is practically very difficult but may change everything). No attempt is made to assess how much is actually gavaged (versus that retained) but data suggest that at least some is. Gavage is stressful and deeply unphysiological- it alters GI physiology / permeability and in such bolus dosing it fails to replicate human exposure. The particles are green fluorescent and chief measurements of uptake are through the process of digestion of tissues and use of a fluorescent spectrophotometer. This would need careful validation (spiking recovery etc type processes to assess how tissue processing affects the validity of florescence analysis), which did not occur, and the control (background) fluorescence was subtracted but raw data not shown. Particle sizes were 5 and 20 microns which may be small compared to human exposure but are still very large for GI translocation (see Overall view below). Particles were spherical which may not represent the shards of human exposure. No attempt to cross reference dose to human exposure is made.

Epi-florescence is used to identify the particles in tissue and the authors then superimpose the image onto regular light microscopic H&E images as ‘representative

examples'. Green fluorescence has high background in the gut and control data are not shown. Representative images are fraught with issues[2].

Some association is reported for plastic particle exposure and hepatic inflammation and lipid droplet accumulation, but this is again through impressions with visualization and how robust this is unclear. Metabolic data do seem to suggest a dose response and appear plausible although have come in for criticism[3].

In summary, this paper suggests that large plastic particles *may* be taken up by the gut and may have detrimental effects. Both need confirming in very carefully controlled studies and relevance to human exposure understood.

Li et al 2020

Methodology descriptions and experimental control are so sparse that it's very difficult to make any sensible assessment of this paper. Particles were added to the feed and may well be closer in size to human exposure that in the paper above- both aspects are laudable. Unfortunately, no effort was made to characterize them in the diet or their state of dispersion. The high dose group seems very high and in terms of microbiome outcome it enhances diversity which is generally considered a 'good thing'- so this is not easily interpreted. One cytokine, IL-1 alpha, from a number, appears lower in serum in the control diet than the plastics-containing diets but how much of this is monkeys and typewriters? There's no real reasoning why this cytokine should be the target one and it's not clear what else was analysed and not reported. At the very least with 8 targets shown the p value threshold should really be 0.006 (not 0.01 as reported). The flow cytometry has no raw data associated with it which means it cannot be properly per reviewed. Ditto the immunofluorescence which, as noted above, is perilous without proper controls and careful quantitation. My impression is that this is a poorly controlled study with a shotgun approach and anything that differs is stuck into a paper.

Deng et al 2020

Here, plastic particles, with and without phthalate esters pre-adsorbed, and of some size-relevance to human exposure, were gavaged to animals. The concerns with gavage, and the dose and morphology relevance to humans, as noted above, remain. They report 'accumulation' in gut tissue but this is misleading as no work was undertaken to empty the lumen (anyway particles stick to the epithelial surface and such work is fraught with pitfalls using gross analysis of gut tissue). The polarized light microscopy work is actually very interesting but again, just representative images are shown which is a real shame as this could be a rich seam to mine with proper quantitative in situ analysis (ie as per ref 2 for example). Such large particles penetrating (translocating) the gut is a 'big statement' so confirming that these are in the tissue and not on (eg contamination / particle movement during preparation of tissue sections) needs careful work. Effects as measured seem to be mainly phthalate ester driven

although some evidence for increased intestinal permeability and inflammation by the microplastics is presented. The latter, again, by unconvincing representative images.

Overall view

Human oral exposure to microplastics is unlikely to be a good thing. But the question is, is it a bad thing and, if so, how bad? Poor studies are in many ways worse than no studies in addressing this (throwing large doses of most things at rats and analysing multiple parameters will undoubtedly reveal something). However, on the positive side, a number of techniques, approaches and outcomes may be refined for a future robust study. Gerhard Volkheimer (https://www.sciencedirect.com/science/article/abs/pii/S105435890860188X) and, later, Kate Carr, showed that at least *some* large particles are taken up by the gut, albeit very inefficiently. But may be plastic particles are better taken up- related to their hydrophobicity for example- by the gut than their counterpart inorganic / mineral particles (on which most reliable literature data are based)?. Worth checking. Human dose, size and morphology should be borne in mind. Ideally dose relates to cell dose as humans are exposed to[4] but this may be very tough to assess currently for plastics. The gross analysis technique is worth pursuing with but needs careful validation. Microscopy (polarized light is an interesting idea) should be used quantitatively and very carefully characterized / validated. Outcomes on (intestinal) permeability seem sensible as does inflammation, again undertaken quantitatively and carefully validated and described. Exposure must be dietary and again validated- both in terms of dispersion and intakes. Faecal analysis can be a useful real-time exposure measure.

References

(1) Gastrointestinal Absorption and Toxicity of Nanoparticles and Microparticles: Myth, Reality and Pitfalls explored through Titanium Dioxide. da Silva AB, Miniter M, Thom W, Hewitt RE, Wills J, Jugdaohsingh R, Powell JJ.Curr Opin Toxicol. 2020 Feb;19:112-120. doi: 10.1016/j.cotox.2020.02.007. Epub 2020 Feb 28.PMID: 32566805 Free PMC article

(2) Image-Based Cell Profiling Enables Quantitative Tissue Microscopy in Gastroenterology. John W Wills , Jack Robertson, Huw D Summers , Michelle Miniter, Claire Barnes, Rachel E Hewitt, Åsa V Keita, Johan D Söderholm, Paul Rees, Jonathan J Powell. Cytometry A. 2020 Dec;97(12):1222-1237. doi: 10.1002/cyto.a.24042. Epub 2020 May 23.

(3) Braeuning A (2018) Uptake of microplastics and related health effects: a critical discussion of Deng et al. Scientific reports 7:46687, 2017. Arch Toxicol 2018:1–2

(4) A Murine Oral-Exposure Model for Nano- and Micro-Particulates: Demonstrating Human Relevance with Food-Grade Titanium Dioxide. Riedle S, Wills JW, Miniter M, Otter DE, Singh H, Brown AP, Micklethwaite S, Rees P, Jugdaohsingh R, Roy NC, Hewitt RE, Powell JJ.Small. 2020 May;16(21):e2000486. doi: 10.1002/smll.202000486. Epub 2020 May 4

Dr. Aimen Farraj, U.S. EPA

(Dr. Aimed Farraj requested that their full review not be included in the supporting information)

Dr. Pauliina Damdimopoulou, Karolinska Institutet

Date Received: June 8, 2021

To: Scott Coffin, Research Scientist, California Environmental Protection Agency

Assessment carried out by: Assc Prof. Pauliina Damdimopoulou, with research team members Eleftheria Maria Panagiotou, Tianyi Li, Dr. Richelle Duque Björvang, and Dr. Valentina Di Nisio, Karolinska Institutet, Sweden

Background

Dr Scott seeks for expert opinion on studies detailing reproductive effects of MP *in vivo* in rodents by email to Dr Damdimopoulou on the 24th of April 2021. Damdimopoulou agrees to review the papers focusing on female reproduction together with her team. Scott asks for advice specifically on following topics:

- the strengths and limitations of the studies
- reliability of endpoints
- their biological relevance for humans
- the weight-of-evidence
- possibility to use the studies for non-regulatory threshold derivation

Materials

Luo et al. 2019 "*Maternal polystyrene microplastic exposure during gestation and lactation altered metabolic homeostasis in the dams and their F1 and F2 offspring*"

Hou et al. 2021 "*Polystyrene microplastics lead to pyroptosis and apoptosis of ovariangranulosa cells via NLRP3/Caspase-1 signaling pathway in rats*"

An et al. 2021 "*Polystyrene microplastics cause granulosa cell apoptosis and fibrosis inovary through oxidative stress in rats*"

Overall assessment

- *Luo et al. 2019*

This paper is focused on the impact of gestational MP exposure on gut microbiome, liver metabolism and metabolic syndrome in the offspring. These endpoints are not of direct relevance to female fertility.

- *Hou et al. 2021 and An et al. 2021*

These papers specifically address the impact of MP exposure on ovaries in rats and are therefore of direct relevance to female fertility.
After a careful evaluation, it is our collective conclusion that the two papers are based on the

same study and the results reporting is overlapping. In both studies, 32 female Wistar rats of6 weeks of age weighing ca 180 g were purchased from the same provider. The MP preparation are the same, as shown by the same images in Fig 1 in both papers. The data on serum AMH are the same in (Fig 5A in Hou et al. and Fig 2C in An et al.). In addition, data on oxidative stress markers reported seem very similar in both papers (Fig 3 in Hour et al. and Fig 3 in An et al.) Participating laboratories are the same, half of the authors are the same, ethical license is from the same university board, and the manuscripts were submittedand published around the same time. *Therefore, it is our strong recommendation to considerthese two papers as one experiment*. All points raised below are relevant to both reports.

SciRap

[Note: Dr. Damdimopulou's research team evaluated An et al 2021 and Hou et al 2021 using the SciRap criteria. Assessments for *in vivo* and *in vitro* experiments were completed separately. Complete assessments are available in excel format. Key figures for studies are below:]

An et al 2021

In vivo

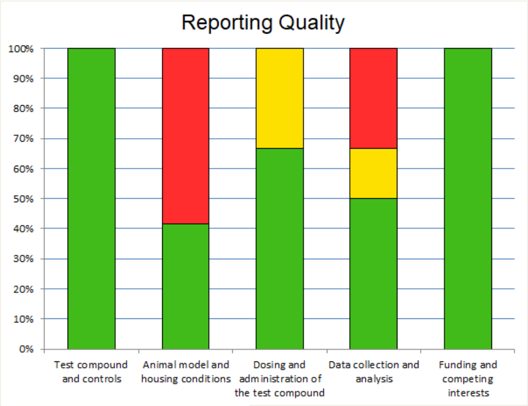

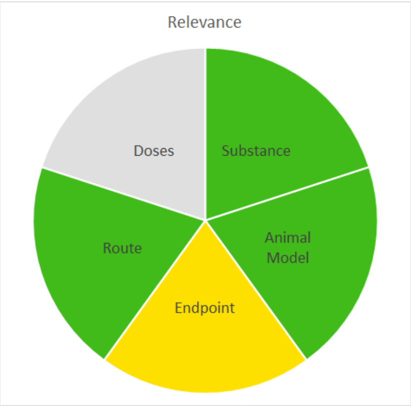

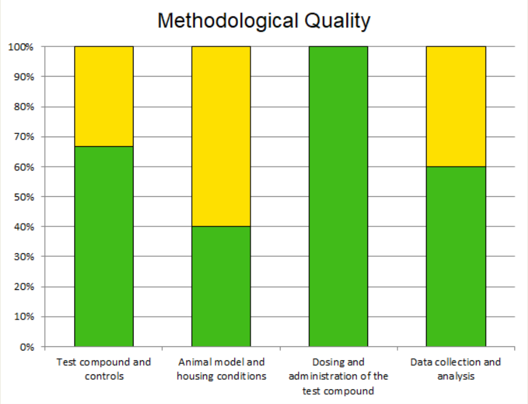

Supporting Information for Development and application of a health-based framework for informing regulatory action in relation to exposure of microplastic particles in California drinking water

In vitro

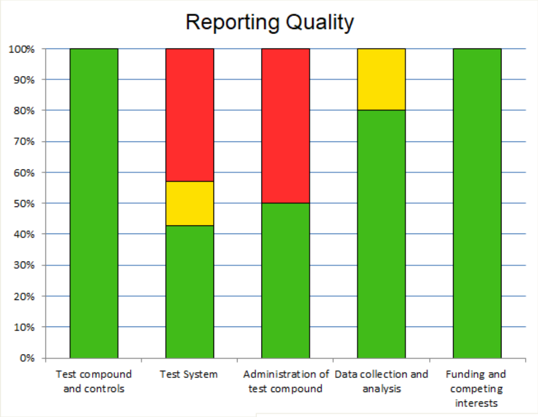

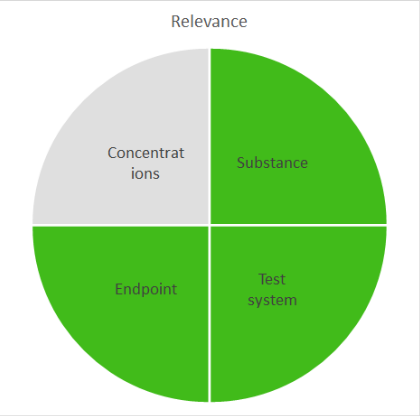

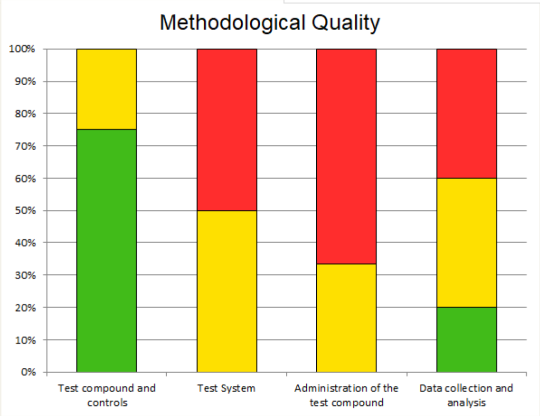

Supporting Information for Development and application of a health-based framework for informing regulatory action in relation to exposure of microplastic particles in California drinking water

Hou et al (2021)

In vivo

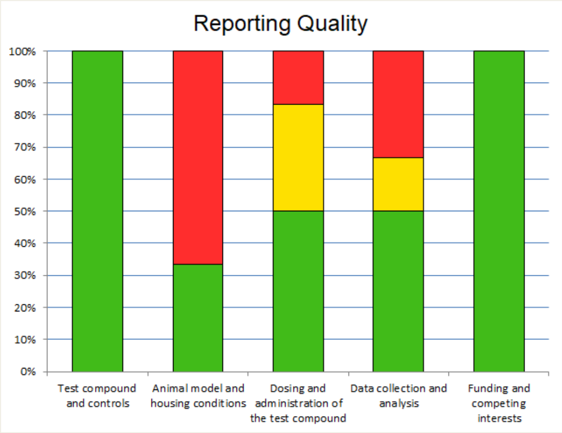

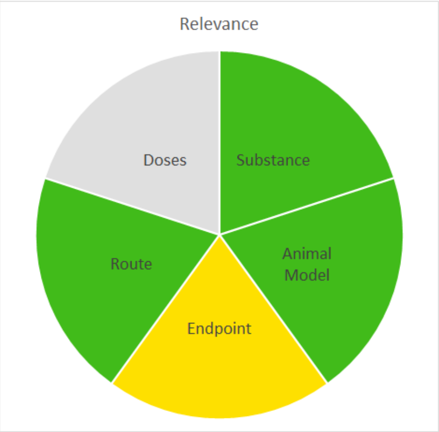

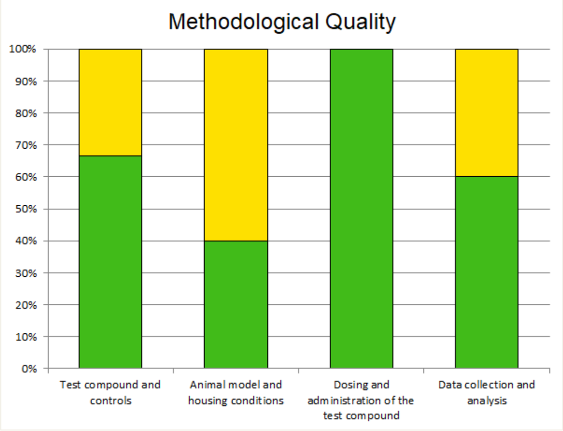

In vitro

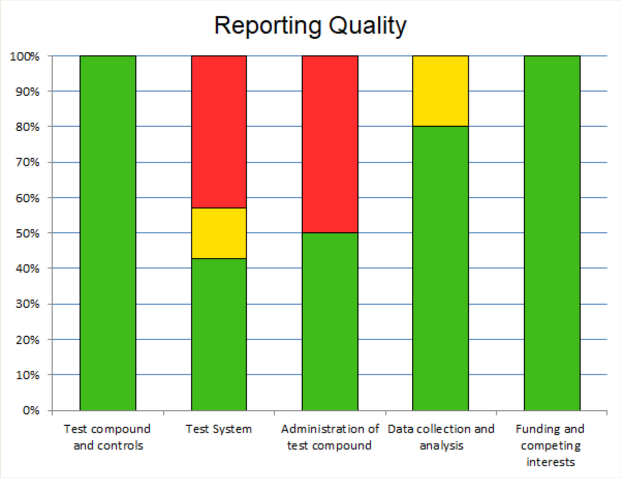

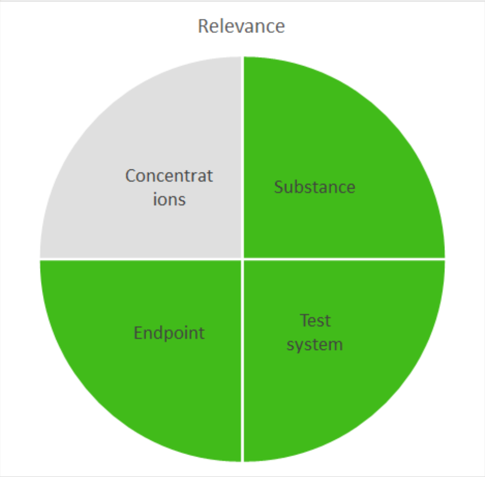

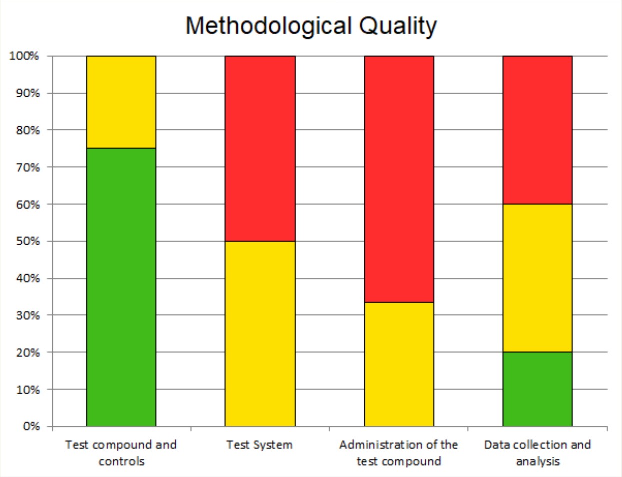

Detailed assessment of Hou et al. and An et al.

Dosing

For threshold derivation, accurate information on dosing is essential. We note that Hou et al.reports dosing as 0.015, 0.15 and 1.5 mg/d, while An et al. reports it as 0.015, 0.15 and 1.5 mg/kg/d. Considering that the papers likely report the same study, it remains unclear whether the doses were adjusted for body weight or not.

Neither study explains how the added MPs in drinking water were calculated to result in the given doses during the 90-day experiment. As the rats were juvenile at the start, and 4-5 months in the end, their weight will have increased during the exposure period. It is also not described how water consumption was measured to assure correct dosing. This is important, because adding a substance to the water can change palatability, leading to altered water intake, which will affect the dosing. If the doses were adjusted for body weight, like suggested in An et al., it is not explained how the increasing rat body weights were considered.

In conclusion, we find it impossible to judge based on the information given what the actual MP dosing in the experiments was. Therefore, the relevance to human situation is difficult toassess.

Animals

During acclimatization period, rats were housed individually, which constitutes a stress factorthat can affect behavior and hormonal status of the animals. However, it is not reported how the rats were housed during the exposure period.

Housing conditions (temperature, relative humidity, light-dark cycle) are appropriate and inagreement with the OECD guidelines.

There is no information on the housing materials and potential environmental enrichment materials, which could be a source of endocrine disrupting chemicals and MPs.

No information on feed is given. To assess the effects on reproductive toxicity, it is recommended to give specific diet with reduced phytoestrogen content to avoid possible interference.

No information on general signs of stress or toxicity are given. For example, body weight gain is not recorded and organ weights at necropsy are not reported.

Assessment of endpoints

Ovarian histology

The stock of non-growing follicles (or ovarian reserve) is established during early development, and follicles grow in a process called folliculogenesis to reach the ovulatory stage that releases mature oocytes for ovulation. Remnants of the post-ovulatory follicle become *corpus luteum*. Majority of follicles die through atresia. Follicles are not evenly distributed in the ovary. There are no internationally agreed and harmonized ways to carry out follicle counting and scoring, and therefore detailed description of the process is strictly essential to be able to interpret results. An et al. refer to two *ca* 20 years old publications as the source of their follicle classification. In these papers, growing follicles are defined under many separate categories, which is not done by An et al. Hou et al. mentions “primary and secondary follicles were counted as growing”, but in the figures they show antral follicles only (primary and secondary follicles are typically not antral).
According to OECD guideline 443 assessing reproductive toxicity, quantification of primordial, small growing follicles and corpora lutea in F1 generation is recommended. For the selection of ovarian sections, middle third of the ovaries should be used. It is recommended that every 20th section (or 100 micron interval) should be used, and in total, 10 individual sections should be evaluated. In the paper, “five visual fields were randomly selected” for histological evaluation, which is not the recommended systematic way to assess folliculogenesis.

Typically, rat ovaries would contain hundreds of growing follicles. An et al. (Fig 2B) and Hou et al. (Fig 2B) report on the average 6-12 growing follicles per group. It is difficult to judge what this number refers to due to lacking information on i) what was counted as growing follicles, ii) how many sections were evaluated per rat, iii) the fraction of ovary that was evaluated. The authors write in the materials and methods that “some ovaries were collected” and “some tissue obtained from ovaries was used”, which makes the evaluation oftheir data

even more difficult.

Authors refer to “follicle volume” and “thickness of granulosa cell layer” in the results but do not explain how it was measured or show the quantitative data.

Stage of estrus cycle was not recorded or taken into consideration in histological analyses.

Oxidative stress markers

The authors have measured markers of oxidative stress using “special kits”. No detail is given on the name, brand, or provider of these kits, and it is hence difficult to evaluate the data from these experiments. Overall, with the reservation of not being able to tell how the kits function, it seems that increasing MP exposure leads to increasing oxidative stress. It is unclear how many samples and replicas the assays are based on.

Western blot analyses

The authors show cut bands, when the standard is to disclose the entire blots so that reviewers can estimate product size and antibody specificity, among other things. It is unclear how many samples and replicas the assays are based on.

Immunostainings

The authors have not included negative controls, which makes the estimation of antibody specificity hard. On the other hand, the authors have included a quantification of the signal which positive as quantitative information is often difficult to obtain from immunostainings. However, details of the quantification process are not included, making assessment of the data challenging. It is unclear how many samples and replicas the assays are based on.

Flow cytometry

Authors do not include any controls in flow cytometry, and the plots in figures are not labelled. It is unclear how many samples and replicas the assays are based on.

ELISA

Kit performance is not explained. It is unclear how many samples and replicas the assays are based on.

Granulosa cell isolation and culture

It is not clear how many rats were used for GC isolation, and how many cultures and replicates were carried out.

Points to improve for next experiments

- Record food and water consumption, body weights, and signs of stress/toxicity during experiment.
- Report housing materials and consumables used, for example water bottle and cage material which are likely plastic.
- Record estrus cyclicity and collect the samples at the same stage of the cycle.
- Serum hormone measurements.
- Ovarian weights.
- Systematic quantitation of follicles and corpora lutea with a well described method for example following the suggestions made by OECD.
- Calculated the numbers of corpora lutea as a sign of recent ovulations.
- Increase animals per group- relatively low numbers of animals per group (n=8, compared to n>=10 suggested by OECD).
- Report measures taken to prevent MP contamination through equipment, air and clothing
- Consider chemical additives in the MPs to account for “particle effect” and chemical additive effects separately

Weight of evidence

Because the data available in the two papers are likely derived from the same cohort of rats, there is no possibility to carry out weight of evidence analysis that would require at least two independent studies.

Human relevance

Of the measured endpoints, AMH is the most relevant to the human situation. AMH is secreted by small growing follicles, and it therefore directly reflects the number of growing follicles. Serum AMH is used in reproductive medicine to evaluate the likelihood of success in retrieving a sufficient number of oocytes in ovarian hyperstimulation during infertility treatments. However, the value of AMH in informing about female fertility potential in the general population (i.e. not infertility treatment patients) is unclear. AMH is not a direct marker of ovarian reserve, as the authors claim.
Antral follicle counts could also be of relevance to humans as a marker of folliculogenesis. However, the authors of neither paper specifically counted antral follicles.

Strengths

Characterization of the MPs Proof
that MPs reach the ovary
Long exposure period of 90 days that covers the entire folliculogenesis in rats and therefore could inform on toxicity during the entire follicle growth period
Relatively high numbers of animals per group (n=8, compared to n>10 suggested by OECD)

Conclusions

There are insufficient independent studies to carry out WOE analysis.
Threshold derivation not possible because the dosing is not clearly explained.
Most relevant endpoint to humans is AMH.
Reporting of data suffers from absence of controls and lacking information on numbers of observations and replicates, which leads to low overall reliability.
Quality assessment using the SciRAP resource (http://www.scirap.org/) is attached.
Overall quality of the two papers (one study) is low.

Opinion on Key Characteristics

Key Characteristics for female reproductive toxicity are a good pragmatic way to identify and prioritize chemicals for testing. As such, fulfilling one key characteristic in one model is not necessarily sufficient to label a chemical as a reproductive toxicant but rather a sign to study the chemical further.

Dr. Jodi Flaws, University of Illinois

Date Received: May 6, 2021

Overall Analysis of Seven Reproductive Toxicity Studies

Studies considered: Xie et al. (2020); An et al. (2021); Amereh et al. (2020); Li et al. (2021); Hou et al (2021a); Hou et al. (2021b); Jin et al. (2021)

Several consistencies among the studies:

1. All seven studies show negative effects of polystyrene microplastic exposure on aspects of male or female reproductive function. Thus, the weight of evidence indicates that exposure to polystyrene microplastic negatively impacts several reproductive endpoints in rodents. This is because, there is:

   a. Consistency in showing that polystyrene microplastics decrease well known biomarkers of gamete health (decreased sperm counts and ovarian follicle counts; abnormal production of protein and steroid sex hormones; alterations in pathways that lead to oxidative stress and cell death in the gonads)

   b. Consistency in showing negative effects of polystyrene microplastics on reproductive outcomes in different species (rats and mice). Of note, many of the biomarkers are highly conserved among species, including rodents and humans. For example, sperm counts, sperm viability, follicle counts, reproductive hormone levels are used in rodent and human studies to assess the impact of chemical exposures on reproduction. Although some species differences in absolute gamete number and hormone levels exist, the same types of gametes and hormones are present in rodents and humans.

   c. Consistency in many of the selected doses of polystyrene microplastics in the studies. Of note, many of the doses are in the environmental range and could occur in human exposure. It is interesting that some of the studies actually show that the microplastics reach the gonads. It is also interesting that some of the studies report non-monotonic dose effects, which are commonly observed in studies on endocrine disrupting chemicals.

   d. Consistency in the pathways that are disturbed by microplastic exposure. Several of the studies show that polystyrene microplastic exposure leads to changes in pathways that control oxidative stress and apoptosis. These pathways are highly conserved among species, including rodents and humans.

   e. Consistency in the methods used to assess the effects of polystyrene microplastics on reproductive endpoints. The selected histological evaluations, sperm counts/viability assays, follicle count assays, hormone assays, measures

of cell death/oxidative stress, and western blots are all methods that have been validated and used in many labs. The same types of methods/assays are used in rodents and humans.

Response to specific questions:

Which of the studies (if any) are most reliable for dose-response?

“As far as studies with good dose response information, I would focus on An et al and Amereh et al.”

What are your thoughts on the limitations of these studies in the larger context of how they would affect the hazard identification?

“ I do not think that I have the expertise to comment on the questions around hazard identification. Those are more risk assessment issues and I don’t have that experience.”

As far as my opinion on key characteristics for male reproductive toxicants, I think the ones in the EHP report are good. Hope this helps.

What is your opinion on the key characteristics for identifying male reproductive toxicants outlined in this perspective: https://ehp.niehs.nih.gov/doi/10.1289/EHP5045?

As far as my opinion on key characteristics for male reproductive toxicants, I think the ones in the EHP report are good

Could you provide feedback in regards to uncertainties suggested by an additional reviewer:

Dr. Earl Gray (US EPA) wrote that Amereh et al (2020) had small sample sizes (n = 6) and did use reliable sperm count methods or reporting. Specifically, Dr. Gray writes,

"The sperm “count” data are reported as sperm concentration per mL but not as total sperm per epididymis. Since an undefined piece of the epididymis was taken for assessment it is not clear what percent of the total epididymis was sampled it is not possible to determine how the reported value relates to total sperm numbers in the epididymis. It is also not possible to compare these results to those reported in the literature on mouse sperm counts."

"I DID NOT DO SAMPLE SIZE ESTIMATES, BUT BASED ON YOUR ESTIMATES, I THINK THE STUDY IS ADEQUATELY POWERED. EARL HAS A POINT, BUT MANY STUDIES ANALYZE SPERM IN THE SAME WAY. I WOULD NOT SAY THAT YOU CAN BASE CONCLUSIONS JUST ON THIS STUDY, BUT IT IS PART OF A LARGER PICTURE CONSISTENTLY SHOWING EFFECTS OF MICROPLASTICS ON SPERM. ALL THE OTHER STUDIES SHOW THE SAME THING BASICALLY."

Do you believe the sperm count data from Amereh et al (2020) are reliable? Dr. Gray suggests Xie et al (2020) may have the most reliable sperm count data, however the dose-response relationship is not as 'neat' as in Amereh et al (2020).

"I BELIEVE THEY ARE RELIABLE WHEN TAKEN IN CONTEXT WITH OTHER STUDIES THAT SHOW THE SAME EFFECTS OF MICROPLASTICS ON SPERM. ALL THE STUDIES ARE CONSISTENT IN SHOWING NEGATIVE EFFECTS OF MICROPLASTICS ON SPERM. I AGREE THAT XIE HAS REALLY GOOD SPERM ANALYSIS. I ALSO AGREE THAT THE DOSE RESPONSE IN THAT STUDY IS NOT NEAT. SOMETIMES EXPOSURES PRODUCE ABNORMAL DOSE RESPONSES. I WOULD SAY THAT YOU HAVE TO LOOK AT THE STUDIES AS A WHOLE."

Additionally, do you believe that any of the other male reproductive toxicity studies used reliable sperm count methods/reporting?

"FOLLICLE COUNTS AND AMH LEVELS"

Supplementary References

Belle, GV, and SP Millard. 1998. "STRUTS: Statistical Rules of Thumb." *Seattle, WA: University Of.*

Chapin, R.E.; Sloane, R.A.; Hasemann, J.K.: The relationships among reproductive endpoints in swiss mice, using the reproductive assessment by continuous breeding database. Fundam. Appl. Toxicol. 38(2) (1997b), 129-142.

Gray, L.; Ostby, J.; Ferrell, J.; Sigmon, R.; Cooper, R.; Linder, R.; Rehnberg, G.; Goldman, J.; Laskey, J.: Correlation of sperm and endocrine measures with reproductive success in rodents. In: Burger, E.J.:Progress in clinical and biological research.302.New York:Liss 1989, 193-209.

Hou, Baolian, Fangyi Wang, Tao Liu, and Zhiping Wang. 2021. "Reproductive Toxicity of Polystyrene Microplastics: In Vivo Experimental Study on Testicular Toxicity in Mice." *Journal of Hazardous Materials* 405 (March): 124028. https://doi.org/10.1016/j.jhazmat.2020.124028.

Mangelsdorf, I, and J Buschmann. 2002. "Extrapolation from Results of Animal Studies to Humans for the Endpoint Male Fertility," 151.

Meistrich, M.L.; Brown, C.C.: Estimation of the increased risk of human infertility from alterations in semen characteristics. Fertil. Steril. 40 (1983), 220-230.

Morrissey, Richard E, Bernard A Schwetz, JAMES C LAMB IV, Monica D Ross, Janet L Teague, and Richard W Morris. 1988. "Evaluation of Rodent Sperm, Vaginal Cytology, and Reproductive Organ Weight Data from National Toxicology Program 13-Week Studies." *Toxicological Sciences* 11 (1): 343–58.

Moore, J.A.; Daston, G.P.; Faustman, E.; Golub, M.S.; Hart, W.L.; Hughes, C.; Kimmel, C.A.; Lamb, J.C.; Schwetz, B.A.; Scialli, A.R.: An evaluative process for assessing human reproductive and developmental toxicity of agents. Reprod.Toxicol. 9(1) (1995), 61-95.

Rohatgi, Ankit. 2014. "WebPlotDigitizer User Manual Version 3.4." *URL Http://Arohatgi. Info/WebPlotDigitizer/App*, 1–18.

EPA :Guidelines for reproductive toxicity risk assessment. EPA630/R-96/009. September 1996, US EPA 1996.

Whitlock, Michael, and Dolph Schluter. 2015. "The Analysis of Biological Data: Textbook and Website."

Xie, Xiaoman, Ting Deng, Jiufei Duan, Jing Xie, Junlin Yuan, and Mingqing Chen. 2020. "Exposure to Polystyrene Microplastics Causes Reproductive Toxicity through Oxidative Stress and Activation of the P38 MAPK Signaling Pathway." *Ecotoxicology and Environmental Safety* 190 (March): 110133. https://doi.org/10.1016/j.ecoenv.2019.110133.

Amereh, F., Babaei, M., Eslami, A., Fazelipour, S., and Rafiee, M. (2020). The emerging risk of exposure to nano(micro)plastics on endocrine disturbance and reproductive toxicity: From a hypothetical scenario to a global public health challenge. *Environ Pollut* **261**, 114158.

Cagen, S. Z., Waechter, J. M., Dimond, S. S., Breslin, W. J., Butala, J. H., Jekat, F. W., Joiner, R. L., Shiotsuka, R. N., Veenstra, G. E., and Harris, L. R. (1999). Normal reproductive organ development in CF-1 mice following prenatal exposure to bisphenol A. *Toxicological Sciences* **50**, 36-44.

Gray, L. E. (2017). Biological relevance of reductions in sperm counts and testis weight in the male rat to fertility and fecundity: Internal Report to NCEA, ORD, pp. 1-24. EPA, EPA, NHEERL, DTD, RTB

Hou, B., Wang, F., Liu, T., and Wang, Z. (2021). Reproductive toxicity of polystyrene microplastics: In vivo experimental study on testicular toxicity in mice. *J Hazard Mater* **405**, 124028.

Meistrich, M. L. (1989). Calculation of the incidence of infertility in human populations from sperm measures using the two-distribution model. *Progress in clinical and biological research* **302**, 275-85; discussion 286-90.

Odet, F., Pan, W., Bell, T. A., Goodson, S. G., Stevans, A. M., Yun, Z., Aylor, D. L., Kao, C. Y., McMillan, L., de Villena, F. P., and O'Brien, D. A. (2015). The Founder Strains of the Collaborative Cross Express a Complex Combination of Advantageous and Deleterious Traits for Male Reproduction. *G3 (Bethesda)* **5**, 2671-83.

Park, E. J., Han, J. S., Park, E. J., Seong, E., Lee, G. H., Kim, D. W., Son, H. Y., Han, H. Y., and Lee, B. S. (2020). Repeated-oral dose toxicity of polyethylene microplastics and the possible implications on reproduction and development of the next generation. *Toxicol Lett* **324**, 75-85.

Sagan, C. (1995). *The Demon-Haunted World: Science as a Candle in the Dark*. Random House.

Tyl, R. W., Myers, C. B., Marr, M. C., Castillo, N. P., Veselica, M. M., Joiner, R. L., Dimond, S. S., Van Miller, J. P., Stropp, G. D., Waechter, J. M., and Hentges, S. G. (2008a). One-generation reproductive toxicity study of dietary 17beta-estradiol (E2; CAS No. 50-28-2) in CD-1 (Swiss) mice. *Reproductive Toxicology* **25**, 144-160.

Tyl, R. W., Myers, C. B., Marr, M. C., Sloan, C. S., Castillo, N. P., Veselica, M. M., Seely, J. C., Dimond, S. S., Van Miller, J. P., Shiotsuka, R. N., Beyer, D., Hentges, S. G., and Waechter, J. M. (2008b). Two-generation reproductive toxicity study of dietary bisphenol A in CD-1 (Swiss) mice. *Toxicological Sciences* **104**, 362-384.

Tyl, R. W., Myers, C. B., Marr, M. C., Sloan, C. S., Castillo, N. P., Veselica, M. M., Seely, J. C., Dimond, S. S., Van Miller, J. P., Shiotsuka, R. S., Stropp, G. D., Waechter, J. M., and Hentges, S. G. (2008c). Two-generation reproductive toxicity evaluation of dietary 17beta-estradiol (E2; CAS No. 50-28-2) in CD-1 (Swiss) mice. *Toxicological Sciences* **102**, 392-412.

Ulbrich, B.; Palmer, A.K.: Detection of effects on male reproduction - A literature survey. J. Am. Coll. Toxicol. 14(4) (1995), 293-327.

Wang, C.; Chan, Y.W.; Matthew, N.G.; William, W.K.SO; Tsol, W.L.; Lo, T.; Leung, A.: Diagnostic value of sperm function tests and routine semen analyses in fertile and infertile men. J. Androl. 9 (1988), 384-389.

Wang, Y. (2003). Epididymal sperm count. *Curr Protoc Toxicol* **Chapter 16**, Unit16 6.

WHO (2010). WHO laboratory manual for the examination and processing of human semen - 5th ed. WHO Press, Geneva.

Xie, X., Deng, T., Duan, J., Xie, J., Yuan, J., and Chen, M. (2020). Exposure to polystyrene microplastics causes reproductive toxicity through oxidative stress and activation of the p38 MAPK signaling pathway. *Ecotoxicol Environ Saf* **190**, 110133.
